# Insights into longevity and virus-driven adaptation from *Myotis* bat genomes

**DOI:** 10.1038/s41586-026-10932-7

**Published:** 2026-08-26

**Authors:** Juan M. Vazquez, M. Elise Lauterbur, Saba Mottaghinia, Léa Gaucherand, Sarah Maesen, Michael Singer, Sarah Villa, Melanie Bucci, Devaughn Fraser, Genavieve Gray-Sandoval, Zeinab R. Haidar, Melissa Han, William Kohler, Tanya M. Lama, Amandine Le Corf, Clara Loyer, Dakota McMillan, Stacy Li, Johnathan Lo, Carine Rey, Samantha L. R. Capel, Kathleen Slocum, Melissa Sui, William Thomas, Janet Debelak Tyburec, Rachel Brem, Richard Miller, Michael Buchalski, Jose Pablo Vazquez-Medina, Sébastien Pfeffer, Lucie Etienne, David Enard, Peter H. Sudmant

**Affiliations:** 1 Department of Integrative Biology, University of California, Berkeley, Berkeley, CA USA; 2 Department of Ecology and Evolutionary Biology, University of Arizona, Tucson, AZ USA; 3 Centre International de Recherche en Infectiologie (CIRI), Inserm U1111, Université Claude Bernard Lyon 1, CNRS UMR5308, Ecole Normale Supérieure ENS de Lyon, Université de Lyon, Lyon, France; 4 Université de Strasbourg, Architecture et Réactivité de l’ARN, Institut de Biologie Moléculaire et Cellulaire du CNRS, Strasbourg, France; 5 Department of Molecular and Cellular Biology, University of California, Berkeley, Berkeley, CA USA; 6 Wildlife Genetics Research Unit, Wildlife Health Laboratory, California Department of Fish and Wildlife, Sacramento, CA USA; 7 Department of Biology, California State Polytechnic University, Humboldt, Arcata, CA USA; 8 Department of Pathology and Clinical Laboratories, University of Michigan, Ann Arbor, MI USA; 9 Department of Biological Sciences, Smith College, Northampton, MA USA; 10 Department of Science and Biotechnology, Berkeley City College, Berkeley, CA USA; 11 Center for Computational Biology, University of California, Berkeley, Berkeley, CA USA; 12 Bat Conservation International, Austin, TX USA; 13 Department of Plant and Microbial Biology, University of California, Berkeley, Berkeley, CA USA; 14 Department of Ecology and Evolution, Stony Brook University, Stony Brook, NY USA; 15 Bat Survey Solutions, Tucson, AZ USA; 16 Present Address: Department of Biology, Pennsylvania State University, State College, PA USA; 17 Present Address: Department of Biology, University of Vermont, Burlington, VT USA; 18 Present Address: Max Delbrück Center for Molecular Medicine in the Helmholtz Association, Berlin, Germany; 19 Present Address: Wildlife Diversity Program, Wildlife Division, Connecticut Department of Energy and Environmental Protection, Burlington, CT USA; 20 Present Address: Colossal Biosciences, Dallas, TX USA

**Keywords:** Evolutionary genetics, Innate immunity, Functional genomics, Genome evolution, Genetic variation

## Abstract

The genus *Myotis* is one of the largest clades of bats, and it exhibits some of the most extreme variation in lifespans among mammals, alongside unique adaptations to viral tolerance and immune defence^1–3^. Here, to study the evolution of these phenotypes, we generated cell lines and near-complete genome assemblies for eight closely related *Myotis* species. Using genome-wide screens of positive selection, analyses of structural variation and functional experiments in primary cells, we identify patterns of adaptation contributing to longevity, cancer resistance and viral interactions. We demonstrate distinct modes of adaptation to DNA and RNA viruses compared with all other mammals, with bats exhibiting genome-wide over-representation of positive selection for DNA-virus-interacting proteins and elevated rates of copy-number variation for RNA-virus-interacting proteins. Characterization of *Myotis*-specific duplications of the key immune factor *EIF2AK2* (also known as *PKR*) reveals multiple ancient segregating trans-species copy-number polymorphisms. We show that the recurrent evolution of longevity seen in *Myotis* is associated with positive selection in cancer pathways, and demonstrate a unique response to DNA damage in primary cells of the long-lived *Myotis* *lucifugus*. Together, our results suggest that bats’ remarkable longevity and immunity are linked through pleiotropic adaptations to viruses and ageing-related disease.

## Abstract (web summary)

Comparative and functional analyses of *Myotis* bats uncover unique patterns of adaptation in virus-interacting proteins and longevity-associated pathways, linking two hallmarks of bat biology.

## Main

**Fig. 1:**
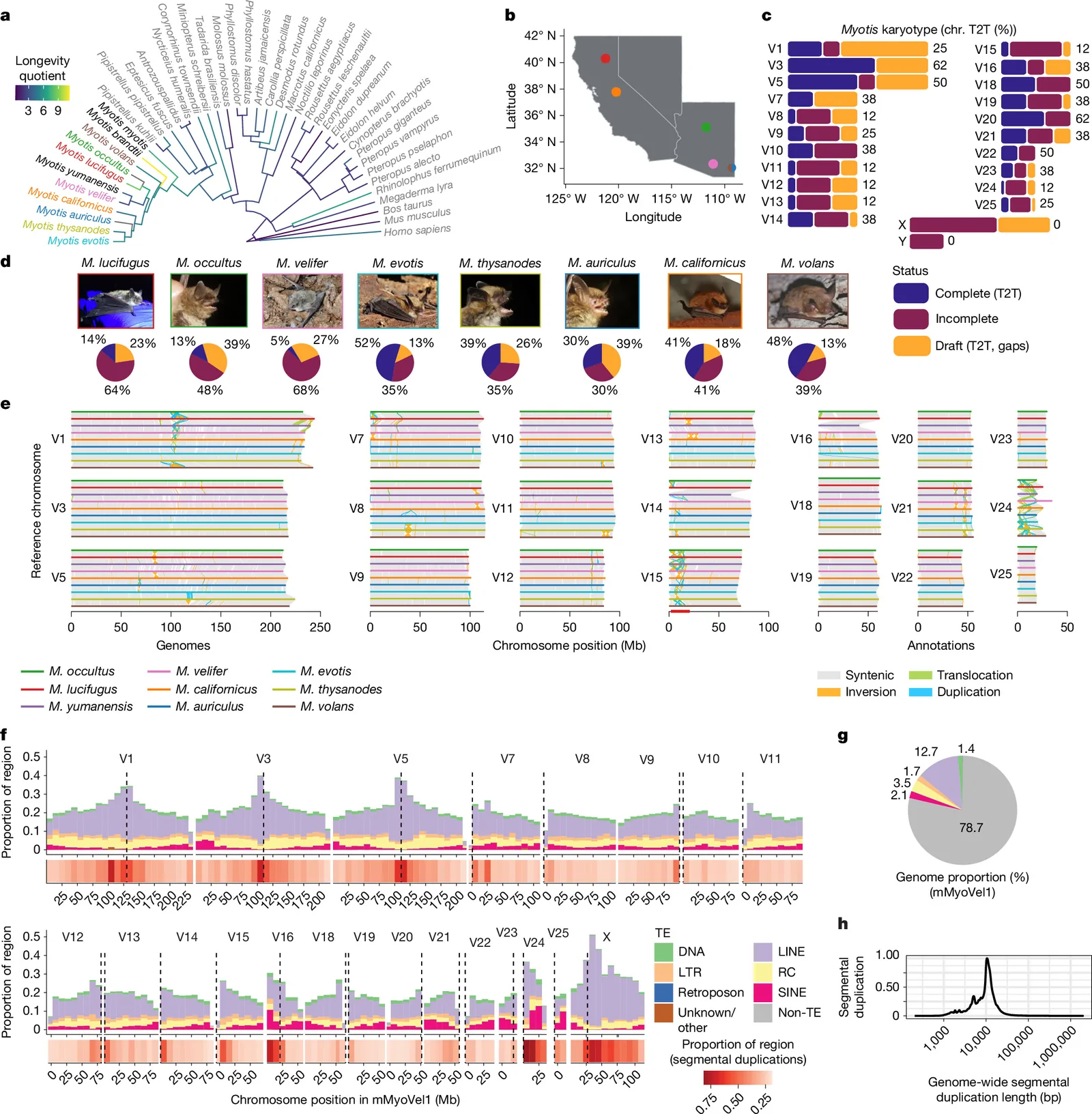
Near-complete reference assemblies reveal a varied structural variation landscape across nine Nearctic *Myotis* species. **a**, Phylogeny of Nearctic *Myotis* bats in this study, including outgroup species of bat, cow, mouse and human. Branches are coloured by their estimated longevity quotient, a ratio of observed-to-expected lifespan^1^. **b**, Map of capture sites in Arizona and California for samples generated in this study; each dot colour indicates the species. **c**, The completion status of each chromosome in assembly. The percentages next to the ideograms indicate the proportion of T2T-assembled chromosomes across species. **d**, The completion status of all chromosomes within each assembly, with representative images for the species shown above. For **c** and **d**, ‘complete (T2T) status’ indicates that a chromosome is fully assembled T2T without gaps; ‘draft (T2T, gaps)’ status indicates that a chromosome is fully scaffolded with both telomeres, but has one or more gaps in the assembly; ‘incomplete’ status indicates that a chromosome was positively identified, but was not scaffolded from telomere to telomere (contains only one telomere).** e**, Synteny between chromosomes of nine *Myotis* species showing syntenic regions (grey), inversions (orange), translocations (green) and duplications (blue). The red bar below chromosome V15 (*x* axis) indicates a locus of approximately 10 Mb where introgression was recently described in other *Myotis*^18^.** f**, The distribution of TEs (top) and segmental duplications (red heat map, bottom) in *M. velifer*. Putative locations of centromeres are denoted by the dotted lines. **g**, The overall genomic proportions of TEs in *M. velifer*. **h**, Histogram of segmental duplication size distributions genome wide in *M. velifer*. LINE, long interspersed nuclear element; LTR, long terminal repeat; RC, rolling circle transposon; SINE, short interspersed nuclear element. Images are from iNaturalist: Juan Cruzado Cortés (*M. californicus*, *M. volans*, *M. occultus*,* M. auriculus* and* M. thysanodes*) under a CC BY SA 4.0 licence; and Marlo Perdicas (*M. lucifugus*), Ansil B. R. (*M. velifer*) and Issac Krone (*M. evotis*) under a CC BY 4.0 licence.

Bats (order Chiroptera) represent approximately 20% of all known mammalian species and are one of the most phenotypically diverse clades of mammals^4^. Since their emergence 60 million years ago^5^, many bat lineages have independently evolved a wide variety of life history strategies and phenotypic traits, including exceptional longevity, viral tolerance and immune defences^2,3^. Systems in which shared traits have evolved de novo multiple times are powerful resources for dissecting the genetic basis of phenotypes. The largest genus of bats, *Myotis*, emerged approximately 33 million years ago^6^ and encompasses over 139 described species spanning 6 continents and a wide range of ecological niches^7^. *Myotis* species demonstrate some of the most extreme variation in lifespan among mammals^1,8^, including a sixfold difference in lifespan between the longest-lived species^9^ (*Myotis brandtii*, 42 years; Fig. 1a) and the shortest-lived species^10^ (*Myotis nigricans*, 7 years), which diverged approximately 10.6 million years ago^11^. Moreover, *Myotis* species are representative of bats’ notable immune mechanisms that enable viral tolerance and pathogen resistance^12^ contributing to their role as key zoonotic reservoirs^2,13^.

To study how longevity and infectious disease resistance have evolved in *Myotis*, we used an integrated field-to-functional-genomics approach to assemble near-complete genome assemblies for eight *Myotis* species with primary cell culture resources for functional validation. We identify copy-number-variable genes associated with RNA viral tolerance and stress response, as well as a trans-species copy number polymorphism of a key immune factor, *PKR*. Consistent with the extreme lifespans of *Myotis* relative to its body size, we identified selective evolutionary signatures in genes associated with longevity- and cancer-related processes. In contrast to humans and other primates, in which virus adaptation is driven by interactions with RNA viruses, we find that modes of virus adaptation in bats differ between DNA and RNA viruses. Together, our results highlight pleiotropic adaptations contributing to the lifespan and immune phenotypes of *Myotis* bats.

## Eight near-complete *Myotis* genome assemblies

**Extended Data Fig. 1:**
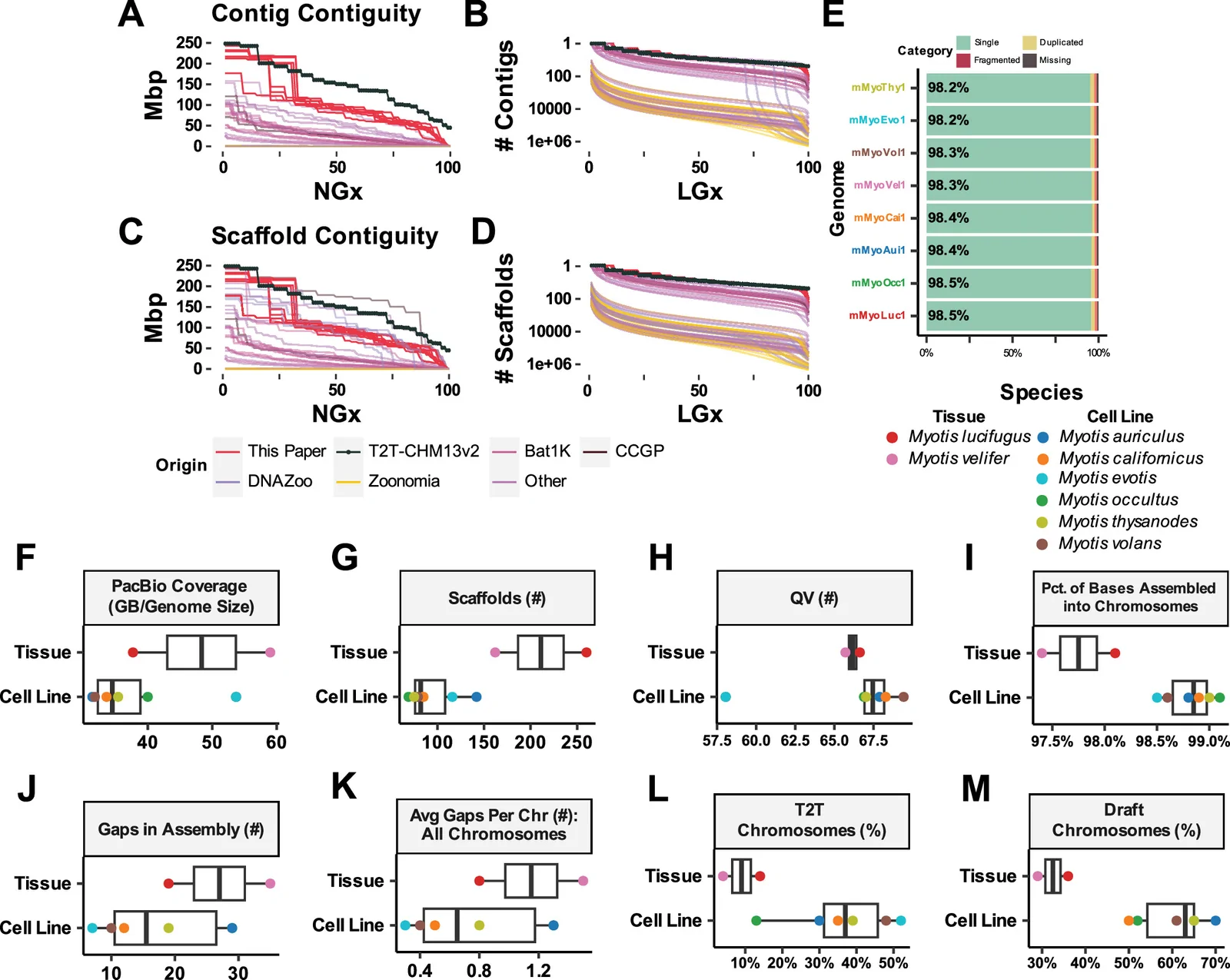
Contiguity statistics for 8 novel *Myotis* genomes. **A**) NG-decay plot for contig-level assemblies of bat genomes used in this paper. **B**) LG-decay plot for contig-level assemblies of bat genomes used in this paper. **C**) NG-decay plot for scaffold-level assemblies of bat genomes used in this paper. **D**) LG-decay plot for scaffold-level assemblies of bat genomes used in this paper. The invariance of the curves in **A-D** corresponding to the genomes generated in this paper is due to the high-contiguity of contigs assembled by *hifiasm-hic*; chromosome-level scaffolds were largely unaffected by scaffolding efforts. **E**) Mammalian BUSCO scores for annotations generated for the 8 new *Myotis* genomes. **F-M**) Genome assembly metric comparisons between assemblies derived from tissues or from primary cell lines. While assembly metrics do not correlate with coverage of PacBio HiFi sequencing (**F**), assemblies derived from primary cell lines do show improved statistics.

**Extended Data Fig. 2:**
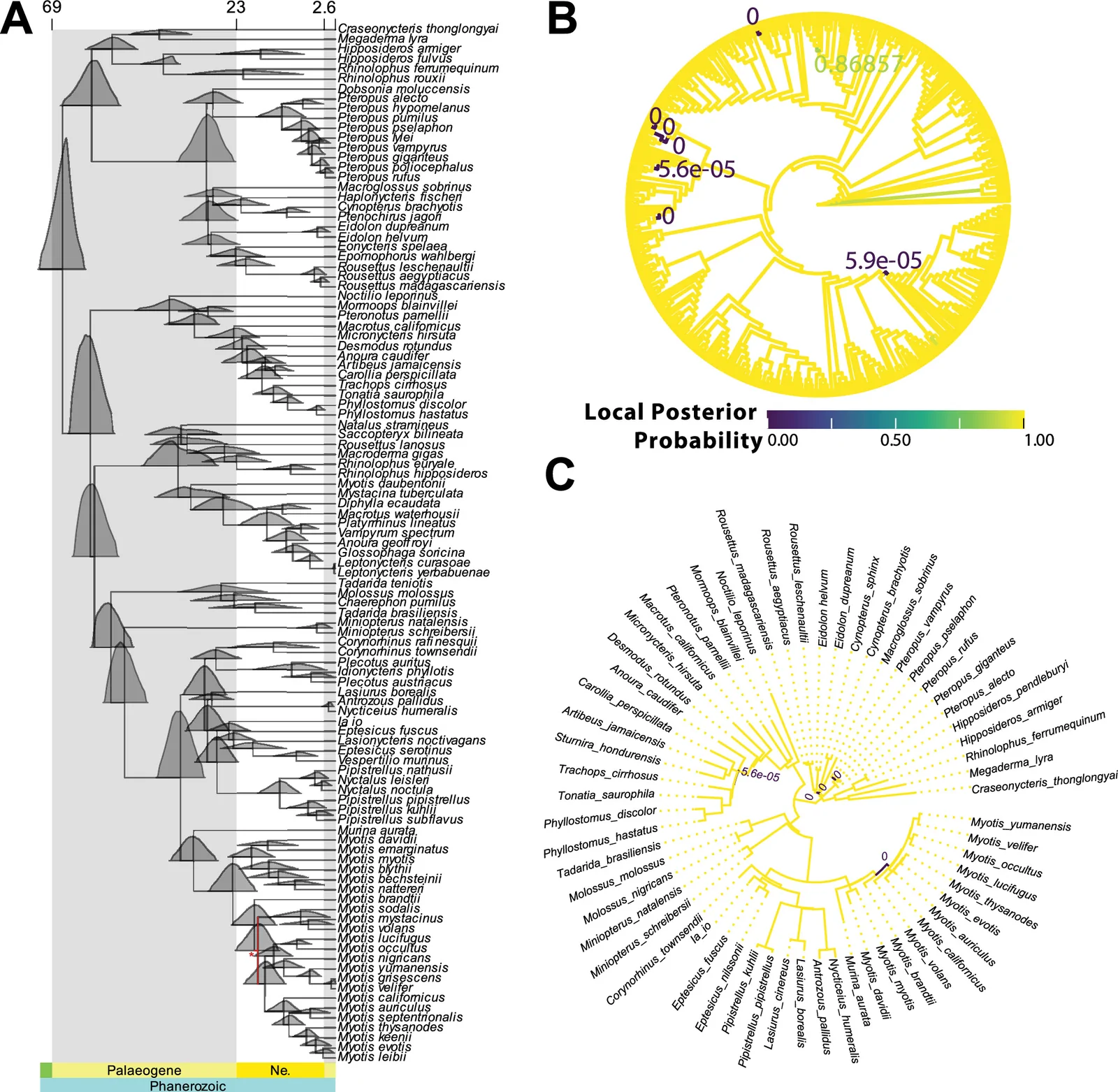
Estimated node divergence times for Chiroptera and ASTRAL-IV scores. **A**) Distribution of time estimates for nodes across the full fossil-calibrated Chiroptera phylogeny. **B**) ASTRAL-IV scored phylogeny of all mammalian genomes used in this study. **C**) ASTRAL-IV scored phylogeny of Chiroptera. For **B-C**, the local posterior probability (LPP) scores of nodes under <90% are shown on the tree; the branch at nearctic *Myotis* root with low bootstrap support is highlighted in red in **A**.

We collected skin punches and derived primary cell lines from several North American (nearctic)^14^ species (Fig. 1a,b,d), including from one of the longest-lived bats, *M. lucifugus*^15^. Using these cell lines and flash-frozen tissues, we generated de novo haplotype-resolved, chromosome-scale genome assemblies for eight species (Fig. 1c,d and Extended Data Fig. 1) using a combination of long-read PacBio HiFi sequencing and HiC scaffolding. These genomes are highly contiguous and near complete, with an average of 98.6% (98.1–99%) of nucleotides assembled into the 22–23 syntenic^16^ chromosomal scaffolds; an average quality value score of 66; and among the highest contig NG50 values of any Chiropteran genome thus far (where NG50 is the length of the shortest contig in the ordered set of longest contigs making up at least 50% of the total assembly length; Extended Data Fig. 1 and Supplementary Table 1). We identified an average of 20,869 protein coding genes with mammalian homologues per genome, with BUSCO^17^ scores ranging from 98.2% to 98.5% (Extended Data Fig. 1e), which we used to build a time-calibrated, maximum-likelihood tree of Chiroptera (Extended Data Fig. 2a and Supplementary Table 1). Across all eight genomes, each autosome has been completely assembled telomere-to-telomere (T2T) in at least one species (Fig. 1c); within assemblies, 29–70% of chromosomes are fully assembled with an average of less than one gap per chromosome (Fig. 1d and Supplementary Table 1). Overall, these fully annotated genomes represent some of the most contiguous mammalian assemblies thus far.

## Abundant structural variation in *Myotis*

**Extended Data Fig. 3:**
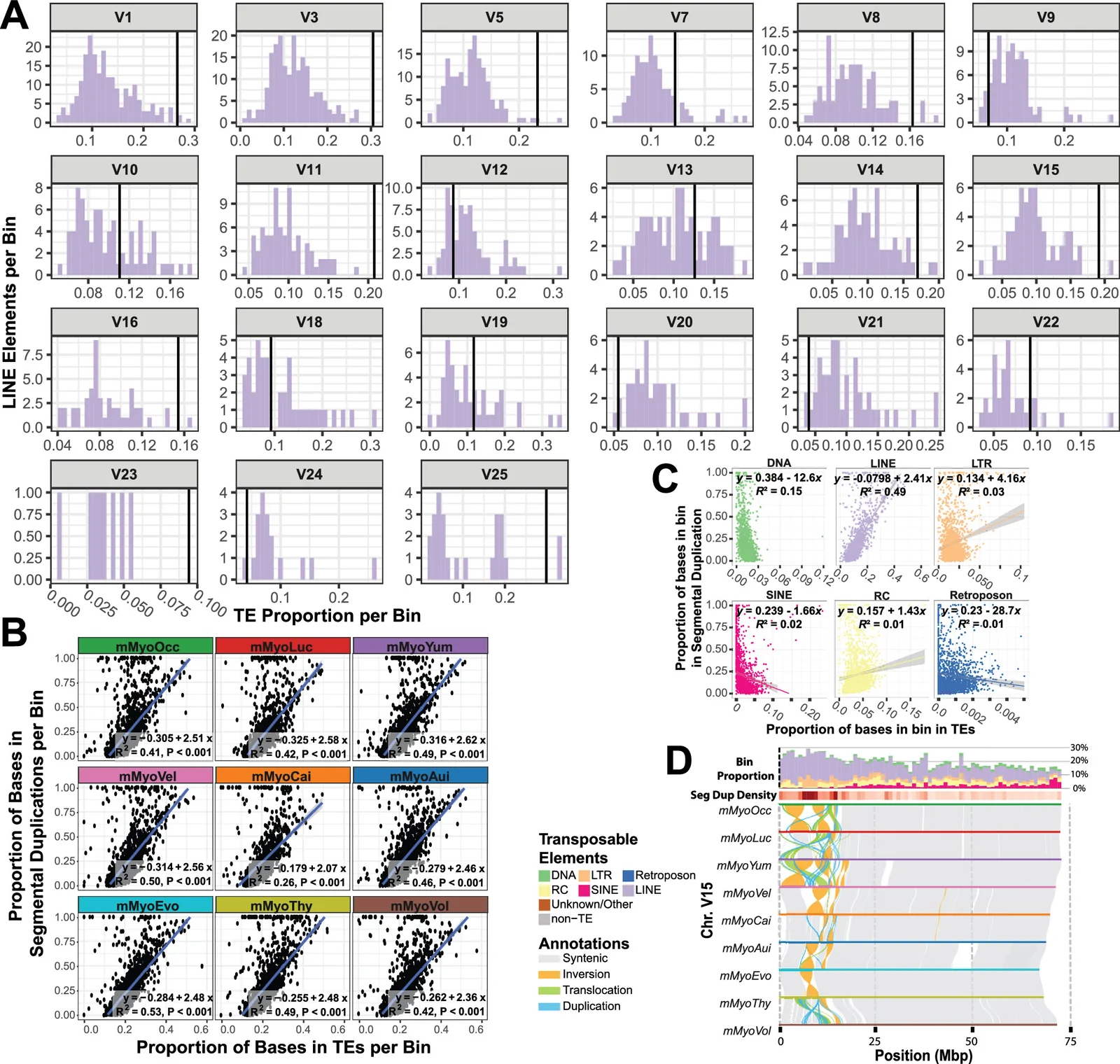
Correlations of TEs and structural variation in *Myotis* genomes. **A**) Histogram of LINE element counts in 1 Mb bins across chromosomes in *M. velifer*. Black lines indicate the estimated location of the centromeric region as a 20 Mb bin. **B**) Correlation between the proportion of bases within segmental duplications and the proportion of bases within TEs in bins across 9 Nearctic *Myotis* genomes. The blue line represents the linear regression of the correlation between the two proportions, and the R^2^, p-value, and equation for the correlations are plotted in each graph. **C**) Correlation between the proportion of bases within segmental duplications and the proportion of bases within TEs in bins for *M. velifer* per TE family**. D**) Synteny between chromosome V15 across 9 *Myotis* species. The binned distribution of transposable elements (top) and segmental duplications (red heatmap, bottom) in *M. velifer* are shown above the syntenic alignment in phylogenetic order. The ~20-Mb block at the subtelomeric end of Chr V15 spans several immune-related and interleukin signalling genes, including IL-1 and IL-36.

We investigated the landscape of structural variation within the tightly conserved *Myotis* karyotype. With only 6 exceptions across over 60 studied species, all *Myotis* have a conserved 2*n* = 44 karyotype—a remarkable phenomenon for a genus spread across six continents and 33 million years of divergence^4,6^. The *Myotis* karyotype comprises three large autosomes; one small metacentric autosome; 17 small telocentric autosomes; and metacentric X and Y chromosomes^16^. Consistent with this broad cytological conservation, we find large scale synteny across the Nearctic *Myotis* in this study (Fig. 1e). However, structural variants (SVs), including inversions, duplications and translocations, are relatively common within chromosomes, especially adjacent to putative centromeric regions (Fig. 1e,f). This includes an approximately 20 Mb block at the subtelomeric end of chromosome V15 that displays frequent and recurrent inversions and translocations across the Nearctic *Myotis*, and contains a 10 Mb locus that was recently identified as a potential target of recent selection by adaptive introgression^18^ (Fig. 1e (red bar) and Extended Data Fig. 3d).

Using SyRI^19^, we identified between 6,813 and 8,013 SVs per Nearctic *Myotis* genome relative to their outgroup, *M. myotis*; 97–99% of these events were under 10 kb. In the three large autosomes, which constitute around 30% of the genome, we catalogued an average of 509 SVs (Supplementary Table 3). By contrast, in the small autosomes, constituting around 65% of the genome, we observed an average of 316 events, highlighting the distinct structural evolution between these chromosome types with SVs found at twofold higher density in larger autosomes (Supplementary Table 3). However, large (at least 10 kb) duplications, inverted duplications and inverted translocations were more common on small autosomes compared with on the large autosomes (Supplementary Table 3). We also quantified the distribution of transposable elements (TEs) across chromosomes (Fig. 1f,g). Consistent with previous observations in other Chiropteran genomes^20^, long interspersed nuclear elements appeared to be enriched around the predicted centromere (Fig. 1f), with simulations showing that this trend is primarily limited to the long, metacentric autosomes (Extended Data Fig. 3a and Supplementary Table 3). The concentration of segmental duplications was also significantly correlated with TE density in each species (linear regression, *P* < 0.001; Fig. 1f,h and Extended Data Fig. 3b), highlighting the importance of TEs in facilitating structural evolution. Together, these results highlight high levels of structural variation occurring in the context of a highly constrained karyotype.

## A trans-species *PKR* copy-number variant

**Fig. 2:**
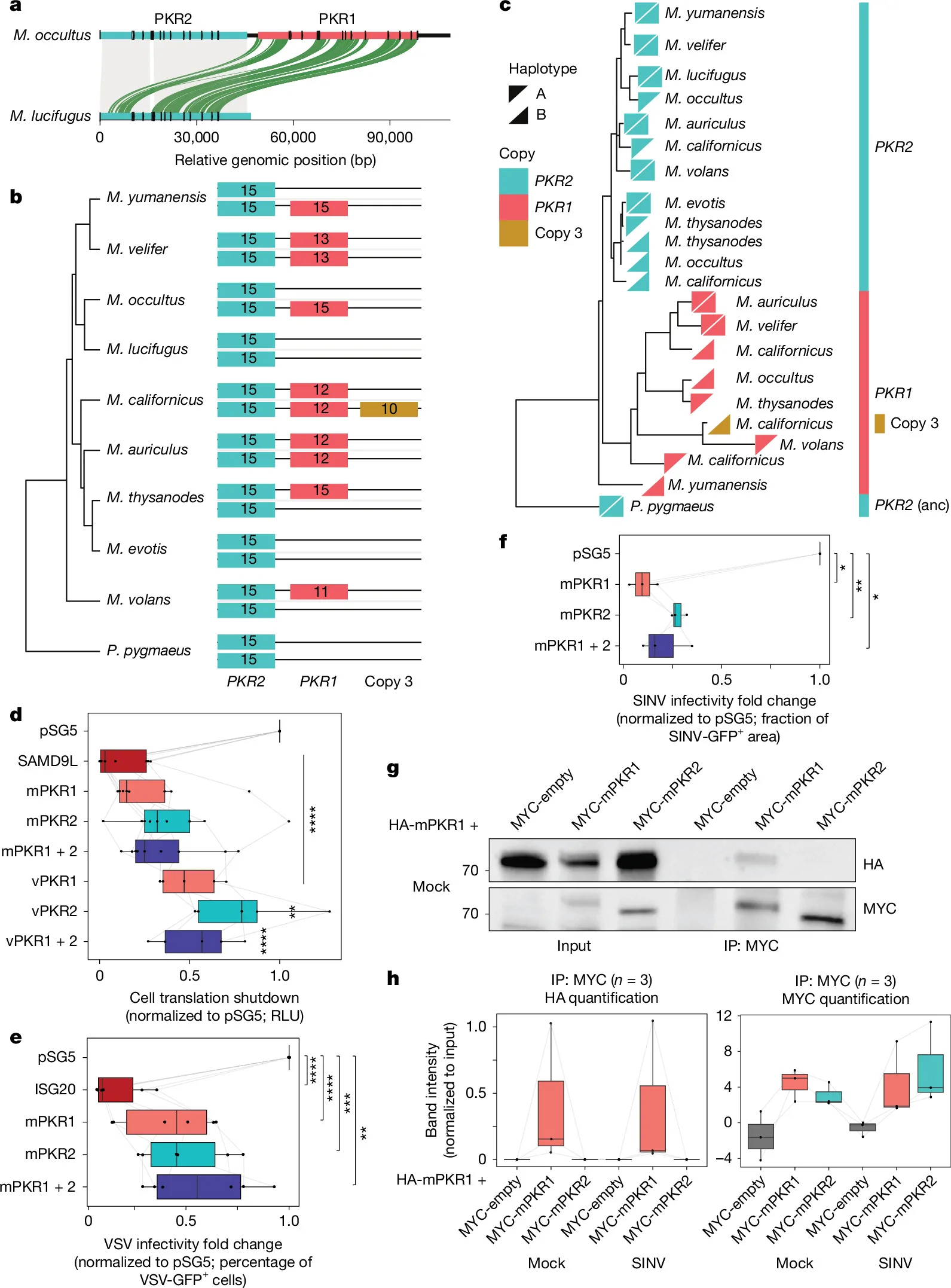
Evolutionary history and function of an actively segregating copy-number polymorphism of *PKR* in *Myotis.* **a**, The structural comparison of PKR haplotypes in two species. Orthologous regions are indicated by grey bands, syntenic duplications are indicated in green and exons are indicated by black marks. **b**, Cartoon of the *PKR* locus in the two phased haplotype assemblies of each *Myotis* species in this study, with the number of exons per copy. While *PKR2* is present across all haplotypes, *PKR1* and *PKR* copy 3 are polymorphic. **c**, Reconciled gene tree for *PKR*s across all haplotypes and species shown in **b**. Haplotype corresponding to the reference (**a**) and alternative (**b**) haplotype for each species are represented by upper- and lower-diagonal triangles, respectively. anc, ancestral.** d**, The effect of *Myotis* *PKR* expression on luciferase reporter translation in *PKR*-KO cells, measured in relative light units (RLU) and normalized to the empty pSG5 control. Human SAMD9L-GoF is a positive control for translation inhibition^69^. **e**,**f**, The effect of PKR on viral infection by VSV-GFP (**e**; Extended Data Fig. 4e) and SINV-GFP (**f**; 34 h after infection, Extended Data Fig. 4c), measured by flow cytometry as the percentage of GFP^+^ cells and by live imaging as the GFP^+ ^area normalized to total cellular area, respectively, normalized to the pSG5 control. Although all of the conditions restricted VSV and SINV, expression of both PKR1 + PKR2 was not beneficial against these viral infections. ISG20 was used as a positive control of VSV-GFP restriction^70^. **g**, Co-immunoprecipitation (co-IP) analysis of *PKR*-KO cells transfected with *Myotis* HA-PKR1 and *Myotis* MYC-PKR1, *Myotis* MYC-PKR2 or MYC-empty vector control. Proteins were pulled-down with anti-MYC beads and lysates from 5% input or IP samples were run on a western blot and stained for HA and MYC (SINV conditions are shown in Extended Data Fig. 4c). **h**, Quantification of the three independent experiments (mock and SINV) for HA (left) and MYC (right). Data are mean ± s.e.m. of at least three independent experiments. Statistical significance was assessed using unpaired *t*-tests; *P < 0.05, **P < 0.01, ***P < 0.001, ****P < 0.0001. mPKR, *M. myotis* PKR; vPKR, *M. velifer* PKR. Gel source data are provided in Supplementary Fig. 1.

Among the SVs identified in Nearctic *Myotis*, the gene *PKR*—previously shown to be duplicated in certain *Myotis*^21^—stood out as an interferon-stimulated gene with antiviral activity against both DNA and RNA viruses. Using our near-complete genome assemblies, we fully resolved the sequence and structure of the two previously known haplotypes: H1, containing a single copy of *PKR* (*PKR2*); and H2, containing two tandemly duplicated copies of *PKR* (*PKR1* and *PKR2*; Fig. 2a). We also identified a third haplotype, H3, with three tandem duplicates of *PKR* (*PKR1*, *PKR2* and a third copy) present only in *Myotis* *californicus*. Notably, while 7 out of 9 *Myotis* species carried duplicated haplotypes, 5 of these cases were heterozygous for the duplicated haplotype (that is, H1/H2 or H2/H3; Fig. 2b). Two *Myotis* individuals (*M. lucifugus* and *Myotis* *evotis*) carried only non-duplicated haplotypes (that is, H1/H1; Fig. 2b). To determine the evolutionary history of the duplicates, we used GeneRax^22^ to construct a tree from alignments of all *PKR* gene copies across Nearctic *Myotis*, using *Pipistrellus pygmaeus* as a non-*Myotis* outgroup (Fig. 2c). We found that *PKR2* is the ancestral copy of *PKR*, and that *PKR1* originated from a single duplication event at the root of *Myotis*. These results highlight that both the duplicated and unduplicated haplotypes have probably been segregating for tens of millions of years, representing an ancient trans-species polymorphism.

PKR is a stress response and innate immune factor that becomes active after detection of double-stranded RNA through sensing, autophosphorylation and dimerization^23^. Active PKR shuts down protein translation and restricts viral replication^23^. Although PKR1 and PKR2 are both expressed in basal or interferon-stimulated *Myotis velifer* cells, only their independent functional impacts have previously been investigated^21^. Given the co-expression and the complex haplotype diversity that we identified in *Myotis*, we set out to determine whether the two *PKR* copies act additively, synergistically or as dominant negatives in several essential functions. We investigated the functional impact of the duplicates’ co-expression on total PKR protein expression levels, cell viability, inhibition of translation, antiviral restriction and homodimer/heterodimer formation (Fig. 2d–h). To pinpoint the contribution of PKRs, we used a heterologous cell system^21^ with *PKR*-KO HeLa cells transfected with either an empty pSG5 plasmid (control), or pSG5 encoding PKR1, PKR2 or PKR1 + PKR2 (1:1 ratio) from *Myotis myotis* or *M. velifer*. In this system, PKR transfection leads to its autoactivation^21^.

**Extended Data Fig. 4:**
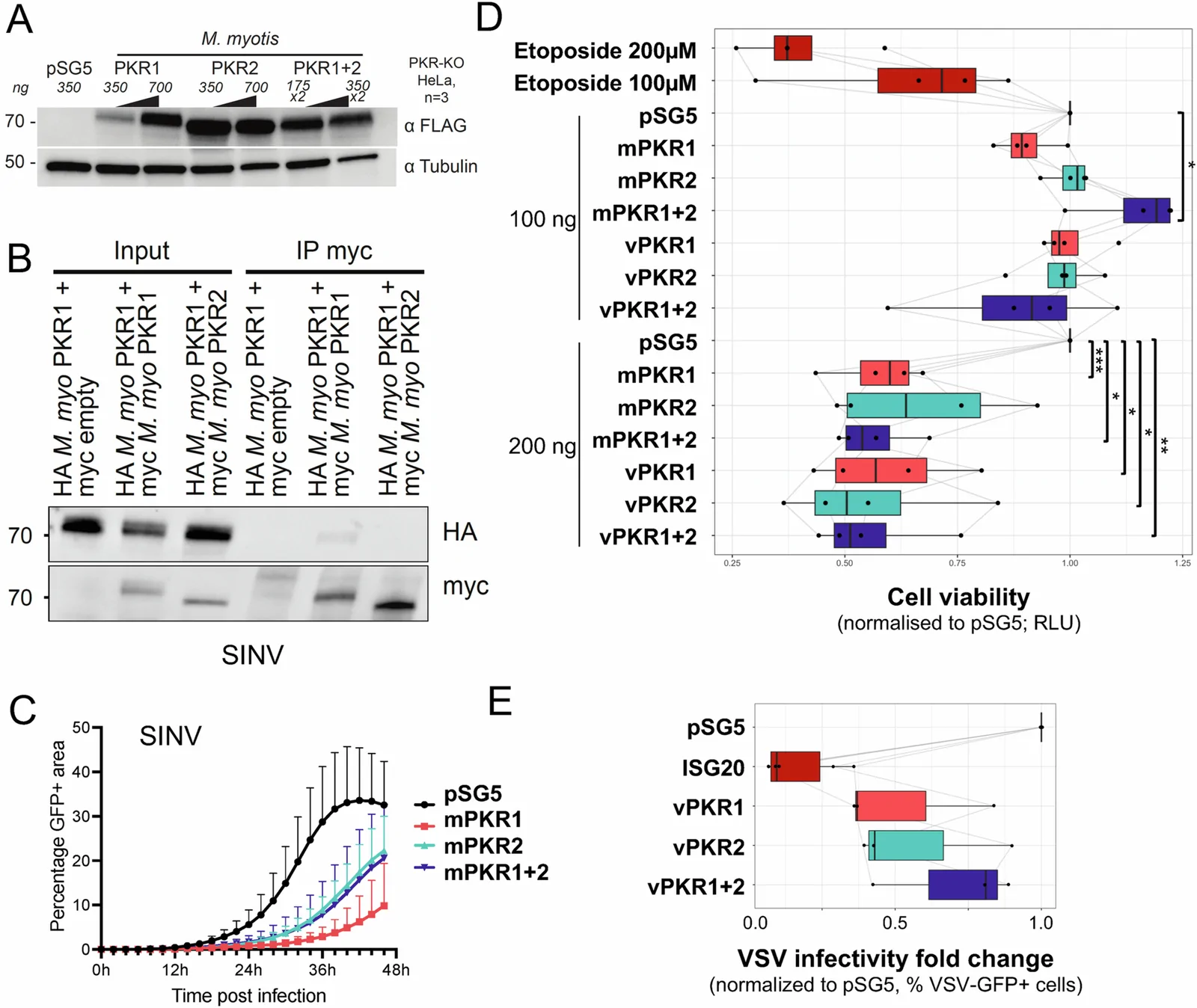
Additional functional characterization of *Myotis* PKR duplicates. **A**) Steady-state protein expression levels in PKR-KO HeLa cells expressing FLAG-tagged *Myotis myotis* PKR1 and/or PKR2. Western blots targeting FLAG or Tubulin (loading control) in lysate of cells transfected with either empty vector (pSG5), PKR1-FLAG, PKR2-FLAG, or an equimass mix of both PKR vectors (total 350 ng or 700 ng). **B**) Co-immunoprecipitation (coIP) of PKR-KO HeLa cells transfected with plasmids encoding HA-tagged *Myotis myotis* PKR1 and either myc-tagged *Myotis myotis* PKR1, myc-tagged *Myotis myotis* PKR2 or a myc empty vector control in SINV-GFP infected conditions. Proteins were pulled down with anti-myc coated beads and lysates from 5% input or immunoprecipitated samples were run on a western blot and stained for HA and myc. Image representative of 3 independent experiments. **C**) PKR-KO HeLa cells transfected with plasmids encoding an empty vector control, *Myotis myotis* PKR1, PKR2 or both PKR1 and PKR2 were infected with SINV-GFP at MOI 0.2 and GFP fluorescence was monitored over time by live imaging. Mean +/− standard deviation of the percentage of GFP fluorescent area over total cell area was plotted for 3 independent experiments, each representing the average value of six photos from two duplicate wells. **D**) Effect of PKRs on cell viability, normalized to the control. Etoposide treatments are positive controls of cell death. Only high transfection of PKRs impacted cell viability (200 ng total of DNA for 10,000 cells). mPKR, *Myotis myotis* PKRs; vPKR, *Myotis velifer* PKRs. Error bars indicate mean ± SEM for at least three independent experiments. Statistical significance was assessed using an unpaired t-test (*, p < 0.05; **, p < 0.01; ***, p < 0.001; ****, p < 0.0001). **E**) Effect of *Myotis velifer* PKRs on viral infection by VSV-GFP, measured by flow cytometry as the % of GFP+ cells, normalized to the pSG5 control. ISG20 served as a positive control of VSV-GFP restriction^70^. For gel source data, see Supplementary Fig. 1.

First, we found that, although *Myotis* Flag–PKR1 was expressed at lower levels than Flag–PKR2, PKR1 + PKR2 co-expression did not affect the overall steady-state protein expression levels of PKRs (Extended Data Fig. 4a). Second, we performed cell viability assays at two doses of transfected PKRs and found that cell viability was only affected at a high dose (Extended Data Fig. 4d). Third, using a luciferase reporter translational assay, we found that PKR1 + PKR2 coexpression led to an intermediate translation shutdown, showing neither synergistic nor dominant negative effect of the PKR duplicates (Fig. 2d). Fourth, we tested PKR1 + PKR2 co-expression effects on infections by two different model RNA viruses: VSV-GFP (vesicular stomatitis virus encoding a GFP reporter, a representative of *Rhabdoviridae* family that is common in bats, including *Myotis*) and SINV-GFP (Sindbis virus, a representative of *Togaviridae*) (Fig. 2e,f and Extended Data Fig. 4c,e). We found that PKR1 + PKR2 restricted viral infections to a similar extent—or slightly lower in the case of VSV—to PKR1 or PKR2 alone (Fig. 2e,f and Extended Data Fig. 4c,e; *P* > 0.05). While PKR1 homodimerized, PKR1 and PKR2 did not form heterodimers (Fig. 2g,h and Extended Data Fig. 4b in the SINV condition). This suggests that, since their duplication, divergence between *Myotis* PKR copies has been particularly strong at the protein–protein interface hindering their ability to form heterodimers.

These results suggest that PKR1 and PKR2 do not exhibit dominant-negative or synergistic effects but, instead, act additively in their primary cellular effector functions. While their duplication may broaden resistance to certain DNA and RNA viral antagonists^21^, the increased cell toxicity observed at high PKR doses suggests a trade-off that could explain the absence of PKR duplication in other mammals. This trade-off may also account for the persistence of both duplicated and non-duplicated haplotypes in *Myotis*. Consistent with this, PKR1, which is generally more potent in our experiments, is expressed at significantly lower levels than PKR2 in *M. velifer* cells^21^. Together, these results reveal gene duplicates still segregating across several *Myotis* species, probably shaped by a balance between antiviral defence and cell toxicity.

## Modes of adaptation to DNA and RNA viruses

**Fig. 3:**
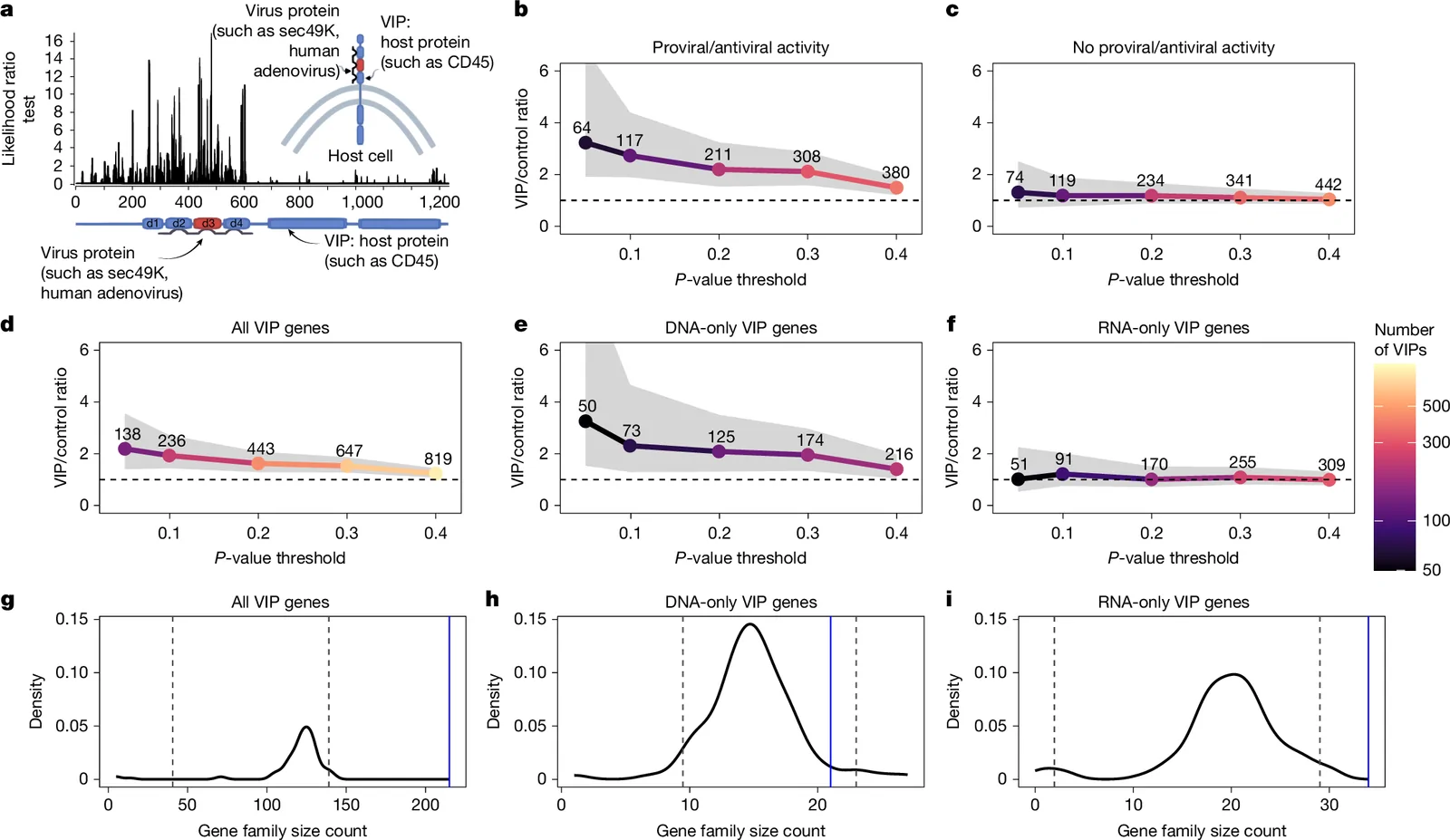
Adaptation to DNA viruses, but not RNA viruses, is enriched in *Myotis* and other bats. **a**, Diagram of an example VIP, the host cell transmembrane receptor CD45, showing the strength of adaptation at each codon and the locations of direct interactions with the human adenovirus protein sec49K. **b**–**f**, The ratio of positive selection of VIP genes associated with proviral/antiviral activity (**b**) or no proviral/antiviral activity (**c**), all VIP genes (**d**), DNA-only VIP genes (**e**) and RNA-only VIP genes (**f**) in only *Myotis* bats in VIPs versus matched sets of control genes at different *P* value thresholds. The solid line shows the median ratio; the colour of the line and the number above each point represent the number of VIPs with significant BUSTED-MH *P* values at the given threshold, and therefore used with the same number of matched control genes for each ratio calculation; the grey band represents the 95% confidence interval generated by bootstrapping sets of matched control genes. The 95% confidence interval widens at lower *P* values because there are fewer VIPs with significant *P* values as the *α* threshold decreases, reducing power. **g**–**i**, The number of VIP genes (all VIP genes (**g**), DNA-only VIP genes (**h**) and RNA-only VIP genes (**i**)) inferred by CAFE to have significantly expanded or contracted in copy number on at least one branch of the Nearctic *Myotis* phylogeny (blue line) compared with the distribution across 100 randomized sets of non-VIP control genes (black line). The vertical dashed lines indicate the 95% highest density interval for the control gene distribution.

To further examine how viruses may have shaped the genomes of *Myotis* bats, we tested for adaptive signatures in virus-interacting proteins (VIPs) in *Myotis* and other bats. VIPs are host proteins that physically interact with viral proteins (such as CD45; Fig. 3a), and can be proviral (contributing to viral infection; for example, viral receptors), antiviral (protective against viral infection; such as interferons) or both, depending on infection stage and virus type. VIPs commonly have major roles in basic host function and, as a result, most host cell biological pathways include VIPs. Previous studies investigating positive selection across mammals have found an enrichment for adaptation among a set of 5,527 manually curated VIPs, defined as host proteins that have at least one experimentally verified physical interaction with a viral protein, RNA or DNA^24^.

**Extended Data Fig. 5:**
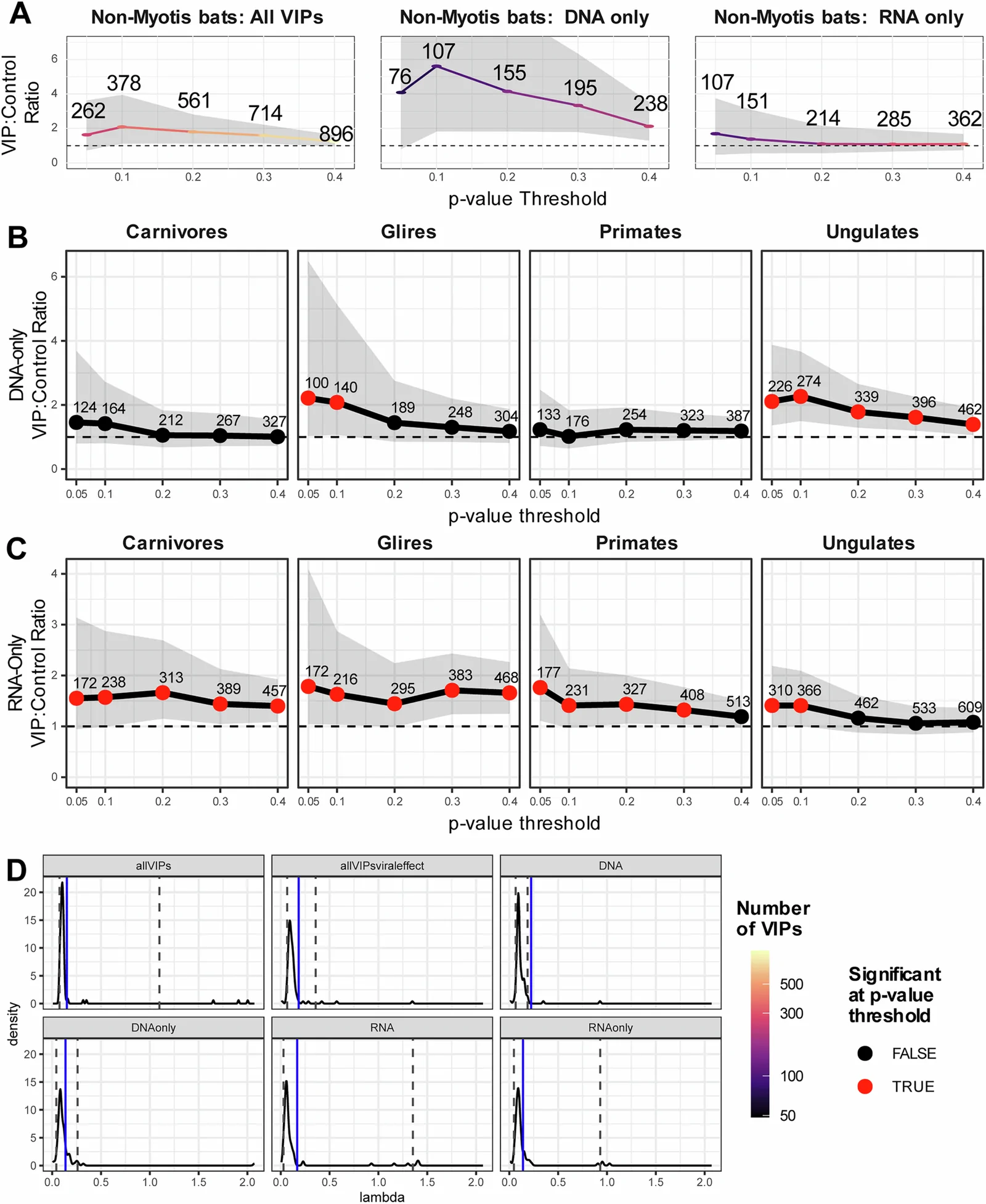
VIP selection enrichments in bats and other mammals. **A**) Enrichment for selection of all VIPs (left), DNA-only VIPs (middle), and RNA-only VIPs (right) versus matched control genes in non-*Myotis* bats. **B**) Enrichment for selection of DNA-only VIPs versus matched control genes in 4 non-bat clades of mammals. **C**) Enrichment for selection of RNA-only VIPs versus matched control genes in 4 non-bat clades of mammals. For **A-C**, plots show the ratio of selection (**ω**) p-values of viral interacting proteins (VIPs) v.s. matched controls for VIPs and control gene sets identified as under positive selection by BUSTED-MH as a function of p-value threshold. **D**) CAFE p-value estimates for the birth-death rate (λ) of VIP genes and VIP-subsets compared to randomized matched sets of non-VIP genes. The 95% confidence interval of the distribution of the bootstrapped non-target control genes is indicated by the dotted lines around each distribution; the p-value estimated for the target gene sets are indicated by the solid blue line.

By calculating an enrichment score from the ratio of positive selection in VIPs compared with their matched control genes using BUSTED-MH^25^, we found that like other mammals, *Myotis* show an enrichment for adaptation at VIPs (Fig. 3b–d and Supplementary Table 5). Physical host–virus interactions may not always result in fitness effects in the host. We therefore repeated our analysis using a gene set restricted to VIPs with experimental evidence of specific pro- or anti-viral effects, and therefore with a stronger expectation of fitness effects. We expect that, if viruses were not the primary driver of adaptation in VIPs, then there would be no difference in the enrichment of selection in VIPs with versus without demonstrated pro- or anti-viral effects. However, we observed an even stronger significant elevation in the ratio of positive selection in these proviral and antiviral VIPs (Fig. 3b and Supplementary Table 5), but no elevation in this ratio in VIPs without experimental evidence of specific pro- or anti-viral effects (Fig. 3c and Supplementary Table 5). This is consistent with the expectation of viral interaction as the cause of enrichment of positive selection in VIPs in bats^26^. We repeated this analysis using a dataset of 47 publicly available non-*Myotis* bat genomes, and confirmed these same patterns across bats more broadly, even when excluding *Myotis* genomes (Extended Data Fig. 5a).

Previous work has suggested that bats may have different physiological responses to DNA and RNA viruses^27^. To determine whether this was reflected in genomic VIP adaptation, we compared the enrichment of positive selection in VIPs that interact only with DNA viruses (DNA-only VIPs) with those that interact only with RNA viruses (RNA-only VIPs). Notably, we found that VIP adaptation in *Myotis* and other bats is driven by selection only in DNA VIPs (Fig. 3e and Extended Data Fig. 5b) in contrast to the observed pattern in RNA VIPs, which show no evidence of genome-wide enrichment in adaptation (Fig. 3f and Extended Data Fig. 5c).

Adaptation to viral pathogens can also occur through gene copy-number changes^28–30^. We tested whether VIPs were enriched among genes that were recently gained or lost throughout *Myotis* using a method described previously^31^ to test the cumulative per-gene, per-branch copy-number changes and birth–death rates of VIPs compared with other non-VIP genes. While the birth–death rates of VIP and non-VIP genes were comparable (*P* = 0.071; Extended Data Fig. 5d), VIP genes were significantly more likely to have undergone expansions and/or contractions on at least one branch of the *Myotis* family (*P* < 0.001; Fig. 3g). Furthermore, we find that this pattern is driven exclusively by copy-number changes in RNA-only VIPs, and not by changes in copy number of DNA-only VIPs (Fig. 3h,i). This suggests that, relative to the general variation in gene family birth–death rates across species, RNA VIPs are more dynamic across the Nearctic *Myotis* as a whole.

In contrast to what we observe in bats, VIP adaptation in humans is driven by positive selection in RNA—and not DNA—VIPs^32^. To investigate whether DNA VIP-driven adaptation in bats is exceptional among mammals, we replicated these analyses across four other large mammalian clades that are well represented among publicly available mammalian genomes: primates, Glires, Euungulata and Carnivora. We found that, while other mammalian orders show a mix of adaptation enrichments in both RNA and DNA VIPs, none show the absence of genome-wide enrichment of protein-coding adaptation in RNA VIPs observed in bats (Extended Data Fig. 5a–c). These results highlight that bats, including *Myotis*, exhibit unique modes of adaptation to DNA viruses compared to RNA viruses, in contrast to all other mammals.

## Body size and lifespan evolution in bats

Beyond their immune adaptations, bats are exceptional as the longest-lived clade of mammals after correcting for their body size^1^, demonstrating an approximately 11× range of lifespans within an approximately 650× range of body sizes^8,33^. *Myotis* species in particular exhibit the full dynamic range of bat lifespans within a narrow range of body sizes. While bats have been noted as an exception to the strong allometric scaling (positive correlation with body size) of lifespan otherwise seen across mammals and other metazoans, this exception has not been tested using phylogenetically corrected statistics leveraging well-resolved phylogenies.

**Fig. 4:**
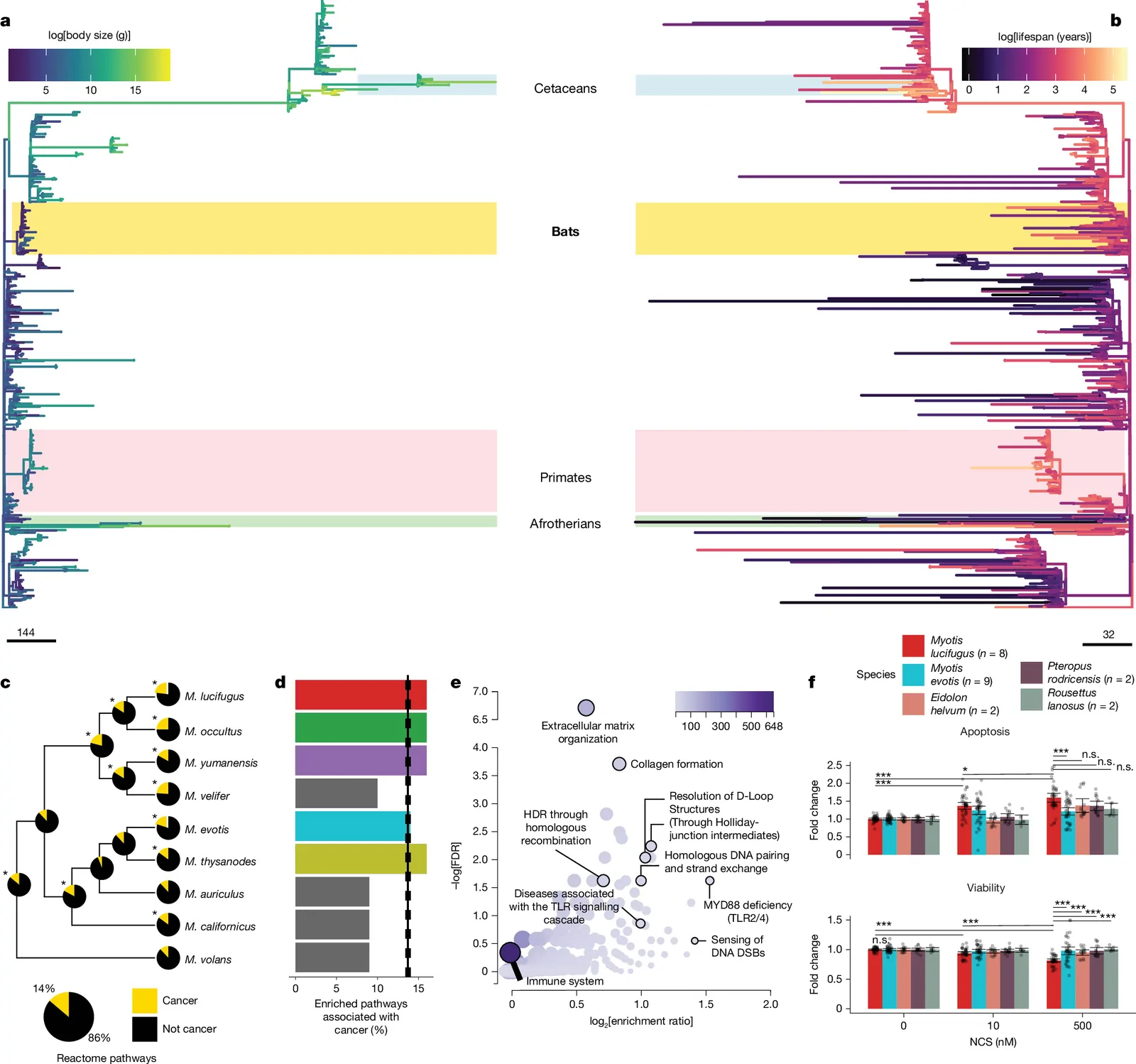
Evolution of cancer risk and resistance in *Myotis* and other mammals. **a**,**b**, Cophylo plot of the evolution of body size (**a**) and lifespan (**b**) across *Eutheria*. The branch lengths in **a** and **b** are scaled proportional to the rate of change of the trait over time. **c**, The phylogeny of Nearctic *Myotis* species. Inset: the proportion of the top 100 Reactome pathways over-represented among genes under selection at each node that are associated with cancer-related processes. Bottom, the expected proportion of pathways are cancer associated after 1,000 random samples of 100 pathways from the full Reactome database. The asterisks represent nodes with proportions greater than the expected value at *P* ≤ 0.05 based on the Fisher’s exact test. **d**, The proportion of the top 100 Reactome pathways over-represented among genes under selection across all nodes in a species’ evolutionary history that are associated with cancer-related processes.** e**, Over-represented pathways in Reactome among the union set of genes under selection across all nodes in the evolutionary history for *M. lucifugus*. **f**, The viability and apoptosis fold change in primary skin fibroblasts of five bat species in response to different doses of NCS, a potent inducer of DNA double-strand breaks. The points represent individual replicates normalized to each species’ control; the bars represent mean ± 95% confidence intervals. NS, not significant (*P* > 0.05).

**Extended Data Fig. 6:**
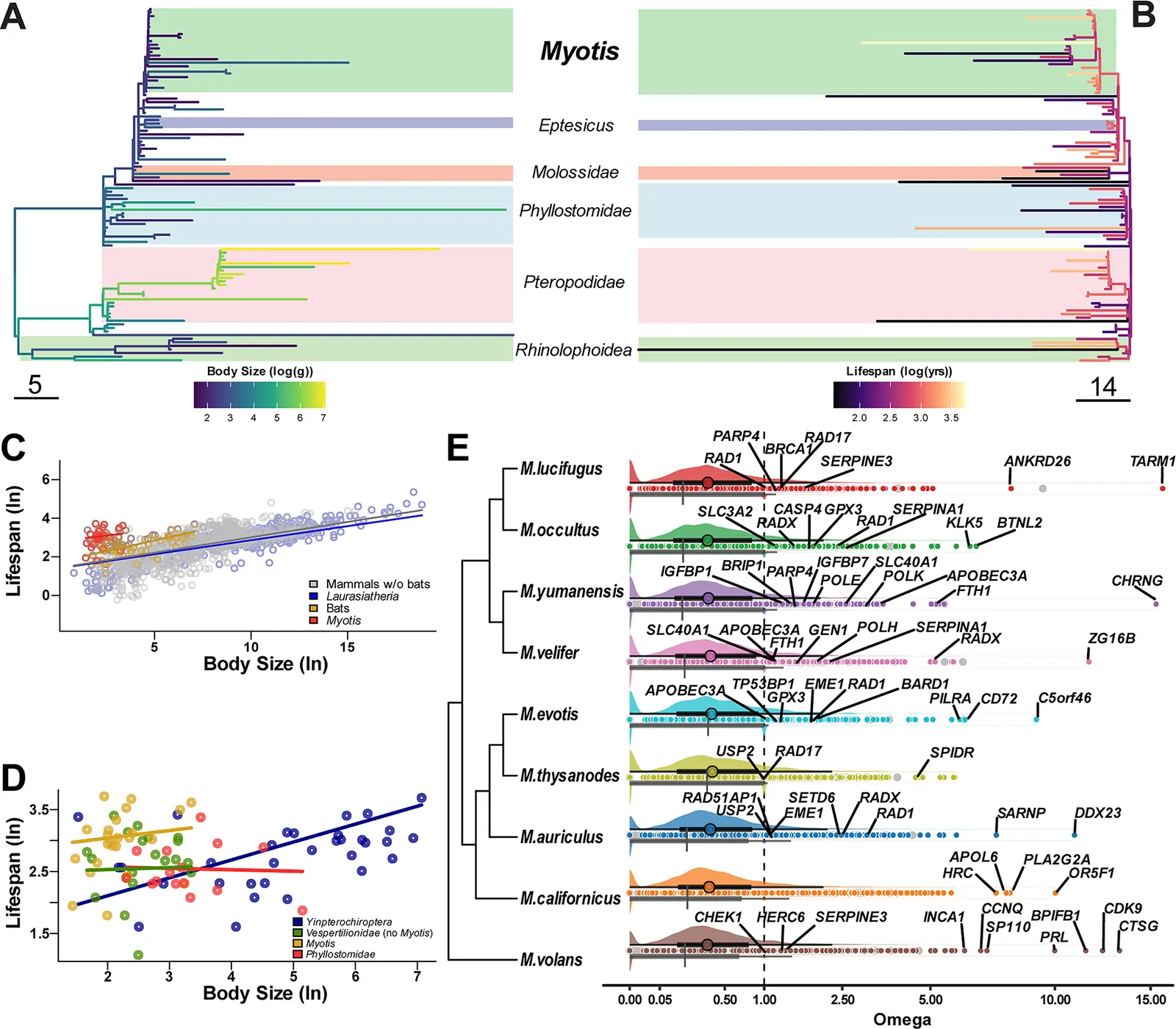
Evolution of body size and lifespan across *Myotis.* **A-B**) Estimates of body size (**A**), and lifespan (**B**) in Chiroptera with major bat families highlighted. **C-D**) Phylogenetic least-squares regression for lifespan (log(yrs)) as a function of body size (log(kg)) across mammals (**C**) and bats (**D**) in the Upham et al.^34^ phylogeny; lines represent the phylogenetically-corrected generalized least squares regression for each group indicated in the legend. **E**) Raincloud plots of genes under selection (p_adj_ ≤ 0.05) by aBSREL in each terminal species. Each histogram represents the distribution of the highest omega (**ω**) value for each gene after multiple testing correction (p_adj_ ≤ 0.05). The 95% confidence interval and median for significant **ω**’s are represented by the black bar and circle, respectively; the 95% confidence interval and median **ω** for all genes are shown in grey below. Individual genes’ **ω**’s are represented by coloured points. Genes that are associated with pathways previously explored in the literature for bats, such as insulin signalling, iron metabolism, DNA damage response, and SERPIN-family genes, are highlighted.

To test the hypothesis of non-allometric scaling of lifespan in bats, we modelled the evolution of body size and lifespan independently across a supertree of over 1,000 mammals^34^ (Fig. 4a,b, Extended Data Fig. 6a,b and Supplementary Table 6). We generally observed agreement between evolutionary patterns of body size and lifespan, such as in whales (Cetacea)^35^, elephantids (Proboscidea)^36^ and primates^37^ (Fig. 4a,b). However, while only minor changes were observed in their body size, we observed some of the largest and most-rapid changes in lifespan across mammals in bats (Fig. 4a,b and Extended Data Fig. 6a,b and Supplementary Table 6). These changes are largely independent between species and genera, consistent with the theory of multiple independent increases in lifespan across bats. We used phylogenetically corrected generalized linear models and analysis of covariance to quantify the relationship between body size and lifespan across mammals. We find that bats experience a 40% greater increase in lifespan per 1% increase in body size compared with non-bat mammals (0.223% increase in lifespan versus 0.159% increase in lifespan per 1% increase in mass); however, these rates were not significantly different after phylogenetic correction (Extended Data Fig. 6c,d; phylogenetic analysis of covariance, *P* = 0.29). In *Myotis*, we saw many of the fastest increases in lifespan relative to their most recent ancestor, as measured by the change in their lifespan over the divergence time from their most recent ancestor (Δlifespan): *M. brandtii* (8.6 Δlifespan, 99th percentile), *M. lucifugus* (8.4 Δlifespan, 99th percentile), *M. myotis* (2.5 Δlifespan, 93nd percentile), *Myotis grisescens* (1.3 Δlifespan, 92nd percentile) and the *Myotis* common ancestor (1.7 Δlifespan, 89th percentile) (Fig. 4b, Extended Data Fig. 6b and Supplementary Table 6). Together, these results demonstrate that *Myotis* bats and their recent ancestors have evolved some of the most extreme increases in lifespan among mammals, despite similar allometric lifespan scaling of bats and mammals after phylogenetic correction.

## Pleiotropic immune and DNA damage response

Rapid changes in body size and lifespan can have major implications for the evolution of cancer risk and resistance across mammals^38^. While lifetime cancer risk scales proportionally to both body size and lifespan within species^38,39^, there is little to no correlation between body size, lifespan and cancer risk across species^39,40^. This observation, known as Peto’s paradox^40^, suggests that species with more cells or longer lifespans have adapted to reduce their cancer risks.

We hypothesized that the extreme changes in lifespan observed throughout the *Myotis* phylogeny should exert a selective pressure on genes associated with cancer processes. Using aBSREL^41^ to test for selection at terminal and internal branches within *Myotis*, we found that, per node, an average of 5.46% and 20.8% of protein-coding genes had at least one region under positive or negative selection, respectively (Supplementary Table 7). Genes under either positive or negative selection were enriched for several pathways in immunity, cancer and ageing, with many intersecting multiple of these processes, suggesting possible pleiotropic selective pressures (Supplementary Table 7).

We quantified the proportion of cancer-associated pathways^42^ over-represented among genes under positive selection throughout the phylogeny (Fig. 4c,d). Among genes under positive selection, we observed that most nodes within Nearctic *Myotis* were enriched for cancer hallmark pathways, especially at the recent ancestors of the longest-lived species (for example, *M. lucifugus* + *M. occultus*; Fig. 4c). Furthermore, when considering the union of all genes under positive selection in the evolutionary history of each species since their common ancestor, we observed significant enrichments in the representation of cancer-associated pathways in many of the lineages with the greatest cumulative increases in lifespan (*M. lucifugus*, *M. occultus, M. evotis* and *M. yumanensis;* Fig. 4d and Extended Data Fig. 6e).

The longest-lived bat in our study, *M. lucifugus*, had an over-representation of pathways specifically associated with DNA double-stranded break repair when looking at both lineage-wide and node-specific enrichments in positive selection using the Reactome database^43^ (Fig. 4e and Supplementary Table 7). This includes 35 out of 65 genes in the high-fidelity ‘Homologous Recombination Repair’ Reactome pathway, and 21 out of 37 members of the ‘Homology-Directed Repair through Single-Strand Annealing’ Reactome pathway (Fig. 4e, Extended Data Fig. 6e and Supplementary Table 7). These results suggested that *M. lucifugus* might have an enhanced response to DNA double-stranded breaks relative to other bats. To test this hypothesis, we assessed the tolerance of *M. lucifugus* primary skin fibroblasts to neocarzinostatin (NCS), a potent radiomimetic agent that induces DNA double-stranded breaks^44^ (Fig. 4f and Supplementary Table 8), compared with primary skin fibroblasts derived from *M. evotis* and three non-*Myotis* bats (*Eidolon helvum,* *Pteropus rodricensis* and *Rousettus lanosus*). At low doses of NCS, *M. lucifugus* was the only species with sensitivity to NCS after 24 h, with a drop in viability and concomitant increase in apoptosis. At high doses, *M. lucifugus* had the highest level of apoptosis and the greatest drop in viability of all the bats tested. This is consistent with other long-lived species, including elephants^45,46^, naked mole rats^47^ and bowhead whales^48^, in which longevity is associated with an increased ability to clear out damaged cells.

**Fig. 5:**
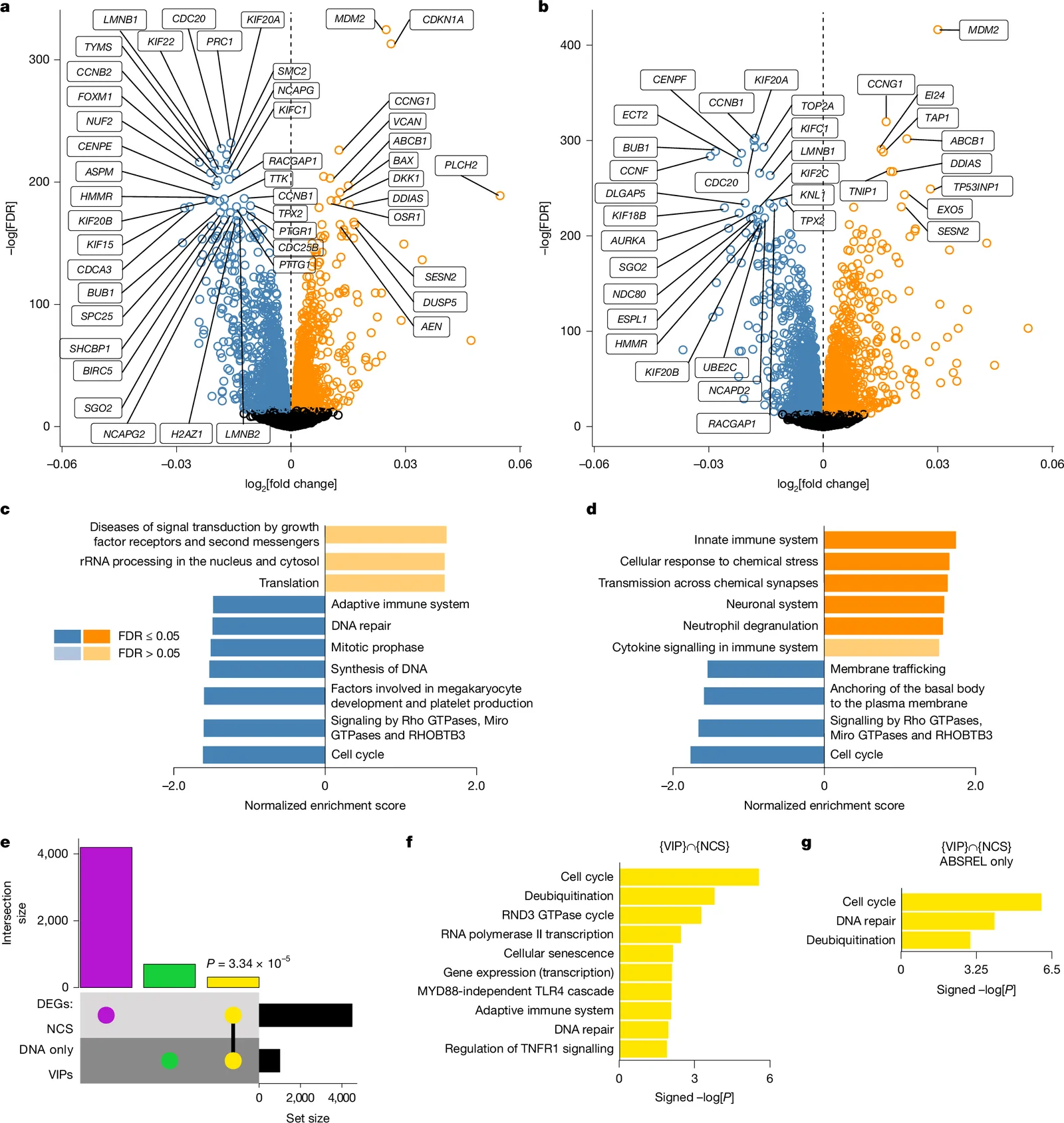
Pleiotropy among genes under selection in *M. lucifugus* between DNA-damage-responsive genes and DNA-only VIPs. **a**,**b**, Genes that are differentially expressed in *M. lucifugus* primary skin fibroblasts after 6 h (**a**) and 18 h (**b**) of treatment with 100 nM NCS. **c**,**d**, Pathway-level gene set enrichment analysis (GSEA) of genes differentially expressed at 6 h (**c**) and 18 h (**d**) after treatment. **e**, Upset plot between genes that are differentially expressed after NCS treatment and DNA-only VIPs; the hypergeometric *P* value of the intersection is shown. **f**, Pathway-level over-representation analysis of genes that are DNA-only VIPs and are also differentially expressed in response to NCS treatment. **g**, Pathway-level over-representation analysis of genes identified as under selection in *M. lucifugus* using aBSREL that are DNA-only VIPs and are also differentially expressed in response to NCS treatment.

**Extended Data Fig. 7:**
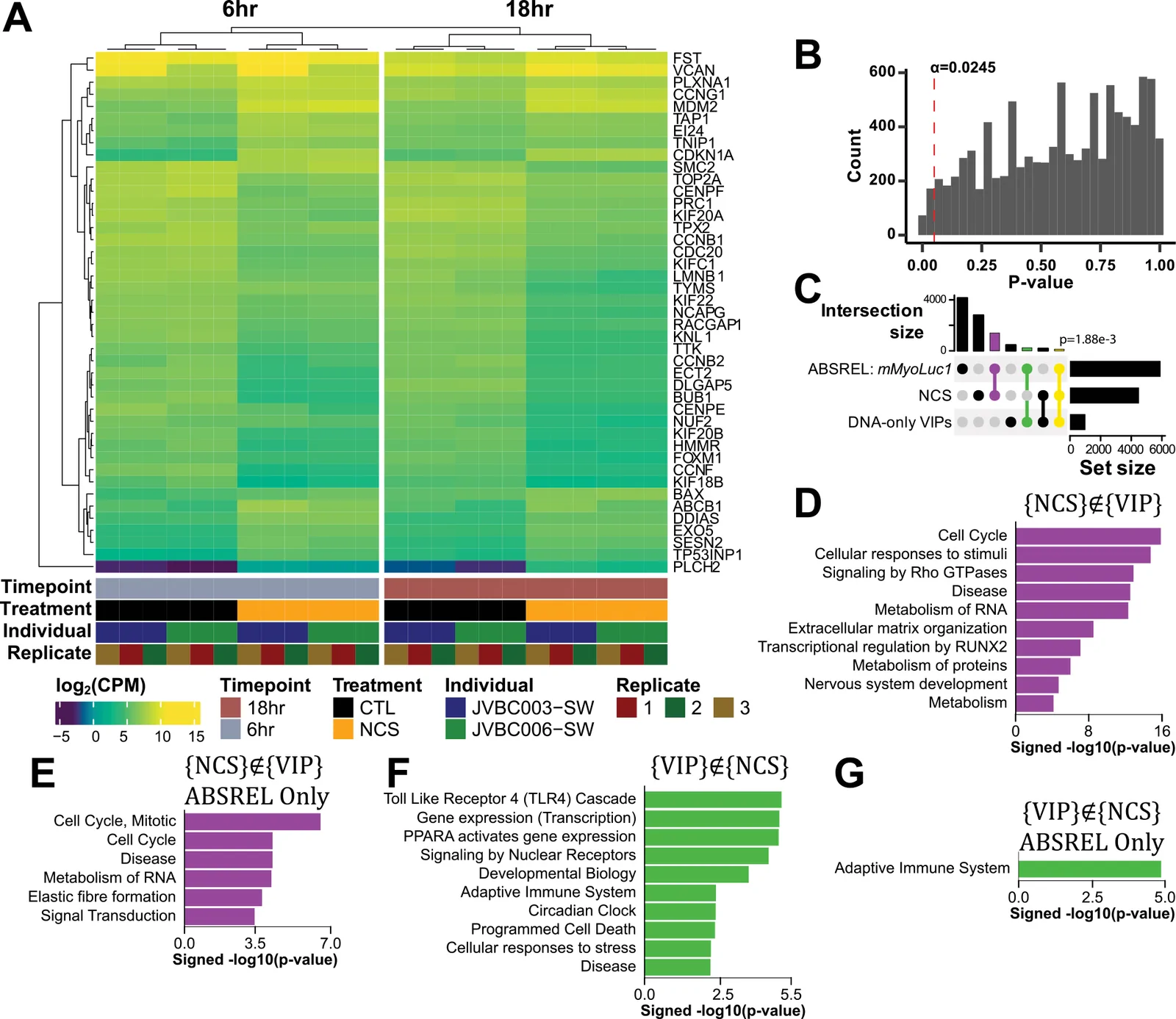
Genes differentially expressed after neocarzinostatin treatment and their overlap with DNA-only VIPs. **A**) Heatmap of log_2_ Counts Per Million (CPM) for the top 50 differentially expressed genes in *M. lucifugus* after 6- and 18-h of treatment with 100 nM neocarzinostatin. **B**) Histogram of p-values for the overlap of 10,000 simulated sets of non-NCS DE genes and non-DNA-only VIPs. The empirical false positive rate, **α**, is 0.0245. **C**) Upset plot of genes that are either 1) under selection (*ABSREL*, p_adj_ ≤ 0.05) in *Myotis lucifugus* ({*aBSREL*}); 2) differentially-expressed after neocarzinostatin treatment ({*NCS*}); or 3) are classified as DNA-only VIPs ({*VIP*}). The hypergeometric p-value for the set {*NCS*}∩{*aBSREL*}∩{*VIP*} is shown. **D-G**) Pathway-level overrepresentation analysis of genes that are exclusive to either {*NCS*} (**D, E**) or {*VIP*} (**F, G**) either before (**D, F**) or after (**E, G**) sub-settings for only those genes identified as under selection. Only pathways significant at FDR ≤ 0.05 are shown.

To better understand the genes that may be driving this difference, we examined RNA expression in *M. lucifugus* cells after 6 h and 18 h of treatment with 100 nM NCS. At 6 h we observed upregulation of genes associated with cell cycle arrest (such as *MDM2* and *CDKN1A*) and cell death (such as *BAX*), and downregulation of genes in pathways associated with cell division, growth and DNA damage repair and synthesis. At 18 h, we observed upregulation of genes in pathways associated with cellular stress response and continued downregulation of the cell cycle (Fig. 5a–d, Extended Data Fig. 7a and Supplementary Table 9). However, we observed that the strongest pathway enrichment in our DNA-damage RNA-sequencing data were genes associated with the innate immune response, including genes in the PI3K–AKT pathway (Fig. 5c,d and Supplementary Table 9). We hypothesized that the observed relationship between the unique DNA damage response of *M. lucifugus* may be due to pleiotropy with DNA VIPs. Indeed, we observed that there was a highly significant enrichment for genes that are differentially expressed after NCS treatment and DNA-only VIPs (*P* = 3.34 × 10^−5^, *α* = 0.0245; Fig. 5e and Extended Data Fig. 7b). The genes at the intersection of DNA-only VIPs and NCS response are over-represented in pathways associated with the cell cycle, senescence, transcription and DNA damage repair (Fig. 5f). We repeated these analyses considering only genes under positive selection in *M. lucifugus*. The intersection between DNA-only VIPs and NCS differentially expressed genes remained highly significant (*P* = 1.88 × 10^−3^; Extended Data Fig. 7c), with only the pathways cell cycle, DNA damage repair and deubiquitination enriched at false-discovery rate (FDR) ≤ 0.05 (Fig. 5g). DNA-only VIP genes, which are not NCS responsive, did not show these signatures (Extended Data Fig. 7d–g). Together these results suggest that selection on the innate immune response may drive agonistic pleiotropy in ageing-associated traits such as DNA damage response and cell cycle regulation.

## Discussion

In addition to evolving true flight, bats are notable for their long lifespan^8,33^, stress tolerance^39,49^ and viral tolerance^2,50^. The genes and mechanisms underlying these highly complex and pleiotropic phenotypes can be challenging to identify, especially for rapidly evolving phenotypes, such as host–pathogen interactions. Here we identify patterns of adaptation contributing to longevity, cancer resistance and viral interactions in bats, and demonstrate a unique DNA damage response in primary cells of the long-lived *M. lucifugus*.

The evolution of body size and lifespan across mammals has major implications for the co-evolution of cancer risk and resistance. We find altered scaling of longevity in *Myotis* that is predicted to have serious consequences for their intrinsic, per-cell cancer risk. Similar to other systems in which the evolution of cancer resistance has been driven largely by rapid changes in body size^36,51,52^, the rapid and repeated changes in lifespan across an order of magnitude observed in *Myotis* are hypothesized to result in strong selective pressures on lifetime cancer suppression to avoid malignancies^33,38,53^. This is supported by recent meta-analyses on cancer risk in vertebrates^39,54^ demonstrating little to no correlation between neoplasias and either species body size or lifespan, as well as by studies showing weak correlation or no correlation between mutation rates and either species body size or lifespan, respectively^55–57^.

Bats are also important models for understanding infectious disease response and immune adaptation owing to their tolerance to viruses and role as zoonotic reservoirs^13,27,58^. Here we show that, while bats have adapted to both DNA and RNA viruses, they have done so by different modes—protein evolution versus copy-number changes, respectively. This is in contrast to humans and other primates, in which protein coding adaptations to RNA viruses dominate^32,59^. Moreover, we demonstrate complex patterns of structural variation in immune genes, including a segregating duplication of *PKR*, which encodes a major protein involved in the antiviral innate immune system. *PKR* has functional relevance in its activity against both DNA and RNA viruses^21^. We further demonstrate the functional implications of copy-number variation in *PKR*, including an additive effect on cellular effector functions.

Multiple hypotheses have been proposed to connect the particular physiology and ecology of bats with the evolution of notable adaptations such as viral infection tolerance and defence, stress tolerance and exceptional longevity^3^. Many theories of the driving causes of disease resistance and longevity in bats have been proposed, including the evolution of flight^1,60–62^, the disposable soma hypothesis^63^, metabolic state^64^, torpor^8^ and other environmentally driven adaptations^65,66^. Moreover, many studies have highlighted the intersection of these traits, including links between hibernation, DNA repair, longevity^8,58^ and infectious disease resistance^8,27,58,67^. Our results support the importance of agonistic pleiotropy in shaping bat evolution, whereby genetic adaptations for many specific traits (such as DNA virus innate immunity) may prove beneficial to other seemingly unrelated traits (such as cancer resistance, cellular homeostasis and longevity). Importantly, bats are also the only known mammal to have active DNA transposons^68^, the suppression of which may also contribute to signatures of selection in DNA-interacting VIPs. Our findings on pervasive selection on DNA-only VIPs and the extreme evolution of longevity-associated in *Myotis* and other bats support a broader hypothesis that traits such as cancer risk, cellular homeostasis and antiviral response have evolved in tandem due to pleiotropic selection at coinciding points in the evolutionary history of bats.

## Methods

### Samples and materials

All sample collection efforts were performed according to approved animal use protocols by the University California, Berkeley and the University of Arizona. All samples collected in this study were collected under scientific collection permits from either the Arizona Game and Fish Department (SP405504, SP407155, SP403977 and SP407113) or the California Department of Fish and Wildlife (S-220230001-22025-001) (Supplementary Table 1). Bats were sampled using standard mist-netting procedures, including taking standard body measurements, following USGS recommendations for White-Nose Syndrome and COVID-19 prevention^71,72^. For *M. lucifugus*, the donor individual was field-caught in California and transported to the Genetics Laboratory of the California Department of Fish and Wildlife, where they were euthanized by isofluorane. The *M. velifer* individual was caught in Arizona and euthanized in the field by isoflurane. For both *M. lucifugus* and *M. velifer*, tissues were collected and preserved through flash-freezing in liquid nitrogen. All other genomes were generated from primary cell lines of heterogametic individuals whenever possible to ensure assembly of all sex chromosomes. Additional information can be found in the Supplementary Information and in Supplementary Table 1. All cell lines were tested routinely for mycoplasma contamination using MycoStrip (InVivogen).

All PKR experiments were performed using HeLa *PKR*-KO cells (provided by A. Geballe)^73^. The cells were maintained at 37 °C under 5% CO_2_ and cultured in DMEM supplemented with 5% FBS, 1% penicillin–streptomycin mix and 1 μg ml^−1^ puromycin (Sigma-Aldrich). All transfections were performed 24 h after seeding, using 3 µl of TransIT-LT1 Transfection Reagent (Mirus Bio) per 1 µg of DNA and Opti-MEM medium. We used previously generated pSG5-Flag×2 vectors encoding either *M. myotis* PKR1 (GenBank: OP006550), *M. myotis* PKR2 (GenBank: OP006559), *M. velifer* PKR1 (GenBank: OP006558) or *M. velifer* PKR2 (GenBank: OP006557)^21^. Plasmids encoding the interferon-stimulated gene* ISG20* (ref. ^70^) and a constitutively active variant of the sterile alpha-motif-domain-containing protein 9-like SAMD9L-F886Lfs*11 (referred to here as SAMD9L)^69^ were used as controls in viral infections and cell translation experiments, respectively.

### Near-complete genome assembly and annotation

Details on genome assembly, including DNA and RNA extraction, preparation of PacBio HiFi, Omni-C (Dovetail Genomics) and RNA-sequencing libraries, genome assembly, annotation and manual curation, are provided in the Supplementary Information.

### Structural variation

To understand the genomic distribution of SVs, including segmental duplication events, we used SyRI (Synteny and Rearrangement Identifier^19^). After masking repetitive regions such as telomeres and centromeres, the primary 22 scaffolds corresponding to the autosomes of the Nearctic *Myotis* genomes were mapped to each other in the correct orientations using minimap2 (ref. ^74^). We ran SyRI on the resulting files and plotted the results with plotsr^75^.

### Phylogenetics

A phylogeny of all 536 mammals in our alignments was generated using IQTREE^76^ (v.2.3.1) using all gene alignments with the settings ‘-B 1000 -m GTR+F3x4+R6’. Gene trees were generated from gene alignments to exclude alignments with less than 50% gaps in the sequence and 4 or more species represented by using IQTREE with the settings ‘--wbtl --bnni --alrt 1000 -B 1000 --safe’. The best substitution models for each gene were saved as a NEXUS file. As the bootstrap values for this tree were unanimously 100%, we ran ASTRAL-IV^77^ (v.1.24.4.7) using the settings ‘-t 54 -u 2 -C’ and confirmed that our phylogeny agreed with other previously published *Eutherian* phylogenies^78–80^. The Chiroptera portion of our phylogeny was time calibrated using MCMCtree^81^ and PAML^82^ (v.4.10.0) with the bat-subset of our codon alignments and using fossil calibrations^79,83–91^ (Supplementary Table 2). We ran MCMCtree twice to generate the Hessian matrix and confirm convergence, and ran ten independent chains using the out.BV file from the first run. Finally, the output files of all ten chains were combined to compute the final divergence time estimates.

### Ancestral body size and lifespan reconstruction

To examine how body size and lifespan have evolved over time in mammals, we used a super-phylogeny of mammal species^34^ subsampled to contain only species with extant body size and lifespan data collected from AnAge^15^ and PanTHERIA^92^. Ancestral body sizes and lifespans were simulated separately using StableTraits^93^ and the settings ‘--iterations 100000 --skip 1000 --chains 8’. Estimates were further validated by comparing our initial results to a second run of StableTraits using the same settings.

### Selection scans and evolutionary rates

#### aBSREL

To conservatively test for branch-specific selection, we used aBSREL^41,94^ (v.2.5.48) to test for selection at each branch within the Nearctic *Myotis* clade for 15,734 gene alignments spanning 536 mammals. These genes were identified as 1:1 orthologues across the full alignment, with no more than 50% sequence gaps and at least 4 species present in the alignment. For each gene alignment, the full phylogeny was trimmed to match the species present, and the HyPhy script label-tree.bf was used to highlight all nodes within Nearctic *Myotis* as foreground branches. aBSREL was then run using the pruned and highlighted tree and gene alignments on the command line with the options ‘--code Universal --branches Foreground’. We defined a gene as under selection if one or more regions within the gene demonstrated a selective pressure with an FDR-corrected* P* ≤ 0.05. A gene was specifically identified as under positive selection if at least one of these significant regions had an *ω* > 1.

#### BUSTED

To quantify the total amount of positive selection across the *Myotis* tree or the different species trees used in this Article, we used an improved version of the BUSTED^25,94^ test called BUSTED-MH (details on BUSTED versus BUSTED-MH are provided in the Supplementary Information). We applied BUSTED-MH to 19,646 *Myotis* orthologous CDS alignments with at least five orthologues. These orthologues are cases in which the Orthofinder gene trees coincide with the species tree. This effectively removes issues regarding whether we should use the gene or the species tree, at the cost of removing 2,110 genes from the *Myotis* selection analysis. Similarly, we applied BUSTED-MH to 17,469 non-*Myotis* bat alignments with at least five orthologues. The species in these two sets (*Myotis* bats and non-*Myotis* bats) are mutually exclusive. This includes a subset of 14,091 alignments with orthologues present in two thirds of the non-*Myotis* bat species, regardless of the genes’ presence/absence in the *Myotis*-only complement. This is specifically used to show that patterns of virus-driven adaptation are representative of all bats. We also tested 17,890 primate alignments with at least five orthologues with BUSTED-MH, as well as 19,311 Glire, 18,000 Carnivora and 18,504 ungulate alignments. Similarly, HyPhy MEME (with --resample 100 to increase power) was run to identify individual codon sites subject to diversifying selection in the *CD45* gene for visualization with host protein–virus contact sites.

#### Enrichment scores for VIPs

We expanded on the set of VIPs reported previously^26^ (5,291 VIPs) by searching the literature for more recent publications reporting VIPs, with a final dataset of 5,527 VIPs. These VIPs (all VIPs) were further subcategorized into four groups on the basis of their interactions with either DNA viruses and/or RNA viruses: DNA VIPs, DNA-only VIPs, RNA VIPs and RNA-only VIPs (Supplementary Table 5). For each VIP set, we selected sets of matching control genes by controlling for 16 different. Sets of control genes were resampled in a bootstrap procedure to generate 95% confidence intervals for sets of genes across a range of *P* values; additional details are provided in the Supplementary Information.

#### Gene duplications and CAFE analysis

To quantify patterns of gene duplication and loss, we quantified the copy number of genes with human orthologues from our gene annotations for each Nearctic *Myotis* genome. To calculate per-gene expansion and loss rates and their statistical significance, we ran CAFE^95^ (v.5) on the previously described set of copy-number counts using our time-calibrated species tree pruned to include only the nine Nearctic *Myotis* species. CAFE uses a birth-death model of gene family evolution to investigate changes in gene family size accounting for evolutionary history using the species phylogeny. Evolutionary branch length is incorporated into the model expectations, therefore accounting for potential bias resulting from differences in branch lengths. We ran CAFE on the subset of genes with two or more copies in at least one species using a Poisson distribution for the root frequency (*-p*), first generating an error model to correct for genome assembly and annotation error (*-e*). We compared the base model (each gene family belongs to the same evolutionary rate category) to gamma models (each gene family can belong to one of *k* evolutionary rate categories) with different values of *k* from 2 to 13. A final gamma model with *k* = 9 was chosen to balance model log likelihood with the number of gene families for which the optimizer failed. The model was run three separate times to ensure convergence.

To understand whether genes in these pathways have higher birth–death rates or are more likely to have significant changes in gene copy number compared with that expected relative to other genes, we evaluated the gene copy birth–death rate *λ* and the number of genes significantly expanded or contracted in copy number on at least one branch within our Nearctic *Myotis* phylogeny using a bootstrap procedure. Following ref. ^31^, we tested whether VIP genes in particular underwent significant copy-number changes or had significantly different birth-death rates than non-VIP genes. For each category of VIP genes (all VIPs, DNA VIPs, DNA-only VIPs, RNA VIPs and RNA-only VIPs), we generated 100 bootstrap sets of control non-VIP genes with the same number of genes as the corresponding VIP gene set. We ran CAFE on each set of VIP genes and the corresponding control non-VIP genes to infer per-gene birth–death rates and per-gene, per-branch expansion/loss events.

### Assessment of DNA double-stranded break tolerance

We assessed each species’ tolerance to DNA double-stranded breaks by measuring viability, cytotoxicity and apoptosis across a range of doses of NCS, a radiomimetic drug. We measured dose–response curves in wing-derived primary dermal fibroblasts across five bat species (*M. lucifugus*, *n* = 8; *M. evotis*, *n* = 8; *R. lanosus*, *n* = 2; *E. helvum*, *n* = 2; *P. rodricensis*, *n* = 2) using the multiplexed ApoTox-Glo assay (Promega). Using 96-well plates, 2 individuals and 11 doses were assessed simultaneously with 4 technical replicates. The results were normalized to treatment controls for each individual in R (Code availability).

### PKR1/2 characterization in *PKR*-KO HeLa cells

#### Western blot

Details on PKR western blotting are provided in the Supplementary Information.

#### Luciferase reporter assays

Details on PKR luciferase reporter assays are provided in the Supplementary Information.

#### PKR co-IP

HeLa *PKR*-KO cells were transfected with 1.25 µg plenti6 HA-tagged *M. myotis* PKR1 plasmid per million cells and 1.25 µg of either plenti6 MYC empty vector, MYC-tagged *M. myotis* PKR1 or MYC-tagged *M. myotis* PKR2 plasmid using TransIT-LTI transfection reagent (Mirus Bio). The next day, some wells were infected with Sindbis virus expressing GFP (SINV-GFP) at a multiplicity of infection (MOI) of 2 for 24 h. Cells were then scraped with cold PBS and pelleted. For the IP, cells were lysed in 500 µl IP buffer (50 mM Tris HCl pH 7.5, 140 mM NaCl, 6 mM MgCl_2_, 0.1% NP40) supplemented with RNase (RiboLock, Fisher Scientific) and protease (complete EDTA-free protease inhibitor cocktail, Sigma-Aldrich) inhibitors for 10 min on ice, then centrifuged at 12,000*g* for 10 min at 4 °C. In total, 5% of the volume was kept for input, and the rest was incubated with 40 µl µMACS anti-MYC MicroBeads (Miltenyi Biotec) for 1 h at 4 °C with constant rotation. The samples were then loaded onto µMACS columns placed in the magnetic field of a µMACS Separator (Miltenyi Biotec), washed four times with cold IP buffer and eluted with the µMACS denaturing elution buffer. Proteins were denatured in elution buffer for 5 min at 95 °C, then loaded onto a 4–20% BioRad Criterion TGX Stain-Free precast gel and transferred onto an Amersham Protran nitrocellulose membrane (Sigma-Aldrich) for 1 h. Membranes were blocked for 1 h in 5% milk in PBS (Euromedex) supplemented with 0.2% Tween-20 (Thermo Fisher Scientific) and were incubated with mouse anti-MYC monoclonal antibodies (Abcam, 9E10, ab32), then secondary anti-mouse IgG antibodies conjugated with HRP (Sigma-Aldrich, A4416), or with rat anti-HA antibodies conjugated to HRP (Roche, Sigma-Aldrich, 12013819001). Images were taken on the Fusion FX imager (Vilber) with SuperSignal West Femto Chemiluminescent Substrate (Thermo Fisher Scientific).

#### Cell viability assay

HeLa *PKR*-KO cells were transfected 24 h after plating in 96 well plates (*n* = 10,000 cells per well), with 100 or 200 ng of pSG5 plasmid: empty or coding for *M. myotis* or *M. velifer* PKR1, PKR2 or PKR1 + 2 equal mix (50%–50%). At 24 h after transfection, positive control cells were treated with an apoptosis-inducing drug (etoposide) at different doses (250, 200 or 100 µM). Then, at 48 h after transfection, cells were collected and lysed to quantify the luminescence signal according to CellTiter-Glo Luminescent Cell Viability Assay (Promega) kit protocol.

#### VSV and SINV infections

##### VSV infections

In total, 200,000 cells were plated in 12-well plates and transfected 24 h after with 350 ng of pSG5 plasmid: empty; encoding *M. myotis* or *M. velifer* PKR1, PKR2 or equal input of PKR1 and PKR2 (175 ng per plasmid); or a plasmid encoding ISG20, due to its known antiviral activity against VSV as positive control^70^. Cells were infected at 24 h after transfection with replicative VSV-GFP virus^96^ at a MOI of 3. Cells were fixed with 4% paraformaldehyde at 16–18 h after infection. VSV infection was quantified by measuring the percentage of GFP-positive cell populations using the BD FACSCanto II Flow Cytometer (SFR BioSciences). Fold change results were normalized to the empty pSG5 condition across at least three independent experiments.

##### SINV infections

HeLa *PKR*-KO cells were transfected with 5 µg pSG5 empty vector, *M. myotis* PKR1, *M. myotis* PKR2 or 2.5 µg *M. myotis* PKR1 + 2.5 µg *M. myotis* PKR2 per million cells using TransIT-LTI transfection reagent (Mirus Bio). The next day, some wells were infected with SINV-GFP at MOI 0.2. Cells were then placed into the CellCyte X live cell imaging system (Cytena) and pictures of every well were taken every 2 h for 48 h. The fraction of GFP^+^ cells over the total cell area was measured and averaged from 6 photos of 2 individual wells per condition, and repeated for a total of 3 independent experiments.

### Reporting summary

Further information on research design is available in the Nature Portfolio Reporting Summary linked to this article.

## Online content

Any methods, additional references, Nature Portfolio reporting summaries, source data, extended data, supplementary information, acknowledgements, peer review information; details of author contributions and competing interests; and statements of data and code availability are available at https://doi.org/10.1038/s41586-026-10932-7.

## Supplementary information

Supplementary Information Supplementary Methods, Supplementary Discussion, Supplementary Figures and Supplementary References.

Reporting Summary

Supplementary Tables Supplementary Tables 1–9.

Peer Review File

## Data Availability

All sequencing data and genomes generated in this study are available under NCBI BioProject accessions PRJNA973719, PRJNA1035541 and PRJNA1295526. Annotations generated in this study are available at GitHub (https://github.com/docmanny/myotis-gene-annotations). All other data are archived on Dryad^97^ (https://doi.org/10.5061/dryad.02v6wwqfh).

## Code availability

All custom code used herein is available at GitHub (https://github.com/sudmantlab/MyotisGenomeAssembly).
