# Supplementary material for: Insights into longevity and virus-driven adaptation from Myotis bat genomes
Source: Nature. 2026 Aug 26;658(8134):153–62. doi: 10.1038/s41586-026-10932-7 (PMC13626957; doi:10.1038/s41586-026-10932-7)
Supplement: Supplementary file 2 [file 41586_2026_10932_MOESM2_ESM.pdf]

nature portfolio

Corresponding author(s): Sudmant*, Enard, Etienne

Last updated by author(s): Jun 30, 2026

Reporting Summary

Nature Portfolio wishes to improve the reproducibility of the work that we publish. This form provides structure for consistency and transparency in reporting. For further information on Nature Portfolio policies, see our Editorial Policies and the Editorial Policy Checklist.

Statistics

For all statistical analyses, confirm that the following items are present in the figure legend, table legend, main text, or Methods section.

n/a	Confirmed	
☐	☒	The exact sample size (*n*) for each experimental group/condition, given as a discrete number and unit of measurement
☐	☒	A statement on whether measurements were taken from distinct samples or whether the same sample was measured repeatedly
☐	☒	The statistical test(s) used AND whether they are one- or two-sided<br>*Only common tests should be described solely by name; describe more complex techniques in the Methods section.*
☐	☒	A description of all covariates tested
☐	☒	A description of any assumptions or corrections, such as tests of normality and adjustment for multiple comparisons
☐	☒	A full description of the statistical parameters including central tendency (e.g. means) or other basic estimates (e.g. regression coefficient) AND variation (e.g. standard deviation) or associated estimates of uncertainty (e.g. confidence intervals)
☐	☒	For null hypothesis testing, the test statistic (e.g. *F*, *t*, *r*) with confidence intervals, effect sizes, degrees of freedom and *P* value noted<br>*Give P values as exact values whenever suitable.*
☐	☒	For Bayesian analysis, information on the choice of priors and Markov chain Monte Carlo settings
☒	☐	For hierarchical and complex designs, identification of the appropriate level for tests and full reporting of outcomes
☒	☐	Estimates of effect sizes (e.g. Cohen's *d*, Pearson's *r*), indicating how they were calculated

Our web collection on statistics for biologists contains articles on many of the points above.

Software and code

Policy information about availability of computer code

Data collection	All code and programs used in this manuscript, along with source, versions used, and settings tested, are described in detail in the Methods and Supplemental Methods of this manuscript. All custom scripts have been made available on GitHub and Zenodo at links listed in the Methods.
Data analysis	All code and programs used in this manuscript, along with source, versions used, and settings tested, are described in detail in the Methods and Supplemental Methods of this manuscript. All custom scripts have been made available on GitHub and Zenodo at links listed in the Methods.

For manuscripts utilizing custom algorithms or software that are central to the research but not yet described in published literature, software must be made available to editors and reviewers. We strongly encourage code deposition in a community repository (e.g. GitHub). See the Nature Portfolio guidelines for submitting code & software for further information.

Data

Policy information about availability of data

All manuscripts must include a data availability statement. This statement should provide the following information, where applicable:

- Accession codes, unique identifiers, or web links for publicly available datasets
- A description of any restrictions on data availability
- For clinical datasets or third party data, please ensure that the statement adheres to our policy

All sequencing data and genomes generated in this study are available on NCBI under Bioprojects PRJNA973719, PRJNA1035541, and PRJNA1295526. Annotations generated in this study are available at https://github.com/docmanny/myotis-gene-annotations. All other code is available at https://github.com/sudmantlab/MyotisGenomeAssembly. All other data are archived on DataDryad at DOI: 10.5061/dryad.02v6wwqfh.

Research involving human participants, their data, or biological material

Policy information about studies with human participants or human data. See also policy information about sex, gender (identity/presentation), and sexual orientation and race, ethnicity and racism.

Reporting on sex and gender	N/A
Reporting on race, ethnicity, or other socially relevant groupings	N/A
Population characteristics	N/A
Recruitment	N/A
Ethics oversight	N/A

Note that full information on the approval of the study protocol must also be provided in the manuscript.

Field-specific reporting

Please select the one below that is the best fit for your research. If you are not sure, read the appropriate sections before making your selection.

☒ Life sciences ☐ Behavioural & social sciences ☐ Ecological, evolutionary & environmental sciences

For a reference copy of the document with all sections, see nature.com/documents/nr-reporting-summary-flat.pdf

Life sciences study design

All studies must disclose on these points even when the disclosure is negative.

Sample size	For functional experiments, the sample sizes for number of individuals per species were selected based on the availability of cell lines for testing. For all species and conditions, at least two biological replicates (distinct, unrelated individuals) were used, and all measurements were taken in replicate (n=3-5) based on the standards of the field for the specific type of experiment. When possible, we controlled for sex and geographic distribution of each species by binning individuals based on these criteria to generate mutually exclusive lists, from which we then randomly selected an equal number of individuals from each list.
Data exclusions	Original manuscript had no data exclusions - 100% of data generated for the manuscript was featured. After a reviewer comment suggesting that one outlier may have driven the statistical significance of one analysis, we omitted this individual from the analysis used in the revised manuscript and figure (Figure 5F) and found no change in our results. All original data was still preserved in the supplement and is still available on our Dryad archive.
Replication	For comparisons on species-specific traits, at least two biological replicates (distinct, unrelated individuals) from each species were used, all measurements were taken in replicate (n=4), and the experiment was performed in triplicate. For experimental validation of PKR, all experiments were done in triplicate; the luciferase assays were done in quintuplicate due to the additional stochasticity of this assay.
Randomization	Individuals from each species were randomly selected from available cell lines by randomly sampling them from a list using a computer. When possible, we controlled for sex and geographic distribution of each species by binning individuals based on these criteria to generate mutually exclusive lists, from which we then randomly selected an equal number of individuals from each list.
Blinding	Each cell line is identified only with an alphanumeric sample that provides no indication of which individual or species it is. Experiments are done using only knowledge of these identifiers, and only after data generation are researchers aware of metadata for each sample.

Reporting for specific materials, systems and methods

We require information from authors about some types of materials, experimental systems and methods used in many studies. Here, indicate whether each material, system or method listed is relevant to your study. If you are not sure if a list item applies to your research, read the appropriate section before selecting a response.

Materials & experimental systems

n/a	Involved in the study
☐	☒ Antibodies
☐	☒ Eukaryotic cell lines
☒	☐ Palaeontology and archaeology
☐	☒ Animals and other organisms
☒	☐ Clinical data
☒	☐ Dual use research of concern
☒	☐ Plants

Methods

n/a	Involved in the study
☒	☐ ChIP-seq
☒	☐ Flow cytometry
☒	☐ MRI-based neuroimaging

Antibodies

Antibodies used: Mouse anti-Flag (Sigma F3165); mouse anti-Tubulin (Sigma T51168); anti-mouse IgG-peroxidase conjugated (Sigma A9044); mouse anti-myc monoclonal (Abcam 9E10, cat# ab32, Lot# GR3421642-4); anti-mouse IgG antibodies conjugated with HRP (Sigma A4416); rat anti-HA antibodies conjugated with HRP (Merck, Sigma cat# 120138190).

Validation: All antibodies used were validated by the manufacturer.

Eukaryotic cell lines

Policy information about cell lines and Sex and Gender in Research

Cell line source(s): All sample collection efforts were performed following approved Animal Use Protocols by the University California, Berkeley and the University of Arizona. All skin punch biopsies in this study were collected under Scientific Collection Permit numbers SP407155, SP403977, SP407113 (Arizona Game and Fish Department), and S-220230001-22025-001 (California Department of Fish and Wildlife). Two 3-mm wing punch biopsies were taken from the left and right plagiopatagium of each donor individual and placed in a live cell collection media consisting of DMEM/F12 (Gibco) supplemented with 15mM HEPES (Gibco), 20% FBS (Gibco), and 0.2% Primocin (Invivogen). These wing punches were then brought back to a cell culture facility in Berkeley, where they were used to generate cell lines.
Cell lines for the M. evotis and M. thysanodes genomes were generously provided by Richard Miller. Cell lines for functional work in Rousettus langosus, Pteropus rodrigensis, and Eidolon helvum were provided by the San Diego Frozen Zoo.

Authentication: Cell lines used to generate reference genomes were validated by sequencing and comparing the assembled mitochondrial genomes to published references on NCBI. Other cell lines were authenticated by species based on sequencing of an amplicon in the gene CoI.

Mycoplasma contamination: All cell lines tested negative using the Mycostrip Mycoplasma Detection Kit (InVivogen).

Commonly misidentified lines (See ICLAC register): N/A

Animals and other research organisms

Policy information about studies involving animals; ARRIVE guidelines recommended for reporting animal research, and Sex and Gender in Research

Laboratory animals: N/A

Wild animals: Bats were sampled using standard mist-netting procedures, including taking standard body measurements, following published USGS recommendations for White-Nose Syndrome and COVID-19 prevention. Nets were checked every 5-8 minutes to ensure bats were recovered quickly upon capture. Bats were detained and handled for no longer than 30 minutes, with most individuals being released after 10 minutes. After species identification, one 3-mm skin punch biopsies were taken from each wing of an individual after sterilization of the biopsy site using 70% isopropyl alcohol. Individuals were released away from the nets after sampling and watched to ensure safe flight and exit from the area.

Reporting on sex: Findings are general to species and not specific to sex. Prior to experimentation, an equal balance of males and females (as determined by donors' physical characteristics) was selected whenever possible; however, this was restricted based on the random selection of individuals captured or the source of cell lines. For reference genomes, males were selected whenever possible to ensure that both sex chromosomes were accounted for in the data.

Field-collected samples	See "Wild animals".
Ethics oversight	All sample collection efforts were performed following approved Animal Use Protocols by the University California, Berkeley and the University of Arizona. All samples collected in this study were collected under Scientific Collection Permit numbers SP405504, SP407155, SP403977, SP407113 (Arizona Game and Fish Department), and S-220230001-22025-001 (California Department of Fish and Wildlife).

Note that full information on the approval of the study protocol must also be provided in the manuscript.

Plants

Seed stocks	N/A
Novel plant genotypes	N/A
Authentication	N/A
