# Supplementary material for: Insights into longevity and virus-driven adaptation from Myotis bat genomes
Source: Nature. 2026 Aug 26;658(8134):153–62. doi: 10.1038/s41586-026-10932-7 (PMC13626957; doi:10.1038/s41586-026-10932-7)
Supplement: Supplementary file 3 [file 41586_2026_10932_MOESM4_ESM.pdf]

nature portfolio

Peer Review File

Insights into longevity and virus-driven adaptation from Myotis bat genomes

Corresponding Author: Professor Peter Sudmant

Any redactions in this file are there to maintain patient confidentiality, the confidentiality of unpublished data, or to remove third-party material.

This file contains all reviewer reports in order by version, followed by all author rebuttals in order by version.

Version 0:

Reviewer comments:

Referee #1

(Remarks to the Author)
This study focuses on the genus Myotis, a group of bats known for their large variation in lifespan and adaptations to viruses. The authors generated cell lines and high quality genome assemblies for eight Myotis species. They performed extensive analyses that provided evidence of positive selection linked to longevity, cancer resistance, and viral interactions. The findings regarding adaptations to DNA but not RNA viruses are very interesting. Functional cell experiments were also conducted, although I have questions about both experiments.

Overall, this manuscript provides important new resources and a number of interesting results.
I have several questions and need clarifications on the analyses and the experiments.

- l118: Please define auNG as this is a not widely used measure (so far).

- l143: 100% bootstrap support is typically observed with large datasets. To support the correctness of the phylogenetic tree inferred with IQtree and concatenated alignments, coalescence methods should be used. Node support can be measured as the proportion of gene trees that support the species tree.

- l170: Are myotis species statistical outliers in this analysis?

- Reduced Intrinsic Cancer Risk per cell.
From the DT^6 equation in the methods, it is clear that if D=body size stays the same and T=lifespan doubles, the risk increases log2(2^6) = 6fold. It is good to quantify this, but given this equation and what we know about Myotis species, I find it a rather trivial result that some myotis species have a large RICR variation.

Moreover, I find the double usage of "reduced" difficult to understand in sentences like "Decreases in (Reduced Intrinsic Cancer Risk) correspond to an increase in the expected cancer risk" or "decreases in RICR" or "bottom 10% of RICR". I would suggest to speak of the ICR, as we all understand what it means if ICR increases or decreases, or if ICR is high or low.

- l200: On average 22.7% of genes were under selection across the 9 nearctic Myotis species. If this analysis also considered internal and not just the terminal branches, what does the average refer to?
Moreover, 22.7% is a rather high percentage for a group of nine related species.

- l200: The methods describe that 70 primates, 138 euungulates, 127 glires, 82 carnivora and 47 pan-chiroptera species were included. This sums to 464 but not 536 species. Please clarify.

Several important datasets including the list of these 536 or 464 species and the orthologous gene alignments are also missing.

- l202 and l1624 and Fig 3A: Since aBSREL fits up to three omega values to model codon evolution. I suppose what is meant with omega > 1 in this line is not the overall genewise omega but one of the three omega values? It would then be important to also specify the proportion of sites that evolve with the omaga > 1. This should be clarified in the text as well as the figure, maybe by plotting the proportion as a color code.

- l208: How are cancer-related pathways defined? Looking at table S3, I can see for some Myotis species gene sets labeled as cell cycle or other relevant terms, but it is not obvious that the enrichments shown are strongly related to cancer.

I also wonder why gene ontology terms were not tested?

- l218: Table S3 shows ferroptosis as a word to occur in the first sheet, which is unclear as this sheet is listing the genes and not the enriched gene sets. The other sheets listing the enriched pathways per clade do not show ferroptosis.
Moreover, the organization of the supplemental tables needs to be improved, including clearly labeling columns, stating where to find what data and what the different sheets mean. This also applies to other tables. For example, what is RERC PC2 ORA in Table S4?

- l255: Figure 3D shows for apoptosis and myotix lucifugus four outlier values with a fold change of 5-6 while the other data points are around a fold change of 2. The confidence intervals do not reflect these 2-3 fold increase for some of the replicates. There is no table with the raw values, therefore I could not check if this is a plotting error.
If this is not a plotting error what explains that this large deviation that was only observed for M. lucifugus. Important, would removing these outliers render the difference to other species not significant?

- There are 5527 VIPs listed in the supplemental table. This is 25% of all genes. If these proteins truly physically interact with viruses as stated in the manuscript, then many of these proteins likely have no effect on the virus and binding is coincidental or biologically not relevant. This is should be made clearer in the manuscript.

- l339: If DNA transposable elements catalyze large-scale rearrangments, why would they be depleted at the inversion boundaries?
I also struggle to see the depletion in figure 5B, which does not show the narrower windows of inversion boundaries. Rather, what seems to be obvious is that regions with more segmental duplications overlap LINE peaks.

- l345: How are the gene families for the CAFE analysis defined? This is not described in the methods.

- l366: The FBXO31 expansion may be an error. Ensembl orthologs list many additional copies of this gene in Myotis lucifugus, but they seem all be intronless retrogenes. These genes may have a function though (https://www.nature.com/articles/s41420-023-01348-7). However, it should be carefully assessed whether the reported copies in other Myotis species are segmental duplications or retrogenes and, if the latter, whether the retrogenes are transcribed and translated.

- l413: I fail to see the additive effect in Figure 6E. PKR1+2 has a less efficient or similar translation shutdown compared to PKR1 alone. Additive effects should result in a stronger shutdown for PKR1+2, which is not visible in the figure.

- l408: I am not convinced that HeLa cells are an appropriate system for these experiments, also because the authors fail to find a strong effect. These experiments should be conducted in the Myotis cell lines that the authors have generated.

- l418-422: The interpretation that high doses of PKR led to cell toxicity would only be relevant for Myotis species if they express PKR1 and PKR2 together at levels similar to those in the overexpression assays. To reach a relevant conclusion, one needs to compare the expression levels in the cell assay to those in Myotis tissues.

- l1641: I assume the 19,646 Myotis orthologous CDS alignments and the 17,469 non-Myotis bat alignments contain distinct species, as otherwise the signal from shared species would be considered in both datasets. This should be stated in the methods.
Moreover, are the number of species in these CDS alignments matched?

- l1578: What are supplementary files XY ?
- l1966 typo xo-expression
- figure S3 is very blurry and not readable
- typo in l156 Yinpterochrioptera

(Remarks on code availability)

Referee #2

(Remarks to the Author)
The study by Vasquez et al presents a comprehensive set of computational analyses based on the sequencing of eight bat genomes from the genus Myotis. These genomes allow the authors to resolve the previously unclear phylogeny of Myotis. Additionally, the authors examine the relationship between lifespan and body size, finding evidence of rapid evolution

toward increased lifespan. Similar to other vertebrates with extreme changes in body size and lifespan, the authors identify strong selection for cancer resistance mechanisms at multiple instances within Myotis. Through selection scans, the study finds associations with genes involved in cancer, DNA repair, immunity, and metabolic processes. The scans also suggest that DNA viruses have exerted stronger selective pressures than RNA viruses. Lastly, structural variation analyses reveal that gene duplications are concentrated in pathways related to cancer, aging, immunity, and olfaction.

The manuscript constitutes a valuable genomic resource due to the high quality of the genome sequences (haplotype-resolved, chromosome-scale assemblies) and the importance of bats as a model organism. While the high-quality genomes allow for more thorough comparative analyses than those previously performed in bats, the approaches used here are standard in comparative genomics. The main traits studied, such as cancer resistance and lifespan, have already been reported as displaying unique patterns in bats. Consequently, the results, while valuable and solid, are not particularly novel.

I find the introduction and overall tone of the paper to be misleading. It emphasizes bats as a unique model system because it is a group in which one can combine comparative and functional/mechanistic approaches, creating an expectation that this study will do so. Rather than focusing on a specific result for in depth functional exploration, as the narrative suggests, the manuscript presents a broad collection of analyses that identify general evolutionary patterns. The results from these analyses are then presented and correlated with various life history traits, without any attempt to delve deeper into the underlying mechanisms. Doing so would have been impactful, and would have provided significant insights into the biology of this group, beyond what is already known. Instead, the reader is left wondering whether the many genes/pathways identified have any real relevance to the traits being examined, and whether they go beyond the correlations presented.

There are two functional studies presented, one showing that M. lucifugus has evolved an enhanced DNA double-strand break response and the other one examining structural differences in the PKR locus. The first one, while interesting, confirms a pattern seen in the genomic data but does not offer any insight into mechanism. Moreover, the results presented in Fig 3D, particularly for viability, show that the differences are quite mild, leaving the reader wondering about the relevance of this observation.

The second one -examining structural differences in the PKR locus- is underwhelming for a couple of reasons: First, the bat-specific structural differences at PKR had already been identified in a previous study examining bat-viral interactions, so this is not a novel finding from this study. The current work examines the combined effects of PKR1 and PKR2, as opposed to independent effects, as examined in the other published study. I consider this addition incremental. This begs the question: Why didn't the authors carry out functional/mechanistic exploration of one of the many genomic correlates identified in this particular study, instead of using a structural variant that was already known? I think this is a missed opportunity to show that bats are indeed an ideal group to carry out complementary genomic and functional studies, as the authors argue. Second, the experiments were performed in (human) HeLa cells. Do HeLa cells accurately reflect the interactions with the virus in a bat system? Are these the cells that come into contact with the viral proteins upon entry into the body? Are these the most relevant cells to carry out these experiments in? It seems that these cells were chosen simply as a 'blank slate' to test the effects of PKR duplicates. This is ok, and many studies do this, but then this has to be explicitly stated and the narrative adjusted because I don't consider this to be an experiment reflecting the fact that bats are good models for functional studies.

In summary, while the study provides valuable resources and can be used as a starting point to formulate/test hypotheses, it lacks the mechanistic depth and novelty. In my opinion, it would be better suited to a specialized evolutionary genomics journal.

(Remarks on code availability)

Referee #3

(Remarks to the Author)
The authors have characterized myotis specific immune features in bats associated with DNA damage responses and longevity and correlate what they believe are the genes linked to DNA viruses - matched to exceptional longevity. This is indeed intriguing. The addition of TE analysis etc also highlights some intriguing possiblities related to choromosmal aberrations/insertions/SVs etc and how the bats may respond to them with DNA damage machinery (I feel this is not highlighted enough). They then consequently show additional about the myotis PKR's they have already characterized but I find this in contrast to the claims of DNA-only VIPs being incredibly important in myotis (as PKR is not a DNA-only VIP, but rather an RNA-only VIP - in terms of direct interaction).

The authors should be careful with references, making sure the most appropriate reference is used - and correct. There's a reference to the author correction rather than the original article.
The comment about comparison of tissue vs cell genomes is interesting but has no associated data.
Figure 1 is a little crowded and the Figure legends do not have enough detail carefully explaining each graph and what it represents.
The Genome annotations appear to be using old versions of the pipelines (e.g. TOGA) so its important to mention version number.
Was it truly 27000 coding genes identified or 27000, isoforms?
The eutheria max likelihood tree should be supplied together in the supp and is there previous validation/other literature confirming time calibration methodology (if so, reference it).
The exceptional examples should be highlighted visually on the lifespan tree in Figure 2 somehow.

I am concerned about the conclusions drawn for figures 2 E/F - the RICR was modelled by the assumption that lifespan or bodysize directly correlate to a constant for cancer risk. The authors are now claiming that lifespan separates out disproportionately for cancer risk in bats (therefore breaking the assumption fo how the RICR was originally modelled)?

It would be important to ensure there is no bias introduced by the length of the phylogenetic tree (and ccumulation of mutations etc) and it is directly a function of the difference between e.g. lifespan/bodysize of species X and its common ancestor.

22.7% of all genes (single copy orthologs) being under selections seem quite high - what would be the expected number of genes be (relative to time/distance of the phylogenetic branch)? This could completely change the results simply based on evolutionary time between species.
There are very methods in the details for the absrel analysis, including which settings/model were chosen and the justification for why. - immunity, cancer and aging are three processes yet you talk about the intersection of two processes ??
Relax and RERconverge were very poorly described in the main text with no real justification for their usage etc (you cannot just explain in the methods but the main text requires some commentary).
Many of these genes have already been published as being under selection in bats - there needs to be a more consistent matching of the findings with existing literature.

The differences in apoptosis and viability between the cell lines is very minimal and as there is only one representative cell line for each species it is essential to show there is a true species-level effect and not a cell-line-specific defect. It has already bee shown that bats express varying levels of ABC transporter proteins affecting uptake of DNA damage reagents (e.g. ABCB1), it would therefore be essential to show levels of these proteins between cells and prove with multiple different DNA damaging reagents.

For Figure 4 - the inserts of non-myotis bats needs to have the same scale as the myotis bats (with numbers included, just like myotis bats) and is there a reason for including all the non-significant data? A standard P-value would be 0.05 and so this number is the most important. At this P-value there is 1 more VIP for RNA compared to DNA (51 vs 50), though yes the ratio is different. To me, the P-value of 0.05 is the only thing worth considering - if not justify how non-significant data is worth including/discussing ?? There is nothing in the methods about defining what is a DNA-only VIP vs a RNA-only VIP. how was this decided from the original VIP dataset (which is ~25% of all genes anyway at >5000). What about RNA/DNA binding VIPs since many viral pathways/sensor overlap depending on the virus? Ref. 109 has 1300 proteins - you should maybe mention the total of genes and the total number of orthologs (matched to the ref paper) as this is misleading. That paper is also quite old now and many VIPs have been further characterized since then - particularly with regards to binding both DNA/RNA instead of just RNA-only etc. It's not possible to really evaluate this figure without that information. How many of the VIPs are viral-specific may indeed be more interesting - once subtracting the DNA damage genes etc from the VIP list (as many moonlight and would be present in the VIP list).

Fig 5A/B/C is interesting but how it correlates to the whole story is not clear. it could easily be placed in the supplemental. There's no link. Fig S5I-J is interesting and fits - though again there's no control for bias based on evolutionary branch length / representation over and above expected evolution etc. The gene duplications etc do not match any virus pathway - but do match this VIP database - explain the inconsistency. What proportion of VIPs sit outside the single-copy ortholog collection (are they normally duplicated anyway - even if duplication etc is an antiviral strategy). evolutionary time-bias is therefore an important consideration also. longer time = more duplications, regardless of species. Please explain how clearly how this is controlled for.
There is very little linking immunity or viral infection with Figure 5 at all.

Figure 6D must be quantified to show the amount of pull-down (though yes, it seems clear from the image). Figure 6E is interesting but the conclusion it is additive cannot be drawn from the experiment.
While it does not appear synergistic or dominant negative the authors would need to perform experiments with 50% PKR1 + 50% EV and see the functional capacity of 350ng PKR1 compared to 700ng of PKR1 to see if the effect in PKR1/2 mixes are due to solely PKR1 or truly PKR2 + PKR1. The amount o DNAis ambiguous also as each experiment in E/F/G uses a different amount of DNA. The viability assay is good using small amounts (and is clearly not additive for viability at 100ng). The level of translation shutdown in E does not match viability in G (for 200ng). Its interesting to note that strong viability effect just from low-dose transfection alone, in the absence of polyIC treatment. The subtleties of this should be more carefully explained. For Figure F, the addition of PKR1/2 (at high doses) in the presence of an activating stimulus (dsRNA from VSV) - may be suggestive of an interaction not seen at the basal state. The results from S6C show it is not black and white 'additive' and clearly not as simple as the authors make it sound. For example the protein shutdown effect is known to block VSV but nfkb is also pro-viral for VSV (and IFN strongly antiviral) - so the 'tradeoff' has to be carefully explained.

It would be easy to test in the presence or absence of polyIC dsRNA to induce dimerization/activation or in the presence of a K271/K296R point mutation that would disable eIF2a phosphorylation but still maintain PKR's scaffold-pro NFkB role. How RNA virus induction correlates to the title of the manuscript is unclear. (except the already published VACV data).

Consistently throughout each section there are differences in the source data used including different number of genomes, different number of non-myotis bats, different number of other animal hosts. Its quite confusing to follow carefully and the greater impact of how the missing data may impact one single analysis is uncertain. As there is no subsampling, downmixing etc it is very hard to say how the numbers of each bracnh may bias the results.

In terms of the conclusions - while indeed wing punch - cell lines, genomes etc is a powerful approach - the authors

identified an RNA sensor (an RNA-only VIP) that has a strong effect in myotis. This is in contrast to the claims throughout the paper that DNA-only VIPs are more important in myotis. While PKR is important during DNA virus infections it is also important in RNA virus infections and not DNA-only. Other examples may have been much better for functional data, particularly considering the importance of myotis PKR in DNA virus infection was already published by the team. The use of PKR with RNA virus infections, while interesting to me, does not particularly add anything to justify the claims from the bioinformatics.

(Remarks on code availability)

Referee #4

(Remarks to the Author)
In their manuscript entitled: “Extensive longevity and DNA virus-driven adaptation in nearctic Myotis bats” Vazquez and colleagues investigate the evolution of extended longevity and infectious disease resistance. The study includes several major findings including the demonstration that virus adaptation in bats is mostly driven by DNA viruses. In addition, it provides chromosome-scale genome assemblies for eight new species of bats, an important resource for future projects on these potential reservoirs of zoonoses.
This paper addresses several fundamental questions regarding the evolution of extended longevity in bats with a powerful combination of new data sets. I enjoyed reading it but I think that the paper often tries to bring together some analyses, approaches or questions that are difficult to combine at the current level of understanding of this system.
For example, I was much less convinced by the section of the manuscript investigating the evolution of cancer resistance and I feel that this aspect of the study weakens this paper, in comparison to the findings and section on virus adaptation. First, I find the section using cell cultures superficial compared to recent papers using this approach in several vertebrate species (Firsanov, D., Zacher, M., Tian, X., Zhao, Y., George, J. C., Sformo, T. L., ... & Gorbunova, V. (2023). DNA repair and anti-cancer mechanisms in the longest-living mammal: the bowhead whale. BioRxiv), including a very recent paper in Myotis bats (Hua, R., Ma, Y. S., Yang, L., Hao, J. J., Hua, Q. Y., Shi, L. Y., ... & Liu, Z. (2024). Experimental evidence for cancer resistance in a bat species. Nature Communications, 15(1), 1401.). Establishing these cell cultures did not bring any clear results or identification of a mechanism of cancer resistance in myotis in the current paper. In addition, establishing cell culture from wild organisms is now quite common and should not be presented has a new tool or innovative component of this paper. Secondly, while it’s often assumed that bats are cancer resistant (see, in the current manuscript Line 479: Intriguingly, these species demonstrate some of the lowest known rates of cancer among mammals or Line 427: Bats are widely known for their long lifespan, cancer resistance, and viral tolerance), I think it’s simply not true and not based on actual epidemiological data. Recent large scale comparative analyses using necropy data show that cancer mortality rate or cancer prevalence vary a lot across bat species (Vincze, O., Colchero, F., Lemaître, J. F., Conde, D. A., Pavard, S., Bieuville, M., ... & Giraudeau, M. (2022). Cancer risk across mammals. Nature, 601(7892), 263-267. and Compton et al. (2024). Cancer across vertebrates. Cancer Discovery), with some bat species showing quite high cancer rates compared to other vertebrates. Finally, while I know that the Relative Intrinsic Cancer risk is a metric that has been used in other studies (mostly, but not only, in papers with some co-authors of this paper), I am still not completely convinced that this variable truly reflects cancer susceptibility across species (but I see that it can be used to just identify species under strong selection for the evolution of cancer resistance mechanism(s)). First, it assumes that a certain number of consecutive changes in the genetic material are needed to transform a normal cell into a cancer cell and that “Within species, lifetime cancer risk scales linearly with body size, and with lifespan by a power-law of exponent 6” (Line 176-177). I feel that we do not have evidence that this is true across species. On the contrary, we already know that naked mole rat or bowhead whale cell lines for example need different number of mutations for malignant transformation. So, can the authors explain the rationale behind the use of this variable across species and how it fits with the recent work of Cagan et al. (Cagan, A., Baez-Ortega, A., Brzozowska, N., Abascal, F., Coorens, T. H., Sanders, M. A., ... & Martincorena, I. (2022). Somatic mutation rates scale with lifespan across mammals. Nature, 604(7906), 517-524.)? Second, the metric does not include real cancer prevalence data that are now available for hundreds of vertebrate species. In other words, two species of similar size and lifespan will obtain the same Relative Intrinsic Cancer risk score but based on epidemiological data published, we know that these two species might show really different cancer prevalence. A real and very useful tool to detect species that have evolved cancer resistance mechanism would instead combine the Relative Intrinsic Cancer risk variable used in this study with real epidemiological data on cancer prevalence.

Other comments:

Abstract: I found the abstract a bit weak, the last sentence, for example, is not useful and does not reflect the impact that this paper can have on this topic and the scientific community.

L179: “The observation of similar lifetime cancer incidence rates across mammals”. I disagree with this sentence, mammalian species have very different lifetime cancer incidence, it has now been shown several times.
L188-196: I think that the authors should provide some real statistics to support this. i.e. Myotis show some of the most pronounced decreases in RICR among mammals.
L204: three pathways are mentioned in the previous sentence (not two).
L231: Why 11 species? Which species?
L244-260: Why would you see a decrease of viability and an increase of apoptosis in response to a radiomimetic agent in a species with an increase ability for DNA repair. I find it counterintuitive.

(Remarks on code availability)

Version 1:

Reviewer comments:

Referee #1

(Remarks to the Author)
The authors have done an excellent job in revising the manuscript, which has been substantially improved; many of the earlier issues have been corrected or further clarified. The inclusion of the new RNA-seq data provides valuable additional insight into the DNA damage response in Myotis cells. The section on intrinsic cancer risk is now clearly articulated, and the experimental results are generally more interpretable. The observation of the trans-species heterozygous PKR duplication is an impressive demonstration of the potential of haplotypic resolved genomes.

I am fully supportive of the publication of this paper, provided that the authors address the following comments, some of which pertain to earlier remarks.

- LPP scores from ASTRAL-IV are not displayed in Figure 1. At a minimum, it would be helpful to indicate nodes with low support, e.g. the root of Nearctic Myotis. The statement in line 103, “Using these annotations, we resolved the conflicting phylogenies of Nearctic Myotis,” should perhaps be toned down, as the low root support suggests the phylogeny remains only partially resolved.

- The reported average of 27,536 protein-coding genes appears to be substantially inflated. Mammals, including bats, typically have around 20,000 genes. This suggests that the annotation likely contains fragmented or erroneous genes. The high figure is particularly striking given that the BLAT approach described in the methods added approximately 1,800 further genes.

- In the previous version, line 138 read:
“To resolve the phylogeny of Nearctic Myotis, we identified single-copy orthologs of 17,509 protein genes present in 536 mammalian genomes,”
whereas the revised version states:
“A phylogeny of all 534 mammals in our alignments,”
and the number of genes changed to “15,734 gene alignments spanning 534 mammals.”
Since the methods description appears unchanged, the authors should clarify which two species were removed and the rationale for the reduction from 17,509 to 15,734 genes.

- The authors state that “The table including the list of species and orthologous gene alignments are now included in Table S2.”
Table S2 does indeed contain the list of species, but not the gene alignments, which would be a useful resource for future studies.

- Adaptation to RNA viruses appears to be primarily driven by changes in gene copy number, as shown in this study. I therefore find the statement in the abstract—“but not RNA viruses”—somewhat misleading, and would suggest instead highlighting the distinct modes by which DNA and RNA virus-interacting proteins evolve.

- Figure 4 contains minor visualization issues: some panels appear shifted upwards, likely due to the omission of panels J–L.

- Line 116: the 20-million-year estimate conflicts with the 33-million-year figure reported in line 63.

- Line 172: it should be clarified that PKR1 + PKR2 co-expression refers to a half-dose of each gene. It would also be useful to note that, despite PKR1 being expressed at lower levels, it consistently proves the more potent of the two duplicates in all experiments.

- Line 293: consider rephrasing as “Genes under positive selection were enriched,” given that the preceding sentence refers to negative selection.

- The abbreviation “NCS” should be defined at line 314.

- Line 362: the authors likely refer to Yangochiroptera.

- There is also a minor typographical error in line 178: “e recently duplicated PKR1s homodimerized.”

(Remarks on code availability)

Referee #2

(Remarks to the Author)
The authors have improved the manuscript by restructuring the text and refining/toning down the language to more accurately reflect the scope and nature of their findings. The presentation is now clearer and more cohesive. The addition of the RNA-seq analysis provides further support for their conclusions, particularly by linking DNA damage responses to immune pathways, and represents a valuable enhancement to the study. The PKR analysis is interesting in the sense that the availability of complete genome sequences allow the authors to conduct evolutionary analyses that clarify the history and structure of this known duplication. However, I do not believe the functional analyses presented in this section offer substantial new mechanistic insights beyond what was previously reported.

I commend the authors for the thoughtful and constructive revisions they have made in response to the reviews. The science is rigorous, and the authors leverage high-quality, haplotype-resolved genomes to generate well-supported insights within a strong statistical framework. Nonetheless, I maintain my original assessment that the main findings largely reinforce existing knowledge about the evolution of key traits in this system. In my view, the degree of conceptual novelty and the extent to which the work advances the field are more modest. It may therefore be appropriate for the Editors to consider whether the manuscript is best suited for a broad, general-interest journal or a more specialized venue.

(Remarks on code availability)

Referee #3

(Remarks to the Author)
The authors have modified the flow and arrangement of the manuscript considerably since the first submission and here they present data on longevity and virus-driven evolution in myotis bats. The abstract and introduction read much better and have a lot of clarification. The data on the additional myotis genomes are presented clearly with statistics etc across figures 1 and 2. There is little effort to correlate their findings to the literature in terms of all the other myotis genome publications including the one last year (https://academic.oup.com/mbe/article/41/9/msae165/7730189?login=false#). Figure 4A is nice however its really odd that some of these bats (like auriculus) are not presented later on in Figure 5 D. Figure 5D also adds griescens/brandtii/myotis etc and so it would be better if these were also included in Figure 1A with their longevity quotient. The PKR section kind of comes out of the blue now, without any kind of link or lead in (1 sentence in the introduction). The data itself is clear and with a more logical interpretation etc. There are some slight differences, pkr2 maybe less functional to both virus and cell translation etc. The wording for Figure 4 and description of each VIP set is much clearer and easier to follow however the separation highlights what I find are some large inconsistencies in the presented data and the conclusions drawn.
In other mammals there are clearly more RNA-only VIPs under selection then DNA-only VIPs (and not the case in myotis or barely in non-myotis bats). However DNA-only VIPs are still very strongly under selection in other mammals.When looking at just truly "significant, P=0.05" data the ratio of VIP:control is 50 vs 51 - showing no difference in # of DNA-only vs RNA-only in myotis bats. The ratio is important and at 3 vs 1 there is more DNA then RNA yes. These figures look really poor and maybs its the PDF generation problem but it seems to be badly photoshopped. Ensure the scale is on each figure (d, e, f etc not just on the left side of D). In non-myotis bats the ratio is ~4 to ~ 1.5 - so about the same. Meaning there is no difference between myotis or non-myotis bats in this regards. The ratio of RNA-only VIPs is very similar to all other mammals (between 1-2) and so the only thing you can claim is that - unlike other mammals, it is much more likely that DNA-only VIPs are under positive selection in (all) bats, regardless of their longevity quotient/status etc. It's also strange that the number of genes is so different yet the ratio (VP:control) is not, when you are dealing with only commonly identified orthologs across the board (in the VIP sets). Overall, the conclusion that's bats have a stronger adaptation to DNA-only VIPs is valid - but it seems to be unrelated to longevity, myotis or geographic region. The Fig 4 figure legend is inaccurate.
DNA-only VIPs are strongly associated with cell cycle, DNA repair etc etc (fig. 6 C-F) and so to claim viral evolution / arms race as a driver of their evolution is very risky unless you show the exact same genes in the VIP set are under selection and the same genes in NCS-treatment etc. Also you would have match the evolutionary time of these genes (tmrca) with the age of the DNA pathogens found in bats, to really claim this. So be cautious with the wording.
For teh RICR there are some conflicting data that are complete glossed over. Yumanensis has quite a higher end RICR yet a lower longevity quotient - why does it sit up close to myotis myotis? Also auricalis is missing and some others like thysanodes should be much higher shouldn't it? It would be good to see all the myotis highlighted here and consistently use all the myotis genomes you added. How to explain the inconsistencies? particularly when the RICR score is used to justify myotis having selected genes for longevity. Similarly, thysanodes/lucifigus etc show more cancer pathway genes (5E) yet volans etc do not? so is the % of cancer genes are valid point to make? I like the raincloud plot in the supplemental but it sees these strongly-selected genes are somewhat different to the NCS/treatment data.

in the summary you link DNA virus adaptations to cancer resistance etc (yet there was no real difference in DNA virus evolution between myotis vs non-myotis bats, yet you still claim there is a difference in RICR for myotis, specifically ?) . These two points do not align. the comment on DNA transposons needs to be clarified due to the differences in different transposons between bats.
Overall the data itself is pretty clear and presented accurately but some of the conclusions I feel are strecthed to far from the data actual represents. This paper has generated some beautiful genomes and there is clearly a difference in the pressure of which genes to evolve (e.g. DNA sensing.repair machinery) - but this was kind of already known for bats and it is not a myotis-specific trend that has been pulled out. The PKR section is interesting though barely mentioned in the discussion.

Gene duplication is an interesting trend that needs further observation. PKR itself is incredibly important for cell stress, stress granule formation, control of metabolism, scaffolding for NFKB, inflammasome activation and sterile inflammation (independent of any virus) - any of these pressures may have triggered gene duplication and none of this is mentioned.

(Remarks on code availability)
The usage of ASBRel, cafe, busted etc seems quite normal. assembly and annotation seems quite logical and similar to methods I have used before.

Referee #4

(Remarks to the Author)
I am reviewing this paper for the second time. I think this new version of the manuscript present some very solid results in addition to publish the sequencing of 8 new bat genomes (an important resource for new studies). So, this paper is definitely of high quality and should be published in a high-ranking journal. However, I still think that the approaches used and results are not novel enough for publishing in a journal such as Nature. I feel that this paper is a broad examination of the very interesting biology and genetic of Myotis, opening a lot of research avenues for future papers and studies, but in-depth examination of the mechanisms at play is essential, in my point of view, to publish in Nature. For example, while the results obtained using the primary cell cultures are interesting, many more experiments are now needed to understand the characteristics of the DNA damage response in these species and how it might confer an increased resistance to cancer or not. This aspect is also not put into perspective in the discussion.

See minor comments below.
Abstract: I still think that the last sentence of the abstract is really weak and this papers should have a much more important conclusion/perspective than what is stated in this last sentence.
A third PKR haplotype - H3 - with three tandem duplicates of PKR (PKR1, PKR2, and a third copy) present only in M. californicus have been detected in this study. Then when the functional impact of the duplicates' co-expression has been investigated, only PKR1 and PKR2 have been studied. Can the authors explain why this third copy has not been further studied?
The rationale for studying PKR more than another gene is not super clear to me.
L260-261 : This is the theory and it has to clearly stated. Maybe, within species evidence should be cited ?
L277-279: "This is consistent with recent pan-mammal and pan-vertebrate studies of cancer risk, which found that bats were present among the 20 species with the lowest rates of neoplasia, and not at all among the 20 species with the highest rates of neoplasia.". I still disagree with this statement. I still think that the data published so far are showing that the prevalence of cancer in bats is extremely variable between species. The fact that none of the bat species is among the 20 species with the highest prevalence of the study does not mean that bats can be considered as cancer-resistant.
The first paragraph of the discussion is trying to convince the readers that collecting wing punches to establish primary fibroblast cell cultures is a revolutionary approach but I am clearly not convinced. This is a basic methodology in this field!
L364-365: "We find that Myotis demonstrates an increased rate of change in lifespan given body size compared to other mammals." This is actually not true given that "We find that while non-bat mammals experience an average 0.159% increase in lifespan per 1% increase in body size, bats experience a 0.223% increase in lifespan years per 1% increase in body size, though these rates were not significantly different after phylogenetic correction" L254-255.

(Remarks on code availability)

Version 2:

Reviewer comments:

Referee #3

(Remarks to the Author)
The authors have gone a long-way towards clarifying a lot of the subtle nuances in the topic and addressed most of my concerns. They clarified a lot of the points that required further details/expansion.
One final note, just because a gene has proviral/antiviral function does not mean that its reason for evolving is due to a virus-host arms race. If you are comparing a gene that is 20 million years old and saying the evolutionary pressure was due virus then you must test it has antiviral/proviral function on one of those 20-million old pathogens... and even then, it still doesn't mean that's the cause when they are, as the authors detail, pleiotropic. A nice job in the revisions though.

(Remarks on code availability)

Referee #4

(Remarks to the Author)
I appreciated the authors' decision to moderate their claims regarding the fact that bats would generally be cancer resistant,

notably through the removal of the RICR analyses. However, beyond this point, the revised manuscript appears to contain only relatively minor clarifications. Consequently, this new version still does not, in my view, provide substantial improvements over the original submission.
I remain concerned that:
(1) the functional analyses presented remain too preliminary to support publication in a journal of this scope and visibility;
(2) as previously noted both by another reviewer and myself, the degree of conceptual and experimental novelty relative to the existing literature appears insufficient to justify publication in Nature;
(3) the establishment of primary cell cultures from skin biopsies is already a widely used methodology in biology — even if perhaps less common in bats — and although I acknowledge that genomes derived from primary cell lines may offer advantages over those generated directly from tissues, I do not consider this to represent a sufficiently major methodological advance for publication in such a journal;
(4) the authors overstate their conclusions when claiming that they "demonstrate a unique DNA damage response in primary cells of the long-lived M. lucifugus." The experiments presented here do not, in my opinion, support such a strong statement, and a substantially more detailed functional characterization of the genes and pathways involved would be necessary to support this conclusion;
(5) the introduction remains insufficiently developed with respect to the current state of the art across the different aspects addressed in the study. In its present form, it remains overly simplistic and does not establish a sufficiently rigorous hypothesis-driven framework, which I believe is essential for a manuscript intended for publication in a high-impact generalist journal.
That being said, I believe that this study represents a substantial and impressive body of work, is conducted with scientific rigor, and provides a valuable foundation for future investigations in this field. In my view, the manuscript would therefore be well suited for publication in a more specialized journal, such as Nature Communications, where its methodological and comparative contributions would likely be of considerable interest to the relevant research community.

(Remarks on code availability)

Open Access This Peer Review File is licensed under a Creative Commons Attribution 4.0 International License, which permits use, sharing, adaptation, distribution and reproduction in any medium or format, as long as you give appropriate credit to the original author(s) and the source, provide a link to the Creative Commons license, and indicate if changes were made.
In cases where reviewers are anonymous, credit should be given to 'Anonymous Referee' and the source.
The images or other third party material in this Peer Review File are included in the article's Creative Commons license, unless indicated otherwise in a credit line to the material. If material is not included in the article's Creative Commons license and your intended use is not permitted by statutory regulation or exceeds the permitted use, you will need to obtain permission directly from the copyright holder.
To view a copy of this license, visit https://creativecommons.org/licenses/by/4.0/

We thank the reviewers for their constructive feedback. We were particularly excited to hear that they enjoyed reading the "*comprehensive set of computational analyses*" based on the "*high quality of the genome sequences (haplotype-resolved, chromosome-scale assemblies) assembled*;" and that they believed that "*this paper addresses several fundamental questions regarding the evolution of extended longevity in bats with a powerful combination of new data sets*."

However, the reviewers also correctly noted that there was much room for refinement and improvement. In particular, we found that several of the comments raised by the reviewers arose as a consequence of the way in which we presented the results. We have thus extensively restructured the manuscript to convey both the novel findings empowered by our near-complete Myotis genomes as well as the narrative of agonistic pleiotropy between antiviral immunity and longevity-associated traits like DNA damage repair. Additionally, we have generated a new RNA-seq dataset based on our results and the suggestions of the reviewers, and uncovered new evidence to strengthen our hypothesis of strong agonistic pleiotropy between anti-viral and DNA damage response in bats.

As such, the new flow of our manuscript is as follows; please note that we indicate in parentheses where the original figure moved:

- Overview of the high quality genome assemblies and samples collected: Figure 1 (prev: Figure 1)
- Karyotypic and structural evolution among genomes: Figure 2 (new,subfigures from Fig. 3 and supplements)
- Characterization of the sequence, genetic structure, and function of the duplication of the antiviral factor PKR: Figure 3 (prev: Figure 6)
- Unique selective signatures in viral interacting proteins among *Myotis* genomes and bats overall: Figure 4 (prev: Figure 4)
- Peto's Paradox and evolution of cancer resistance: Figure 5 (prev: Figures 2 and 3)
- Pleiotropy between DNA damage response and innate immunity in *Myotis lucifugus*: Figure 6 (new)

In addition to the new structure, we have moved extraneous details to our Extended Data and Methods section in order to streamline our narrative. Additionally, we have now archived all additional raw data into a new Dryad repository associated with this manuscript: http://datadryad.org/share/VLwqtJ4gv1joM8_0JaZen4y7Un2TjGcudZXluzjbpLQ.

Overall, we believe that the combination of editorial changes and additional analyses has greatly improved this paper, and we present it once more for consideration in *Nature*.

Referee #1 (Remarks to the Author):

This study focuses on the genus Myotis, a group of bats known for their large variation in lifespan and adaptations to viruses. The authors generated cell lines and high quality genome assemblies for eight Myotis species. They performed extensive analyses that

provided evidence of positive selection linked to longevity, cancer resistance, and viral interactions. The findings regarding adaptations to DNA but not RNA viruses are very interesting. Functional cell experiments were also conducted, although I have questions about both experiments.

Overall, this manuscript provides important new resources and a number of interesting results.
I have several questions and need clarifications on the analyses and the experiments.

- l118: Please define auNG as this is a not widely used measure (so far).

We have added a note in the Figure 1 legend to explain that auNG represents the "Area Under the NGx curve", and is a metric of genome contiguity independent of genome size. We selected this metric as opposed to others such as NG50 as it provides a comparable point measurement of genome quality across the full range of genome sizes in mammals.

- l143: 100% bootstrap support is typically observed with large datasets. To support the correctness of the phylogenetic tree inferred with IQtree and concatenated alignments, coalescence methods should be used. Node support can be measured as the proportion of gene trees that support the species tree.

We thank the reviewer for their suggestion. We used the latest version of ASTRAL (ASTRAL-IV) to score our maximum-likelihood species tree, and observed posterior probability supports of 100% across nearly the entire phylogeny, with a handful of exceptions; this is not unexpected given the original Sayyari & Mirarab (2016) paper describing the local posterior probability metric using quartet scores. Notably, the root of Nearctic *Myotis* (*M. brandtii* as outgroup to Nearctic *Myotis*) has extremely low quartet support (~36%), which has been previously observed in other bat phylogenetic studies. Given the geographical boundary between the most closely related species (*M. volans* and other Nearctic *Myotis* are in North America, while *M. brandtii* is exclusively European), we are confident in our placement of *M. brandtii* as the outgroup to Nearctic *Myotis*, as has been agreed upon by others in the literature (e.g. Foley *et al.* (*Cell Genomics* 2024) and Jebb *et al*. (*Nature* 2020)). We chose to use the LPP score from ASTRAL-IV rather than the proportion of gene trees since, as observed by Foley *et al*. (*Cell Genomics,* 2024) and others, there appears to be rampant introgression across *Myotis*, and our initial analyses of the population genetics of these species supports this conclusion.

- l170: Are myotis species statistical outliers in this analysis?

We found that our PGLS and PANCOVA analyses were underpowered for *Myotis* due to the small number of species included in the Upham *et al*. (2019) phylogeny with both accurate body size and lifespan estimates (n=21). As such, while we do highlight the clade in the prior Figure S4A-B, we did not treat *Myotis* separately from all bats in this statistical analysis. To clarify the conclusion of this section in the text, we have

edited the concluding sentence in the paragraph to read: “ Together these results demonstrate that *Myotis* bats and their recent ancestors have evolved some of the most extreme increases in lifespan among mammals, despite similar allometric lifespan scaling of bats and mammals after phylogenetic correction.”

- Reduced Intrinsic Cancer Risk per cell.
From the DT^6 equation in the methods, it is clear that if D=body size stays the same and T=lifespan doubles, the risk increases log2(2^6) = 6fold. It is good to quantify this, but given this equation and what we know about Myotis species, I find it a rather trivial result that some myotis species have a large RICR variation.

While we agree that it is unsurprising that Myotis, as long-lived species, have large shifts in RICR, the novelty of our findings is the resolution to answer *when* and *where* this occurred on the phylogeny. Our ancestral trait reconstruction of lifespan enables us to model how RICR changes in evolutionary time. Our results suggest that cancer risk has generally gone up throughout the evolutionary history of *Myotis*, with the greatest shifts in RICR having occurred at the most recent common ancestor between extant sister species. This is in contrast to other possible modes of evolution, such as a long-lived common ancestor to all *Myotis* or bats with little modern change, or a short-lived common ancestor with 100% of the shift in RICR occurring at the tips. These results inform our hypotheses of selection on genes associated with cancer.

Moreover, I find the double usage of "reduced" difficult to understand in sentences like "Decreases in (Reduced Intrinsic Cancer Risk) correspond to an increase in the expected cancer risk" or "decreases in RICR" or "bottom 10% of RICR". I would suggest to speak of the ICR, as we all understand what it means if ICR increases or decreases, or if ICR is high or low.

We agree. The original language was used for consistency with the original RICR publication (Vazquez & Lynch, 2021). However, the term *Relative* Intrinsic Cancer Risk is clearer. We have changed the manuscript to use this term and clarified the language overall to make these analyses easier to understand.

- l200: On average 22.7% of genes were under selection across the 9 nearctic Myotis species. If this analysis also considered internal and not just the terminal branches, what does the average refer to? Moreover, 22.7% is a rather high percentage for a group of nine related species.

We have edited our text to clarify that an average of 5.46% of protein-coding genes had at least one region under positive selection per node at FDR <=0.05; in contrast, an average of 20.8% of protein-coding genes per node at FDR <=0.05 had all regions with an omega less than 1, suggesting negative selection. Our selected method, aBSREL, incorporates controls for expected dN/dS for each sequence using the species phylogeny.

- l200: The methods describe that 70 primates, 138 euungulates, 127 glires, 82 carnivora and 47 pan-chiroptera species were included. This sums to 464 but not 536 species. Please clarify.
Several important datasets including the list of these 536 or 464 species and the orthologous gene alignments are also missing.

Orders with few species represented in the dataset were excluded from our VIP analysis, leaving only the four additional non-Chiropteran orders described with the stated number of species. We have refined the text throughout the manuscript to remove this confusion. The table including the list of species and orthologous gene alignments are now included in Table S2.

- l202 and l1624 and Fig 3A: Since aBSREL fits up to three omega values to model codon evolution. I suppose what is meant with omega > 1 in this line is not the overall genewise omega but one of the three omega values? It would then be important to also specify the proportion of sites that evolve with the omaga > 1. This should be clarified in the text as well as the figure, maybe by plotting the proportion as a color code.

We thank the reviewer for this point - for simplicity, we defined a gene as under positive selection if, after filtering for FDR<=0.05, at least one of the up-to-three significant omegas was greater than one; in contrast, a gene was defined as being under negative selection if all omega values were below one. We have added a sentence in the results section to clarify this.

- l208: How are cancer-related pathways defined? Looking at table S3, I can see for some Myotis species gene sets labeled as cell cycle or other relevant terms, but it is not obvious that the enrichments shown are strongly related to cancer. I also wonder why gene ontology terms were not tested?

All 2598 Reactome pathways used by WebGestalt (https://www.webgestalt.org/) and their classifications as either cancer or not-cancer are listed in Supplementary Table S7, in the tab "Reactome_cancer". We opted to focus on pathway-level overrepresentation analyses, as opposed to gene ontology overrepresentation, since we wanted to test the broader pathways that were under selection across the phylogeny as opposed to the biological functions of specific genes.

- l218: Table S3 shows ferroptosis as a word to occur in the first sheet, which is unclear as this sheet is listing the genes and not the enriched gene sets. The other sheets listing the enriched pathways per clade do not show ferroptosis.

The analysis of selection within ferroptosis-related pathways was a specific observation we made; "Ferroptosis" is not a pathway in Reactome. We include a figure in the extended data from KEGG (which does include a "Ferroptosis" ontology) highlighting the ferroptosis results, and have moved the related text on ferroptosis from the results to the extended data.

Moreover, the organization of the supplemental tables needs to be improved, including clearly labeling columns, stating where to find what data and what the different sheets

mean. This also applies to other tables. For example, what is RERC PC2 ORA in Table S4?

We apologize for the unclear organization of the supplemental tables. We have added README tabs to tables like Table S7 (ABSREL results) where additional clarity was needed.

- l255: Figure 3D shows for apoptosis and myotix lucifugus four outlier values with a fold change of 5-6 while the other data points are around a fold change of 2. The confidence intervals do not reflect these 2-3 fold increase for some of the replicates. There is no table with the raw values, therefore I could not check if this is a plotting error. If this is not a plotting error what explains that this large deviation that was only observed for M. lucifugus. Important, would removing these outliers render the difference to other species not significant?

(Note: the original Fig 3 is now Fig 4)

We apologize for the organization of the tables; both the raw data and the normalized values were located on the first two tabs of Table S7 in our original submission. The outlier for *M. lucifugus* comes from an individual that has a 6-fold response to NCS, and was replicated multiple times; this individual is heterozygous for a duplication of TP53, which is the focus of a separate manuscript in progress. Excluding this individual from the analysis, we find that the results observed remain significant for both the drop in viability and increase in apoptosis.

- There are 5527 VIPs listed in the supplemental table. This is 25% of all genes. If these proteins truly physically interact with viruses as stated in the manuscript, then many of these proteins likely have no effect on the virus and binding is coincidental or biologically not relevant. This is should be made clearer in the manuscript.

We have clarified in the manuscript that the VIP dataset is an expansion of the dataset reported in Souilmi et al. *2021*, which had 5,291 VIPs.

These VIPs were identified in the literature from low-throughput molecular methods and diverse high-throughput mass-spectrometry studies. Of these genes, at least 2,329 have well-studied, well-characterized anti- or pro-viral impacts and analyses of these genes alone recapitulate our findings (**Figure 5B,C**; **Table S8**). An example of this standard is CD45, which we highlight and describe in Fig. 5A. We note that any inclusion of genes with incidental viral interactions would make the results presented more conservative.

- l339: If DNA transposable elements catalyze large-scale rearrangements, why would they be depleted at the inversion boundaries? I also struggle to see the depletion in figure 5B, which does not show the narrower windows of inversion boundaries. Rather, what seems to be obvious is that regions with more segmental duplications overlap LINE peaks.

(Note: Fig 5 is now Fig 2)

We have repeated our analyses and modified the figure to examine the relationship between structural variation and mobile elements more rigorously. These results highlight strong

correlation between segmental duplications and DNA TEs ($R2=0.15$) and LINE elements ($R2=0.49$). We have revised the text to focus on these signatures (Extended Data Fig 3).

- l345: How are the gene families for the CAFE analysis defined? This is not described in the methods.

These analyses refer to the copy number of human-orthogous genes. We have modified the text to make this clear and removed mention of "gene families."

- l366: The FBXO31 expansion may be an error. Ensembl orthologs list many additional copies of this gene in Myotis lucifugus, but they seem all be intronless retrogenes. These genes may have a function though (https://www.nature.com/articles/s41420-023-01348-7). However, it should be carefully assessed whether the reported copies in other Myotis species are segmental duplications or retrogenes and, if the latter, whether the retrogenes are transcribed and translated.

While 24 copies of FBXO31 in *M. lucifugus* are mono-exonic (and thus likely retrogenes), 8 of them preserve an intron-exon structure. Both types of copies are spread across the genome in all *Myotis*. The functionality of these copies are the current focus of a separate manuscript; in the interest of conciseness, we have opted to remove the discussion of FBXO31 from this manuscript aside from the copy number featured in Figure S5A.

- l413: I fail to see the additive effect in Figure 6E. PKR1+2 has a less efficient or similar translation shutdown compared to PKR1 alone. Additive effects should result in a stronger shutdown for PKR1+2, which is not visible in the figure.

We agree that our definition of "additive" was unclear, and we have now clarified our experimental setup in both the Methods and Results sections. We used the same amount of total DNA in all conditions so that PKR1, PKR2, and PKR1+2 all have the same total mass of transfected DNA (which also matches the empty vector control). As such, the observation that the effect of a half-dose each of PKR1 and PKR2 in cells is the average of the two full-dose experiments with PKR1 and PKR2, and agrees with an additive model. We have edited the text to clarify this point.

- l408: I am not convinced that HeLa cells are an appropriate system for these experiments, also because the authors fail to find a strong effect. These experiments should be conducted in the Myotis cell lines that the authors have generated.

Our goal in these experiments was to validate the functional activity of a two-copy system of PKR. As the original duplication predates all Nearctic *Myotis*, any experiments done in these cells would be in the context of higher-order interactions with PKR1/2, and of the greater innate immune response of *Myotis*. We selected a full-PKR knockout cell line as a cleaner model for testing the direct effects of PKR1/2 when co-expressed, and stand by our results. Using model cell lines such as HeLa and HEK293 that are KO for genes of interest is a standard way to model the functions of specific genes in bats without the interference of other adaptations (see e.g. Morales *et al*. *Nature* 2025 with ISG15).

- l418-422: The interpretation that high doses of PKR led to cell toxicity would only be relevant for Myotis species if they express PKR1 and PKR2 together at levels similar to those in the overexpression assays. To reach a relevant conclusion, one needs to compare the expression levels in the cell assay to those in Myotis tissues.

We have previously shown that both PKRs are expressed in different tissue types in *Myotis velifer* under both +/- IFN stimulation conditions (Jacquet *et al*. 2022). PKR1 has a lower basal expression level, which we hypothesize is a cell toxicity tradeoff. We agree that protein-level expression would be ideal, however, none of the antibodies against PKR that we tested are cross-reactive with *Myotis* PKRs. Therefore, while we have previously shown expression at the mRNA level of both copies, we were not able to quantify this at the protein level.

- l1641: I assume the 19,646 Myotis orthologous CDS alignments and the 17,469 non-Myotis bat alignments contain distinct species, as otherwise the signal from shared species would be considered in both datasets. This should be stated in the methods.

We thank the reviewer for highlighting this point. The 19,646 *Myotis* orthologous CDS alignments contain only *Myotis* species, while the 17,469 non-*Myotis* bat alignments contain only non-*Myotis* bat species; thus, the species sets are mutually exclusive. We have clarified in the text: "The species in these two sets (*Myotis* bats and non-*Myotis* bats) are mutually exclusive. This includes a subset of 14,091 alignments with orthologs present in two thirds of the non-*Myotis* bat species, regardless of the genes' presence/absence in the *Myotis*-only complement. This is specifically used to show that patterns of virus-driven adaptation are representative of all, and not just a limited subset of bats."

Moreover, are the number of species in these CDS alignments matched?

The number of species in the CDS alignments for each gene is indeed an important confounding factor, and is one of the confounding factors matched between sets of VIPs and control genes using the pipeline, as described in Enard and Petrov 2020 and in the methods: "the number of species with a one to one ortholog out of all the species included in an alignment, where species with no ortholog are represented by gaps the whole length of the alignment."

- l1578: What are supplementary files XY ?

This line referred to a table containing all the accessions for genomes used in the multiple sequence alignment, which was mistakenly omitted. We have now included this table as Table S2.

- l1966 typo xo-expression
- figure S3 is very blurry and not readable
- typo in l156 Yinpterochrioptera

We thank the reviewer for bringing these issues to our attention; we have made the corresponding corrections to each item, and we have fixed the compression issue in the PDF that led to Figure S3 being blurry.

Referee #2 (Remarks to the Author):

The study by Vasquez et al presents a comprehensive set of computational analyses based on the sequencing of eight bat genomes from the genus Myotis. These genomes allow the authors to resolve the previously unclear phylogeny of Myotis. Additionally, the authors examine the relationship between lifespan and body size, finding evidence of rapid evolution toward increased lifespan. Similar to other vertebrates with extreme changes in body size and lifespan, the authors identify strong selection for cancer resistance mechanisms at multiple instances within Myotis. Through selection scans, the study finds associations with genes involved in cancer, DNA repair, immunity, and metabolic processes. The scans also suggest that DNA viruses have exerted stronger selective pressures than RNA viruses. Lastly, structural variation analyses reveal that gene duplications are concentrated in pathways related to cancer, aging, immunity, and olfaction.

The manuscript constitutes a valuable genomic resource due to the high quality of the genome sequences (haplotype-resolved, chromosome-scale assemblies) and the importance of bats as a model organism. While the high-quality genomes allow for more thorough comparative analyses than those previously performed in bats, the approaches used here are standard in comparative genomics. The main traits studied, such as cancer resistance and lifespan, have already been reported as displaying unique patterns in bats. Consequently, the results, while valuable and solid, are not particularly novel.

We agree with the reviewer that we should have done a better job in the introduction and elsewhere to highlight the novelty of our work compared to prior work. We have modified the corresponding sections of the manuscript to clarify these points.

While previous work has highlighted cancer resistance and extreme lifespan in bats, it is typically assumed that these adaptations happened at or near the root of bats. Our genomic and functional analyses suggest that the independent evolution of longevity in individual bats species is more widespread than previously assumed. Understanding *where* greater longevity and cancer resistance has evolved in the *Myotis* phylogeny allows for more powerful investigation of the genes and molecular innovations associated with these traits.

While there are common themes underpinning the evolution of longevity and cancer resistance, the path to extreme lifespan is distinct across taxa (see Li, Vazquez, and Sudmant 2023). Our analyses highlight lineage-specific patterns of adaptation associated with longevity and cancer resistance in *Myotis*; innovations associated with DNA but not RNA viral tolerance across bats that have never been described; and pleiotropic links between genes in these two processes. Together our study

demonstrates how T2T genome assemblies, primary cell lines, and functional follow-up can provide unique insights into a taxa with extreme lifespan and substantial contributions to zoonoses.

I find the introduction and overall tone of the paper to be misleading. It emphasizes bats as a unique model system because it is a group in which one can combine comparative and functional/mechanistic approaches, creating an expectation that this study will do so. Rather than focusing on a specific result for in depth functional exploration, as the narrative suggests, the manuscript presents a broad collection of analyses that identify general evolutionary patterns. The results from these analyses are then presented and correlated with various life history traits, without any attempt to delve deeper into the underlying mechanisms. Doing so would have been impactful, and would have provided significant insights into the biology of this group, beyond what is already known. Instead, the reader is left wondering whether the many genes/pathways identified have any real relevance to the traits being examined, and whether they go beyond the correlations presented.

On revision, we have made significant changes to the narrative throughout the paper to better reflect the scope of the work we present herein. We agree that our study represents a breadth, rather than depth, of vignettes that highlight the remarkable biology of *Myotis* and its value to multiple disciplines. We believe that we have presented sufficient evidence in each vignette to support their respective conclusions, and highlight the future directions opened by this manuscript for researchers across various disciplines.

There are two functional studies presented, one showing that M. lucifugus has evolved an enhanced DNA double-strand break response and the other one examining structural differences in the PKR locus. The first one, while interesting, confirms a pattern seen in the genomic data but does not offer any insight into mechanism. Moreover, the results presented in Fig 3D, particularly for viability, show that the differences are quite mild, leaving the reader wondering about the relevance of this observation.

We would like to highlight that there is a 2-fold change between *M. lucifugus* and the other bats for the activation of Caspase 3/5 (“Apoptosis”) at 500uM; however, in the literature, the LD50 for cells for NCS is significantly lower (5-20uM). We interpret our results as indicative of a general resistance to DNA damage in *Myotis* and bats (which has previously been shown), with the addition of an enhanced “fix or die” phenotype in *M. lucifugus* reminiscent of mechanisms identified in elephants but not naked mole rats - two poster-children for the evolution of longevity and cancer resistance.

To add to our mechanistic understanding of this process, we performed RNA-seq for multiple (n=2) individuals of *M. lucifugus* at two timepoints (6 and 18 hours). These results are now described in the new Figure 6 and Extended Data Fig 7 in our manuscript. These results show that 1) in response to DNA damage *M. lucificus* mounts an up-regulation of genes associated with cell cycle arrest (e.g. *MDM2, CDKN1A*) and cell death (e.g. *BAX*), as expected given our apoptosis and viability assays; 2) the strongest pathway enrichment in our DNA-damage RNA-seq data were genes

associated with the innate immune response, including genes in the PI3K/AKT pathway, providing a link between immunity and DNA damage in *Myotis* bats; 3) We find that genes up-regulated in response to DNA-damage are highly overenriched for the DNA-associated viral interacting proteins that we identify as under selection. Together, these results strengthen the conclusions of our manuscript.

The second one -examining structural differences in the PKR locus- is underwhelming for a couple of reasons: First, the bat-specific structural differences at PKR had already been identified in a previous study examining bat-viral interactions, so this is not a novel finding from this study. The current work examines the combined effects of PKR1 and PKR2, as opposed to independent effects, as examined in the other published study. I consider this addition incremental. This begs the question: Why didn't the authors carry out functional/mechanistic exploration of one of the many genomic correlates identified in this particular study, instead of using a structural variant that was already known? I think this is a missed opportunity to show that bats are indeed an ideal group to carry out complementary genomic and functional studies, as the authors argue.

We would like to clarify that while the duplication of PKR was previously known, its evolutionary history and structure were not. This manuscript is the first to provide the complete sequence and structure of PKR locus. We further identify a third previously unknown haplotype with an additional duplication and show that PKR1 is a tandem duplication of PKR2 which is the ancestral copy. Finally we use evolutionary analyses to show that that this duplication likely occurred just once, however both duplicated and non-duplicated haplotypes are segregating in highly diverged species, ie is a trans-species polymorphism. Together, these results provide substantial insights into the structural evolution of a key viral restriction factor.

Second, the experiments were performed in (human) HeLa cells. Do HeLa cells accurately reflect the interactions with the virus in a bat system? Are these the cells that come into contact with the viral proteins upon entry into the body? Are these the most relevant cells to carry out these experiments in? It seems that these cells were chosen simply as a 'blank slate' to test the effects of PKR duplicates. This is ok, and many studies do this, but then this has to be explicitly stated and the narrative adjusted because I don't consider this to be an experiment reflecting the fact that bats are good models for functional studies.

As suggested by the reviewer and noted above we have modified the structure and narrative of the paper to better reflect the experiments and analyses we performed. The experiments on *Myotis* PKRs were designed to dissect the functional consequences and possible tradeoffs of having the two PKR copies in *Myotis*; the Hela PKR-KO system provides a clean, controlled model for this. As previously mentioned, using model cell lines such as HeLa and HEK293 with KOs of genes of interest is a standard way to model the functions of specific genes without the interference of other adaptations (see e.g. Morales *et al*. *Nature* 2025 with ISG15).

In summary, while the study provides valuable resources and can be used as a starting point to formulate/test hypotheses, it lacks the mechanistic depth and novelty. In my opinion, it would be better suited to a specialized evolutionary genomics journal.

> We believe that this manuscript presents several novelties that are interesting to a broader audience than evolutionary genomics. We agree that we have presented here a mechanistic breadth, rather than depth, of vignettes that highlight the remarkable biology of *Myotis* and its value to multiple disciplines. The significant number of future directions opened by this manuscript are of interest to various fields, which is highlighted in the diversity of discussion points among the reviewers for the manuscript. As such, we do believe that a general journal such as *Nature* is a better fit for this manuscript than a journal with a more narrow scope.

Referee #3 (Remarks to the Author):

The authors have characterized myotis specific immune features in bats associated with DNA damage responses and longevity and correlate what they believe are the genes linked to DNA viruses - matched to exceptional longevity. This is indeed intriguing. The addition of TE analysis etc also highlights some intriguing possiblities related to choromosmal aberrations/insertions/SVs etc and how the bats may respond to them with DNA damage machinery (I feel this is not highlighted enough). They then consequently show additional about the myotis PKR's they have already characterized but I find this in contrast to the claims of DNA-only VIPs being incredibly important in myotis (as PKR is not a DNA-only VIP, but rather an RNA-only VIP - in terms of direct interaction).

The authors should be careful with references, making sure the most appropriate reference is used - and correct. There's a reference to the author correction rather than the original article.

> We appreciate the reviewer for catching this - we have gone through the citations and ensured that we have appropriately cited the correct references. We have also reduced the number of references to simplify our text and arguments, and moved several references to the Extended portion of our manuscript where necessary.

The comment about comparison of tissue vs cell genomes is interesting but has no associated data.

> While we acknowledge that the data is not presented as a stand-alone graph, the data is presented in the statistics in the summary table and cited as such. For clarity, we have moved this observation to the Extended Data section.

Figure 1 is a little crowded and the Figure legends do not have enough detail carefully explaining each graph and what it represents.

> We have revised our text to better explain what the different elements of Figure 1 represent.

The Genome annotations appear to be using old versions of the pipelines (e.g. TOGA) so its important to mention version number.

We have added the version information for TOGA in the Methods section of the text.

Was it truly 27000 coding genes identified or 27000, isoforms?

We annotated an average of 27.5K coding genes per genome, with 21,756 genes having an annotated ortholog in at least one other *Myotis* genome (see section “Orthologous Gene Alignments” in Methods).

The eutheria max likelihood tree should be supplied together in the supp and is there previous validation/other literature confirming time calibration methodology (if so, reference it).

Our pan-mammalian phylogeny is in strong agreement with other previously-published *Eutherian* phylogenies, including phylogenies of 39 mammals(Romiguier et al. 2013), of 241 placental mammals(Foley et al. 2023), and of 11 nearctic *Myotis* species based on SINEs(Korstian et al. 2022). This note has now been added to the text in the Methods.

The exceptional examples should be highlighted visually on the lifespan tree in Figure 2 somehow.

Due to space constraints, we were unable to clearly highlight these examples; however, we do list the exceptional examples and the relevant statistics in the text, and include our full results in Table S6.

I am concerned about the conclusions drawn for figures 2 E/F - the RICR was modelled by the assumption that lifespan or bodysize directly correlate to a constant for cancer risk. The authors are now claiming that lifespan separates out disproportionately for cancer risk in bats (therefore breaking the assumption fo how the RICR was originally modelled)?

We would like to clarify how RICR is calculated. RICR has always been modeled as the ratio between a node and its ancestor of the product of their lifespan *and* body size. In previous studies (Vazquez & Lynch 2021, Vazquez *et al* 2022) RICR was only considered in the context of *Afrotheria* and *Atlantogenata*, two clades which show significant changes in body size, but not lifespan. This study improves on the prior work by discretely modeling the evolution of lifespan (as opposed to applying a phylogenetic linear regression model to estimate ancestral lifespans given body size), and by using a larger phylogeny of over 1000 mammals with body size and lifespan estimated; importantly, this phylogeny (Upham *et al.* 2019) resolves polytomies in the *Myotis* clade that were present in the phylogenies used in prior work. This work is also the first to specifically rank RICR across mammals, leading to our finding that lifespan contributes to a disproportionate change in cancer risk *in bats*; this does not contradict prior findings that body size plays a major role in the cancer risk of clades such as *Afrotheria*. More recent studies on Peto’s Paradox (e.g.(Compton et al. 2024)) have shown that while

body size appears to be potentially weakly correlated with cancer risk - although not to the extent that would be predicted by statistics - no such relationship can be found for lifespan. This further underscores the importance of studying the influence of lifespan independent of body size on cancer risk.

It would be important to ensure there is no bias introduced by the length of the phylogenetic tree (and ccumulation of mutations etc) and it is directly a function of the difference between e.g. lifespan/bodysize of species X and its common ancestor.

We agree with the reviewer that phylogenetic sampling is a concern in these types of analyses. All of our statistics incorporate phylogenetic correction where relevant, and this is stated in the manuscript (e.g. use of phylogenetic ANCOVA for analyzing body size and lifespan, Extended Data Fig 6). Because RICR is calculated based on the direct ancestor of each node, missing data can result in a mis-estimation of the rate of change in body size and lifespan in the tree - but equally enlarges the confidence interval of our estimates. Our evolutionary analysis of body size, lifespan, and RICR encompasses 1157 species, with robust sampling within each major mammalian clade including bats and *Myotis*. Furthermore, we ran *StableTraits* twice with eight distinct MCMC chains and 100,000 iterations of sampling to ensure both robust median body size and lifespan estimates, and robust confidence intervals for these estimates. As such, we are confident that our results are not significantly affected by the length of the phylogenetic tree.

Furthermore, we now include in Extended Data Fig 6 a plot showing the positive correlation between RICR and the percentage of overrepresented Reactome pathways that are involved in cancer; due to the small number of species and nodes tested in this analysis, however, these results are underpowered for statistical testing.

22.7% of all genes (single copy orthologs) being under selections seem quite high - what would be the expected number of genes be (relative to time/distance of the phylogenetic branch)? This could completely change the results simply based on evolutionary time between species.

We have edited our text to clarify that an average of 5.46% of protein-coding genes had at least one region under positive selection per node at FDR <=0.05; in contrast, an average of 20.8% of protein-coding genes per node at FDR <=0.05 had all regions with an omega less than 1, suggesting negative selection. Our selected method, aBSREL, incorporates controls for expected dN/dS for each sequence using the species phylogeny.

There are very methods in the details for the absrel analysis, including which settings/model were chosen and the justification for why.

We have added additional details on how we ran aBSREL in the respective subsection of the methods.

- immunity, cancer and aging are three processes yet you talk about the intersection of two processes ??

We thank the reviewer for catching this error - we have changed the text to state: “Many of these genes lie at the intersection of multiple processes.”

Relax and RERconverge were very poorly described in the main text with no real justification for their usage etc (you cannot just explain in the methods but the main text requires some commentary).

We agree with the reviewer that our treatment of the RELAX and RERConverge results was insufficient. For the sake of clarity in the main text, we have moved our RELAX and RERConverge analyses to our Dryad archive, as these results - while important and interesting - are less relevant to our main points.

Many of these genes have already been published as being under selection in bats - there needs to be a more consistent matching of the findings with existing literature.

We apologize if we mistakenly implied that many of these genes were previously unknown or unstated as under selection in *Myotis*. We discuss in the results and discussion how our results compare to other publications, such as Morales *et al.* 2025.

The novelty of this study is our ability to resolve *when* in the phylogeny each gene has undergone selection, as prior studies have been limited to the European-Nearctic *Myotis* split due to available genomes. Our ultimate goal in this study is to highlight the pleiotropy between immunity and longevity that we observed among both the pathways overrepresented among genes under selection and the pathways overrepresented among genes that are differentially expressed after DNA damage.

The differences in apoptosis and viability between the cell lines is very minimal and as there is only one representative cell line for each species it is essential to show there is a true species-level effect and not a cell-line-specific defect.

We apologize for any confusion - in the Methods section, we described how we used cell lines from 8 individuals of M. lucifugus, 8 individuals of M. evotis, and 2 individuals each for the three megabat species (provided by the SDFZ). Each cell line was tested in quadruplicate per experiment, and the experiments were run twice, for a total of 8 replicates per treatment and cell line. Thus, this is a true species-level effect and not a cell-line-specific defect.

It has already bee shown that bats express varying levels of ABC transporter proteins affecting uptake of DNA damage reagents (e.g. ABCB1), it would therefore be essential to show levels of these proteins between cells and prove with multiple different DNA damaging reagents.

While ABCB1 was shown to pump out two DNA-damaging drugs, etoposide and doxorubicin in Koh *et al.* (2019) , it is worth noting that this same paper demonstrated that doxorubicin accumulated in bat cells until the media was washed out - ABCB1 does not prevent the initial entry of these drugs, and cells reach a stable equilibrium of drug concentration inside of cells. In our manuscript, we left the cells in treatment media continuously for 24 hours - not for a limit of 3 hours followed by recovery as in Koh *et al*. Additionally, because of the findings of Koh *et al*, we chose from the onset to avoid the

issue entirely by using neocarzinostatin: as a holoprotein, it enters the cell via endocyotsis (see https://pubmed.ncbi.nlm.nih.gov/2528008/) and is not removed by by ABCB1, which targets small molecules and not large proteins (see https://pmc.ncbi.nlm.nih.gov/articles/PMC6800160/); and its mechanism of action is direct, and not dependent on TOP2 activity (unlike etoposide and doxorubicin).

For Figure 4 - the inserts of non-myotis bats needs to have the same scale as the myotis bats (with numbers included, just like myotis bats) and is there a reason for including all the non-significant data? A standard P-value would be 0.05 and so this number is the most important. At this P-value there is 1 more VIP for RNA compared to DNA (51 vs 50), though yes the ratio is different. To me, the P-value of 0.05 is the only thing worth considering - if not justify how non-significant data is worth including/discussing ??

We agree with the reviewer that the inserts should have the same scale as the main figures, with numbers of VIPs included. We have made these changes and moved the inserts to separate panels for readability. Our analysis of VIPs compares the enrichment ratio of VIPs compared the background at different p-value cutoffs. Comparing the distribution of this enrichment across the full range of p-values is a more statistically rigorous approach than selecting a single p-value. The reviewer is correct that the key statistic is not the number of VIPs, but the enrichment ratio, which is well beyond expectation for the DNA-only VIPs, but at ~1 for the RNA-only VIPs. This highly significant enrichment across the full P-value distribution is indicated by the shaded confidence intervals. We have clarified this in the text.

There is nothing in the methods about defining what is a DNA-only VIP vs a RNA-only VIP. how was this decided from the original VIP dataset (which is ~25% of all genes anyway at >5000).

We thank the reviewer for pointing this out. We have classified VIPs by what types of viruses they interact with: DNA viruses, RNA viruses, or both. These classifications are based on the experimental literature from which the VIPs were defined, as described in Enard et al. 2016 and Souilmi et al. 2021. Furthermore, DNA-only VIPs are VIPs that only interact with viruses whose genomes are encoded by DNA, and RNA-only VIPs are VIPs that only interact with viruses encoded by RNA. We divide them this way because the fundamental genetic material making up a viral genome is an important characteristic of viral biology, and it has been previously found that the DNA/RNA virus distinction in humans is evolutionary relevant because it is RNA viruses that have driven most adaptation in humans. Furthermore, most zoonoses are caused by RNA viruses. We have clarified this in the text, as well as included an additional supplement of the list of viruses each VIP is known to interact with.

What about RNA/DNA binding VIPs since many viral pathways/sensor overlap depending on the virus? Ref. 109 has 1300 proteins - you should maybe mention the total of genes and the total number of orthologs (matched to the ref paper) as this is misleading. That paper is also quite old now and many VIPs have been further

characterized since then - particularly with regards to binding both DNA/RNA instead of just RNA-only etc. It's not possible to really evaluate this figure without that information.

The reviewer's point that many VIPs have overlap in interacting with both RNA and DNA viruses is well-taken, and reflected in the "All VIPs" panel of the figure. This emphasizes the need to separate out interactions with DNA viruses vs interactions with RNA viruses (as represented by the "DNA-only VIPs" and "RNA-only VIPs," which we have now better defined in the text), since looking at VIPs regardless of the types of viruses they interact with obscures the finding that virus adaptation in bats is driven by interactions with DNA viruses. As we state in a separate response and clarified in the Extended Methods, our new set of VIPs is an update to the original Enard et al. 2016 and Souilmi et al. 2021 datasets based on the most recent literature search since 2021.

How many of the VIPs are viral-specific may indeed be more interesting - once subtracting the DNA damage genes etc from the VIP list (as many moonlight and would be present in the VIP list).

We interpret the reviewer's statement about "viral-specific" VIPs to be referring to proteins that only interact with viruses and have no other role in host biology. We respectfully disagree that this would be more interesting, as this would limit the analyses only to proteins specialized in anti-viral immune defense. Viruses have been shown to drive adaptation across a broad array of mammalian proteins, including those with key roles in host biology that might otherwise be expected to be under strong constraint. The roles of VIPs in host biology are part of why viruses interact with these specific proteins - many VIPs have cellular functions in the host that the virus hijacks, for example replication machinery. It is the adaptation in these genes that reveals the genome-wide importance of viruses as drivers of evolutionary adaptation in mammals. Restricting the analysis to only immune genes also wouldn't be informative about the kinds of viruses (DNA viruses or RNA viruses) that are more important influences on the host. Furthermore, the overall conservation of these genes (related to their roles in host biology) is something we account for in the confounding factors by matching dN/dS ratio between VIPs and controls, with the expectation that the dN/dS ratio in the VIPs is elevated due to their additional interactions with viruses that the control genes don't share.

Fig 5A/B/C is interesting but how it correlates to the whole story is not clear. it could easily be placed in the supplemental. There's no link.

We greatly appreciate the reviewer's perspective here. In order to clarify the central narrative of the manuscript, we have rearranged the sections to more naturally link our observations from the structural variation observed in near-complete assemblies, to the examination of the relationship between genetic variation, pathway-level enrichments, and investigations on specific biological processes.

Fig S5I-J is interesting and fits - though again there's no control for bias based on evolutionary branch length / representation over and above expected evolution etc. The gene duplications etc do not match any virus pathway - but do match this VIP database -

explain the inconsistency. The gene duplications etc do not match any virus pathway - but do match this VIP database - explain the inconsistency.

We have noted the lack of clarity in our analyses in Fig. S5I-J. The results from CAFE (Mendes et al. 2020) incorporate evolutionary branch length and models of gene family birth-death processes, providing a control for bias in these factors. We have added additional text to clarify how these distributions are generated from the results of repeated bootstraps of CAFE analyses. As previously mentioned, VIP classification is based on the experimental literature, as described in Enard et al. 2016 and Souilmi et al. 2021. We note that many VIPs shown to be experimentally involved in the viral life cycle may not be characterized explicitly within viral pathways available on databases such as Reactome, as viral interactions are not their primary function.

What proportion of VIPs sit outside the single-copy ortholog collection (are they normally duplicated anyway - even if duplication etc is an antiviral strategy). evolutionary time-bias is therefore an important consideration also. longer time = more duplications, regardless of species. Please explain how clearly how this is controlled for.

88% of the VIPs in our dataset are 1:1 across Nearctic *Myotis*; the remaining 12% have 2+ copies in at least one (in some cases only one) species.We would like to clarify that CAFE uses a time-calibrated phylogeny, and takes both evolutionary time and tree topology into account in its birth/death models.

There is very little linking immunity or viral infection with Figure 5 at all.

We apologize for the confusion that may have resulted from the ordering of the text. The original goal of Figure 5 was to showcase the varied structural variation landscape across 9 nearctic *Myotis* species, which was previously nested between two narratives of viral innate immunity. This has been corrected in the new ordering of the text.

Figure 6D must be quantified to show the amount of pull-down (though yes, it seems clear from the image). Figure 6E is interesting but the conclusion it is additive cannot be drawn from the experiment.

We appreciate the reviewer's suggestion, and have incorporated this feedback into the new Figure 2.

While it does not appear synergistic or dominant negative the authors would need to perform experiments with 50% PKR1 + 50% EV and see the functional capacity of 350ng PKR1 compared to 700ng of PKR1 to see if the effect in PKR1/2 mixes are due to solely PKR1 or truly PKR2 + PKR1.

Yes, this is the setup we used. We have now clarified the Method and Result sections.

The amount o DNAis ambiguous also as each experiment in E/F/G uses a different amount of DNA. The viability assay is good using small amounts (and is clearly not additive for viability at 100ng). The level of translation shutdown in E does not match viability in G (for 200ng). Its interesting to note that strong viability effect just from low-

dose transfection alone, in the absence of polyIC treatment. The subtleties of this should be more carefully explained.

We thank the reviewer for highlighting this observation. As previously shown by other labs (Elde, Rothunberg Labs…) and in the transfection setup we use, PKR is already activated, even without polyIC induction, by sensing of cytosolic nucleic acids resulting from the transfection.

For Figure F, the addition of PKR1/2 (at high doses) in the presence of an activating stimulus (dsRNA from VSV) - may be suggestive of an interaction not seen at the basal state. The results from S6C show it is not black and white 'additive' and clearly not as simple as the authors make it sound.

We thank the reviewer for their insightful comment. We agree that there is a lot of nuance in the innate immune response, which is why we chose to use a non-*Myotis* cell line to characterize PKR: this allows us to examine the functions of PKR1/2 in a controlled context. However, we do agree that it is interesting to test PKR interactions in the context of viral infections. We therefore performed the same coIP experiments in the presence of SINV infection and have now added these data in **Extended Data Fig. 4**. It showed that, even in the context of viral infections, PKR1 and PKR2 from *Myotis* did not dimerize.

For example the protein shutdown effect is known to block VSV but nfkb is also pro-viral for VSV (and IFN strongly antiviral) - so the 'tradeoff' has to be carefully explained. It would be easy to test in the presence or absence of polyIC dsRNA to induce dimerization/activation or in the presence of a K271/K296R point mutation that would disable eIF2a phosphorylation but still maintain PKR's scaffold-pro NFkB role.

While other studies in bats have shown selection in NFKB in *Myotis*, how this influences PKR is outside of the scope of this study. The use of a HeLa PKR-/- line for our testing ensures that we are controlling for the influence of NFKB in our experiments. Most of the antiviral phenotypes we observe are consistent with translation shutdown. We cannot exclude that other functions of PKR such as NFKb play a minor role but that would be the scope of another study.

How RNA virus induction correlates to the title of the manuscript is unclear. (except the already published VACV data).

We apologize for the lack of clarity in our text. To fix this common comment, we have edited the manuscript to clarify that PKR is also a DNA VIP; and we have rearranged the text to more clearly connect the focus of PKR as an exemplar trans-species polymorphism in our system. For more discussion on RNA VIPs in the context of our DNA-only VIP findings, please see the last comment for this reviewer and our response.

Consistently throughout each section there are differences in the source data used including different number of genomes, different number of non-myotis bats, different number of other animal hosts. Its quite confusing to follow carefully and the greater

impact of how the missing data may impact one single analysis is uncertain. As there is no subsampling, downmixing etc it is very hard to say how the numbers of each bracnh may bias the results.

We have simplified the text to clarify and better explain the differences in sample size between the analyses (see responses to Reviewer #1). We also agree that having additional megabat samples would have been ideal. We included in our experiments all the individuals that were available to us via the San Diego Frozen Zoo; unfortunately, due to the difficulty in obtaining samples of CITES-protected species, we are unable to increase our sample size for these species. We do however note that in these species, there is no significant difference between individuals' NCS response.

In terms of the conclusions - while indeed wing punch - cell lines, genomes etc is a powerful approach - the authors identified an RNA sensor (an RNA-only VIP) that has a strong effect in myotis. This is in contrast to the claims throughout the paper that DNA-only VIPs are more important in myotis. While PKR is important during DNA virus infections it is also important in RNA virus infections and not DNA-only. Other examples may have been much better for functional data, particularly considering the importance of myotis PKR in DNA virus infection was already published by the team. The use of PKR with RNA virus infections, while interesting to me, does not particularly add anything to justify the claims from the bioinformatics.

We appreciate the reviewer's summary of the conclusion in this way, as it has helped us to realize that the way we presented the dichotomy between genome-wide adaptation in response to DNA viruses and specific cases of adaptation in response to RNA viruses was not sufficiently nuanced. We have changed the way we present our results to emphasize that, while bats have adapted to both DNA viruses and RNA viruses, adaptation to DNA viruses is required beyond the first line of defense of antiviral proteins. In contrast, our results show that bats' specialized immune adaptations provide an adequate first line of immune defense to protect against fitness impacts of some (but not all) RNA viruses. This results in broad-scale adaptation across DNA virus VIPs. This is in contrast to other mammals, in which the first-line immune defence is either not sufficient to protect against the fitness impacts of DNA or RNA viruses on its own, or is sufficient to protect against the fitness impacts of DNA viruses but not RNA viruses (primates). In addition, we have added additional clarification about the difference in relative literature focus on RNA viruses (because of their zoonotic potential) vs DNA viruses. More is known about bat immune adaptations to RNA viruses because these are the viruses that have historically had the most impact on humans. Finally, we have reworked the manuscript to focus on several broad scale genomics conclusions we have been able to make from our newly assembled genomes, genomic analyses and experiments.

Referee #4 (Remarks to the Author):

In their manuscript entitled: "Extensive longevity and DNA virus-driven adaptation in nearctic Myotis bats" Vazquez and colleagues investigate the evolution of extended longevity and infectious disease resistance. The study includes several major findings including the demonstration that virus adaptation in bats is mostly driven by DNA viruses. In addition, it provides chromosome-scale genome assemblies for eight new species of bats, an important resource for future projects on these potential reservoirs of zoonoses.
This paper addresses several fundamental questions regarding the evolution of extended longevity in bats with a powerful combination of new data sets. I enjoyed reading it but I think that the paper often tries to bring together some analyses, approaches or questions that are difficult to combine at the current level of understanding of this system.
For example, I was much less convinced by the section of the manuscript investigating the evolution of cancer resistance and I feel that this aspect of the study weakens this paper, in comparison to the findings and section on virus adaptation. First, I find the section using cell cultures superficial compared to recent papers using this approach in several vertebrate species (Firsanov, D., Zacher, M., Tian, X., Zhao, Y., George, J. C., Sformo, T. L., ... & Gorbunova, V. (2023). DNA repair and anti-cancer mechanisms in the longest-living mammal: the bowhead whale. BioRxiv), including a very recent paper in Myotis bats (Hua, R., Ma, Y. S., Yang, L., Hao, J. J., Hua, Q. Y., Shi, L. Y., ... & Liu, Z. (2024). Experimental evidence for cancer resistance in a bat species. Nature Communications, 15(1), 1401.). Establishing these cell cultures did not bring any clear results or identification of a mechanism of cancer resistance in myotis in the current paper. In addition, establishing cell culture from wild organisms is now quite common and should not be presented has a new tool or innovative component of this paper.

We thank the reviewer for their constructive feedback. Our manuscript indeed presents more mechanistic breadth than depth with vignettes that highlight the remarkable biology of *Myotis* and its value to multiple disciplines. We appreciate that the manuscripts cited by the reviewer represent significant efforts in addressing more targeted questions, however are also among a select few publications that leverage functional genomics in primary cell lines for comparative biology. We have reorganized the manuscript in response to the reviewers insights to 1) provide more emphasis on the areas of the manuscript which the reviewer suggests are strong, 2) strengthen the experimental evidence of individual vignettes. We believe that these strengthen the manuscript overall and highlight the future directions to be explored by researchers across various disciplines.

Secondly, while it's often assumed that bats are cancer resistant (see, in the current manuscript Line 479: Intriguingly, these species demonstrate some of the lowest known rates of cancer among mammals or Line 427: Bats are widely known for their long lifespan, cancer resistance, and viral tolerance), I think it's simply not true and not based on actual epidemiological data. Recent large scale comparative analyses using necropy data show that cancer mortality rate or cancer prevalence vary a lot across bat species (Vincze, O., Colchero, F., Lemaître, J. F., Conde, D. A., Pavard, S., Bieuville, M., ... &

Giraudeau, M. (2022). Cancer risk across mammals. Nature, 601(7892), 263-267. and Compton et al. (2024). Cancer across vertebrates. Cancer Discovery), with some bat species showing quite high cancer rates compared to other vertebrates.

We have revised our manuscript to tone down the language of cancer resistance in bats. The reviewer is correct that there is significant variability of cancer *rates* in bats which highlights the strength of using chiroptera as a comparative system to study the evolution of cancer risk. Our own RICR estimates firmly support this, with bats present across the spectrum of RICR changes in mammals. We note that Hua *et al*. 2024 (cited by the Reviewer in the previous remark) showed empirically that several bats have increased resistance to tumorigenesis compared to mice and humans. Additionally, in the "Cancer Leaderboard" published in Compton *et al.* 2024, there are no bats in the top 20 species by cancer incidence, whereas bats are 5 out of the 20 species with the lowest incidence of cancer.

Finally, while I know that the Relative Intrinsic Cancer risk is a metric that has been used in other studies (mostly, but not only, in papers with some co-authors of this paper), I am still not completely convinced that this variable truly reflects cancer susceptibility across species (but I see that it can be used to just identify species under strong selection for the evolution of cancer resistance mechanism(s)). First, it assumes that a certain number of consecutive changes in the genetic material are needed to transform a normal cell into a cancer cell and that "Within species, lifetime cancer risk scales linearly with body size, and with lifespan by a power-law of exponent 6" (Line 176-177). I feel that we do not have evidence that this is true across species. On the contrary, we already know that naked mole rat or bowhead whale cell lines for example need different number of mutations for malignant transformation. So, can the authors explain the rationale behind the use of this variable across species and how it fits with the recent work of Cagan et al. (Cagan, A., Baez-Ortega, A., Brzozowska, N., Abascal, F., Coorens, T. H., Sanders, M. A., ... & Martincorena, I. (2022). Somatic mutation rates scale with lifespan across mammals. Nature, 604(7906), 517-524.)?

RICR does not assume that there is a universal set of mutations, or mutational load, in multicellular organisms that is required for malignant transformation - quite the opposite. The only two assumptions are that individuals of all species acquire mutations over their lifespan; and that body size differences between individuals and species are mostly due to differences in cell count.

The first assumption is the central conclusion of Cagan *et al.* (2022): all mammals studied acquire somatic mutations over their lifespans, and the rate of somatic mutations is inversely related to species' lifespans. Lowering the rate of somatic mutations is one mechanism (but not the only mechanism) to reduce cancer risk, and it would be a consequence predicted by RICR. This is the hypothesis that underlies our decision to look at DNA damage sensitivity in this manuscript, as increased sensitivity to DNA damage (defined as increased cell death) is one way to reduce the accumulation of somatic mutations within an individual.

The second assumption has also been shown to hold true for most cell types with a handful of cell-type specific exceptions (Savage, Allen, and Brown, 2007). The effect of

cell size scaling to body mass, however, is still a rounding error compared to the differences in cell count.

The different number of mutations needed for malignant transformation in mice, humans, naked mole rats, and bowhead whales is, in fact, an excellent example of how *intrinsic cancer risk* can change to compensate for changes in body size and lifespan. Similar to our somatic mutation argument before, this is one way - but not the only way - to reduce the cancer risk.

Second, the metric does not include real cancer prevalence data that are now available for hundreds of vertebrate species. In other words, two species of similar size and lifespan will obtain the same Relative Intrinsic Cancer risk score but based on epidemiological data published, we know that these two species might show really different cancer prevalence.

The reviewer is correct that this could theoretically happen: for example, one can imagine that in the presence of a strong external influence on cancer rates, such as environment or diet, two sister species that share a most recent common ancestor, and have identical lifespans and body sizes, will have identical RICRs but different empirical cancer rates. We would argue that these species represent outliers for any genetic analysis of the evolution of cancer resistance, and as such, RICR represents a highly conservative metric for identifying points in evolutionary history for studying changes in cancer risk and resistance due to changes in intrinsic life history traits. We do agree, however, that an improved model could incorporate these external environmental and epidemiological data for a more nuanced approach that could dissect adaptations to the environment that are associated with changes in RICR and empirical cancer risk; however, as we elaborate in the next point, the use of empirical cancer data is not feasible at pan-mammal scales in the current time.

A real and very useful tool to detect species that have evolved cancer resistance mechanism would instead combine the Relative Intrinsic Cancer risk variable used in this study with real epidemiological data on cancer prevalence.

We strongly agree with the reviewer that the combination of RICR and empirical cancer rates would be a gold-standard tool for studying the evolution of cancer risk and resisitance. However, this is only feasible for a small fraction of species where we can obtain these estimates in an unbiased manner. A major limitation in the field of comparative cancer biology is that there are 6,495 species of mammals (Burgin *et al*. 2024, *Journal of Mammology*), and there are significant biases in sampling data both within and between each order. This is especially true in bats, where only a miniscule fraction of bats can be kept in captivity (Barnard 2012, “Bats in Captivity”). As most of the longest-lived bats cannot be maintained in captivity - and are difficult to necropsy due to their small sizes - there will need to be significant improvements in our ability to detect neoplasms in wild populations to compensate, such as sequencing cell-free DNA in plasma to detect tumors in life or euthanized animals.

Other comments:

Abstract: I found the abstract a bit weak, the last sentence, for example, is not useful and does not reflect the impact that this paper can have on this topic and the scientific community.

We have revised the abstract along with the rest of the text in order to better highlight the impact of the paper

L179: "The observation of similar lifetime cancer incidence rates across mammals". I disagree with this sentence, mammalian species have very different lifetime cancer incidence, it has now been shown several times.

We agree with the reviewer that this is an inaccurate statement of Peto's Paradox, and have removed this statement.

L188-196: I think that the authors should provide some real statistics to support this. i.e. Myotis show some of the most pronounced decreases in RICR among mammals.

Unfortunately, there are no neoplasia records for most species of bats, and little hope to obtain unbiased estimates of cancer rates from Vesper bats such as *Myotis*, which cannot be successfully kept in captivity. However, from Compton *et al*. 2024, we do not see bats present among the 20 species with the highest rates of cancer; conversely 5 out of the 20 species with the lowest rates of cancer are bats. Experimentally, it has been shown by Hua *et al.* (2024) that at least one *Myotis* species has increased resistance to tumorigenesis *in vitro*.

L204: three pathways are mentioned in the previous sentence (not two).

We thank the reviewer for catching this - we have changed the text to correct this mistake.

L231: Why 11 species? Which species?

At the time of this study, we had access to only 11 *Myotis* species with well-annotated genes in our dataset: the 8 *Myotis* species highlighted in Figure 1, *Myotis yumanensis*, *Myotis brandtii, Myotis myotis,* and *Myotis davidii*.

L244-260: Why would you see a decrease of viability and an increase of apoptosis in response to a radiomimetic agent in a species with an increase ability for DNA repair. I find it counterintuitive.

We agree that this result is counterintuitive. Our hypothesis is that there is a threshold effect: it may be that long-lived bats are able to repair low levels of DNA damage with higher fidelity, while at higher levels (which are more likely to cause irreparable mutations) they are more readily able to switch to a pro-apoptotic program. Our new RNA-seq results show that DNA repair pathways are enriched among *down-regulated* genes after exposure to a high dose of neocarzinostatin, which is in line with this hypothesis.

Dear Referees,

We present our newly revised manuscript titled “Extensive longevity and DNA virus-driven adaptation in nearctic Myotis bats.” As requested, we have tracked and highlighted our edits to the text; for clarity, we additionally present a clean copy of the manuscript with all changes implemented. Additionally, please note that we have restructured our supplements and extended materials to confirm with editorial guidelines.

We appreciated the positive feedback, which highlighted our rigorous analyses and the welcomed inclusion of our RNA-seq data and novel conclusions in Figure 6. While most comments were noted to be minor, on review of the manuscript after considering your comments, we have elected to remove our RICR analyses in order to streamline the narrative of this manuscript. Many of our results can be explained by the rapid increases in lifespan seen in *Myotis*, and by our findings of pleiotropy between anti-viral and pro-longevity processes. In addition to this change, we have incorporated your feedback throughout the manuscript, and have tracked changes as requested.

We believe that this work presents a number of important results and conclusions that are of interest to an interdisciplinary readership. Our study uncovers new ties between the evolution of lifespan, cancer resistance, and virus response in bats thus contributing both to our understanding of the etiology of the many extraordinary phenotypes found in bats. We believe this study provides a new perspective on the evolution of aging and longevity across the tree of life. We hope that the additional improvements made in response to your thoughtful comments further highlight the broad interest and exciting future work enabled by our study.

For clarity, we have answered each reviewer in-line with their comments, and indented and highlighted our responses for clarity.

Referees' comments:

Referee #1 (Remarks to the Author):

The authors have done an excellent job in revising the manuscript, which has been substantially improved; many of the earlier issues have been corrected or further clarified. The inclusion of the new RNA-seq data provides valuable additional insight into the DNA damage response in Myotis cells. The section on intrinsic cancer risk is now clearly articulated, and the experimental results are generally more interpretable. The observation of the trans-species heterozygous PKR duplication is an impressive demonstration of the potential of haplotypic resolved genomes.

I am fully supportive of the publication of this paper, provided that the authors address the following comments, some of which pertain to earlier remarks.

- LPP scores from ASTRAL-IV are not displayed in Figure 1. At a minimum, it would be helpful to indicate nodes with low support, e.g. the root of Nearctic Myotis. The statement in line 103, "Using these annotations, we resolved the conflicting phylogenies of Nearctic Myotis," should perhaps be toned down, as the low root support suggests the phylogeny remains only partially resolved.

The LPP scores from ASTRAL-IV are shown in Extended Figure 2B (for all mammals) and 2C (bat subtree). We now highlight in Fig. 1B and its legend that the root of Nearctic Myotis has low support in the text and figure. All other nodes in the bat phylogeny have 100% support. We have now reworded the sentence to highlight the uncertainty.

- The reported average of 27,536 protein-coding genes appears to be substantially inflated. Mammals, including bats, typically have around 20,000 genes. This suggests that the annotation likely contains fragmented or erroneous genes. The high figure is particularly striking given that the BLAT approach described in the methods added approximately 1,800 further genes.

We agree that the number of predicted genes appears high. When excluding predicted protein-coding genes that do not have a named mammalian homolog (e.g: TP53 or a Zinc-finger family gene), there are only an average of 20,869 genes annotated across all 8 genomes. We now report this number for clarity in the main text.

- In the previous version, line 138 read:
"To resolve the phylogeny of Nearctic Myotis, we identified single-copy orthologs of 17,509 protein genes present in 536 mammalian genomes,"
whereas the revised version states:
"A phylogeny of all 534 mammals in our alignments,"
and the number of genes changed to "15,734 gene alignments spanning 534 mammals."
Since the methods description appears unchanged, the authors should clarify which two species were removed and the rationale for the reduction from 17,509 to 15,734 genes.

We apologize for the confusion. The original text had typos which were corrected. There are 536 total mammals that are included in our alignments and trees, and 15,734 genes in the alignments. Related to this, there were two missing assemblies in Table S2 (*Canis lupus familiaris* and *Canis lupus dingo*) which lead to the erroneous "534" number. These issues have been corrected.

- The authors state that "The table including the list of species and orthologous gene alignments are now included in Table S2."
Table S2 does indeed contain the list of species, but not the gene alignments, which would be a useful resource for future studies.

All orthologous gene alignments were included as multiple sequence alignments uploaded to Dryad, visible at the following link for reviewers: https://datadryad.org/share/LINK_NOT_FOR_PUBLICATION/VLwqtJ4gv1joM8_0JaZen4y7Un2TjGcudZXluzjbpLQ. As the titles were unclear, we have now renamed them for clarification.

- Adaptation to RNA viruses appears to be primarily driven by changes in gene copy number, as shown in this study. I therefore find the statement in the abstract—"but not RNA viruses"—somewhat misleading, and would suggest instead highlighting the distinct modes by which DNA and RNA virus-interacting proteins evolve.

We have rewritten the abstract as a Summary Paragraph reflecting these suggestions.

- Figure 4 contains minor visualization issues: some panels appear shifted upwards, likely due to the omission of panels J–L.

We have now corrected this to repair all visual artifacts.

- Line 116: the 20-million-year estimate conflicts with the 33-million-year figure reported in line 63.

We thank the reviewer for pointing this out. The estimated divergence according to Morales *et al*. (2019) is 26-47 million years, and is no less than 33 MY according to fossil records from Gunnell *et al*. (2017). We have corrected this estimate in the text.

- Line 172: it should be clarified that PKR1 + PKR2 co-expression refers to a half-dose of each gene. It would also be useful to note that, despite PKR1 being expressed at lower levels, it consistently proves the more potent of the two duplicates in all experiments.

We have noted the half dose of *PKR1* + *PKR2* and added text commenting on the increased potency of PKR1.

- Line 293: consider rephrasing as "Genes under positive selection were enriched," given that the preceding sentence refers to negative selection.

The analysis in this particular sentence refers to genes under both positive and negative selection (Extended Figure 6). However, subsequent analyses refer to genes under positive selection, and thus in the next paragraph the text has been modified to read "positive selection" throughout for clarity.

- The abbreviation "NCS" should be defined at line 314.

We have added this additional clarification to line 314: "neocarzinostatin (NCS)."

- Line 362: the authors likely refer to Yangochiroptera.

> We greatly appreciate this catch - we have fixed this typo, replacing “Yinpterochiroptera” with “Yangochiroptera”.

- There is also a minor typographical error in line 178: “e recently duplicated PKR1s homodimerized.”

> We have fixed this error by adding the missing text (bolded): “**While th**e…”

Referee #2 (Remarks to the Author):

The authors have improved the manuscript by restructuring the text and refining/toning down the language to more accurately reflect the scope and nature of their findings. The presentation is now clearer and more cohesive. The addition of the RNA-seq analysis provides further support for their conclusions, particularly by linking DNA damage responses to immune pathways, and represents a valuable enhancement to the study. The PKR analysis is interesting in the sense that the availability of complete genome sequences allow the authors to conduct evolutionary analyses that clarify the history and structure of this known duplication. However, I do not believe the functional analyses presented in this section offer substantial new mechanistic insights beyond what was previously reported.

I commend the authors for the thoughtful and constructive revisions they have made in response to the reviews. The science is rigorous, and the authors leverage high-quality, haplotype-resolved genomes to generate well-supported insights within a strong statistical framework. Nonetheless, I maintain my original assessment that the main findings largely reinforce existing knowledge about the evolution of key traits in this system. In my view, the degree of conceptual novelty and the extent to which the work advances the field are more modest. It may therefore be appropriate for the Editors to consider whether the manuscript is best suited for a broad, general-interest journal or a more specialized venue.

> We appreciate that the reviewer considers this manuscript to be interesting and rigorous. We believe that this work presents a number of important results and conclusions that are of interest to an interdisciplinary readership. Our study uncovers new ties between the evolution of lifespan, cancer resistance, and virus response in bats thus contributing both to our understanding of the etiology of the many extraordinary phenotypes found in bats. We believe this study provides a new perspective on the evolution of aging and longevity across the tree of life. We hope that the additional improvements made in response to the reviewers’ thoughtful comments further highlight the broad interest and exciting future work enabled by our study.

Referee #3 (Remarks to the Author):

The authors have modified the flow and arrangement of the manuscript considerably since the first submission and here they present data on longevity and virus-driven evolution in myotis bats. The abstract and introduction read much better and have a lot of clarification. The data on the additional myotis genomes are presented clearly with statistics etc across figures 1 and 2. There is little effort to correlate their findings to the literature in terms of all the other myotis genome publications including the one last year (https://academic.oup.com/mbe/article/41/9/msae165/7730189?login=false#).

We thank the reviewer for bringing this up - we made an effort to compare our findings to the broader literature throughout our manuscript, rather than solely in the discussion, including Morales *et al.* (2025) and others. We have now added additional text throughout the manuscript highlighting comparisons of our selection analyses and other results.

Figure 4A is nice however its really odd that some of these bats (like auriculus) are not presented later on in Figure 5 D. Figure 5D also adds griescens/brandtii/myotis etc and so it would be better if these were also included in Figure 1A with their longevity quotient.

Figure 1A-B are derived from our 536 mammals phylogeny, which is generated only from species with whole genome sequences such as *Myotis brandtii* and *Myotis myotis*, but not *Myotis grisescens*. Both *Myotis brandtii* and *Myotis myotis* are featured in Figure 1A with their branches colored based on their longevity quotients as well as in Figure 5D. Only two species of *Myotis* were excluded from our RICR analysis, which is based on a larger mammalian phylogeny from Upham *et al.* (2019): *M. auriculus*, due to poor support of its maximum lifespan; and *M. occultus*, due to its absence from the Upham *et al*. (2019) phylogeny.

The PKR section kind of comes out of the blue now, without any kind of link or lead in (1 sentence in the introduction).

We have reworded the introductory sentence to highlight its link to the previous section.

The data itself is clear and with a more logical interpretation etc. There are some slight differences, pkr2 maybe less functional to both virus and cell translation etc. The wording for Figure 4 and description of each VIP set is much clearer and easier to follow however the separation highlights what I find are some large inconsistencies in the presented data and the conclusions drawn.
In other mammals there are clearly more RNA-only VIPs under selection then DNA-only VIPs (and not the case in myotis or barely in non-myotis bats). However DNA-only VIPs are still very strongly under selection in other mammals.When looking at just truly "significant, P=0.05" data the ratio of VIP:control is 50 vs 51 - showing no difference in # of DNA-only vs RNA-only in myotis bats. The ratio is important and at 3 vs 1 there is more DNA then RNA yes.

As shown in Table S4, there are 1998 DNA VIPs (1046 DNA-only), and 4318 RNA VIPs (2654 RNA-only) - in other words, there are more than twice as many RNA and RNA-only VIPs as DNA/DNA-only VIPs. This explains why you can have the same absolute number of ~50 while having very different relative selection enrichments. We would like to stress that what matters when estimating if a specific class of virus imposed a greater pressure is the *relative* enrichment above the genomic baseline provided by the controls. In this case, while 51 RNA-only VIPs were found to be under positive selection at $p \le 0.05$, this value is not significantly different from the genomic baseline expected based on controls matched for genomic features as described in the Extended Methods. However, while 50 DNA-only VIPs were found to be under positive selection at $p \le 0.05$, this value is significantly higher than the genomic baseline based on the bootstrapped matched controls. We report the number of VIP and control genes involved in each ratio calculation not as an indication of significance of the raw number, but as a reference for the number of genes involved in calculating the VIP:control ratio and thus the selection enrichment (or lack thereof). We have added text to the figure 4 legend to clarify this nuance.

These figures look really poor and maybs its the PDF generation problem but it seems to be badly photoshopped. Ensure the scale is on each figure (d, e, f etc not just on the left side of D).

We apologize for the graphical artifacts in Figure 4; these have now been corrected. Additionally, we have added the scale to the side of each figure.

In non-myotis bats the ratio is ~4 to ~ 1.5 - so about the same. Meaning there is no difference between myotis or non-myotis bats in this regards. The ratio of RNA-only VIPs is very similar to all other mammals (between 1-2) and so the only thing you can claim is that - unlike other mammals, it is much more likely that DNA-only VIPs are under positive selection in (all) bats, regardless of their longevity quotient/status etc.

We agree with the reviewer that DNA-only VIPs are under positive selection in all bats, and did not intend to claim that there is a significant difference between *Myotis* and non-*Myotis* bats in this respect. In the text we note: *"we found that VIP adaptation in Myotis **and other bats** is driven by selection in DNA VIPs."*

It's also strange that the number of genes is so different yet the ratio (VP:control) is not, when you are dealing with only commonly identified orthologs across the board (in the VIP sets). Overall, the conclusion that's bats have a stronger adaptation to DNA-only VIPs is valid - but it seems to be unrelated to longevity, myotis or geographic region.

Please see comment above highlighting the different total number of RNA vs DNA VIPs. In Figure 6, we demonstrate that DNA VIPs are enriched in genes expressed after DNA damage, especially when only considering genes under positive selection in *Myotis lucifugus*, suggesting a link between DNA virus response and DNA damage response (and in turn, cancer and longevity).

The Fig 4 figure legend is inaccurate.

We have carefully checked the legends throughout the manuscript for errors.

DNA-only VIPs are strongly associated with cell cycle, DNA repair etc etc (fig. 6 C-F) and so to claim viral evolution / arms race as a driver of their evolution is very risky unless you show the exact same genes in the VIP set are under selection and the same genes in NCS-treatment etc. Also you would have match the evolutionary time of these genes (tmrca) with the age of the DNA pathogens found in bats, to really claim this. So be cautious with the wording.

We thank the reviewer for their comments and have attempted to carefully word this section to avoid potential pitfalls. To determine if viruses were the predominant cause of DNA VIP evolution, we compared the enrichment for selection in all VIPs to those with experimentally demonstrated proviral/antiviral activity. Enrichment was only shown for these VIPs with experimentally characterized proviral/antiviral activity (Fig 4B, C), strongly implicating that their evolution is due to viral pressures. We have clarified this in the main text. The description of VIP adaptation being driven by host-virus interaction is highly consistent with conclusions in the literature, and thus we feel that it is a reasonable claim (see eg. Ostridge et al. 2025. *Science*, *387*(6730), Daugherty and Malik. 2012. *Annual Review Genetics*. 46:677-700.)

For teh RICR there are some conflicting data that are complete glossed over. Yumanensis has quite a higher end RICR yet a lower longevity quotient - why does it sit up close to myotis myotis? Also auricalis is missing and some others like thysanodes should be much higher shouldn't it? It would be good to see all the myotis highlighted here and consistently use all the myotis genomes you added. How to explain the inconsistencies? particularly when the RICR score is used to justify myotis having selected genes for longevity. Similarly, thysanodes/lucifigus etc show more cancer pathway genes (5E) yet volans etc do not? so is the % of cancer genes are valid point to make?

In the interest of clarity and given our results in Figure 6, we have elected to remove our RICR analyses, and instead motivate our analysis of the percentage of cancer pathway enrichment on the basis of lifespan, leading into our findings suggesting pleiotropy between DNA damage repair and DNA VIPs. See also response below to reviewer 4.

I like the raincloud plot in the supplemental but it sees these strongly-selected genes are somewhat different to the NCS/treatment data.

We did not intend for the gene labels in Figure 6, Extended Data Figure 6, and Extended Data Figure 7 to highlight the same sets of genes; the motivations underlying these gene labels were distinct. We chose to highlight in Extended Data Fig 6E genes associated with pathways that others have examined related to bat biology in the literature, such as insulin signaling, iron metabolism, and SERPIN-family genes. Meanwhile, in Figure 6

and Extended Data Fig 7A we highlighted the top differentially expressed genes based on NCS treatment.

in the summary you link DNA virus adaptations to cancer resistance etc (yet there was no real difference in DNA virus evolution between myotis vs non-myotis bats, yet you still claim there is a difference in RICR for myotis, specifically ?）. These two points do not align. the comment on DNA transposons needs to be clarified due to the differences in different transposons between bats.

We apologize if we implied that *Myotis* is the only group of bats demonstrating these patterns. Indeed, we would like to highlight the opposite in this manuscript: that agonistic pleiotropy has likely played a fundamental role in shaping the unique biology of bats. Furthermore, we have edited our concluding paragraph to stress that *Myotis* serves as a useful model for exploring this phenomenon in bats using functional evolutionary genomics.

Overall the data itself is pretty clear and presented accurately but some of the conclusions I feel are strecthed to far from the data actual represents. This paper has generated some beautiful genomes and there is clearly a difference in the pressure of which genes to evolve (e.g. DNA sensing.repair machinery) - but this was kind of already known for bats and it is not a myotis-specific trend that has been pulled out. The PKR section is interesting though barely mentioned in the discussion. Gene duplication is an interesting trend that needs further observation. PKR itself is incredibly important for cell stress, stress granule formation, control of metabolism, scaffolding for NFKB, inflammasome activation and sterile inflammation (independent of any virus) - any of these pressures may have triggered gene duplication and none of this is mentioned.

We agree with the reviewer that PKR is a strong example of a pleiotrophic gene, and that this fact was not highlighted in the discussion. We have now added text to the discussion highlighting its role as not only an innate immune gene, but also a gene implicated in various aspects of aging and inflammation.

We would like to emphasize that most studies on selection in bats highlight the importance of either aging and cancer or immunity, and the few that discuss both treat them as two unrelated phenomena. Our manuscript instead, highlights the significant degree to which genes *at the intersection* of these two processes are enriched in these selection scans. As discussed in other responses, we have also made several changes to the text to highlight that this phenomenon is not unique to *Myotis*, but rather that *Myotis* are a valuable model system for studying this phenomenon.

Overall, we would like to underscore our gratitude to the reviewer for their perspectives and the points that they have brought up, as we feel they have greatly contributed to the clarity of this manuscript.

Referee #3 (Remarks on code availability):

The usage of ASBRel, cafe, busted etc seems quite normal. assembly and annotation seems quite logical and similar to methods I have used before.

Referee #4 (Remarks to the Author):

I am reviewing this paper for the second time. I think this new version of the manuscript present some very solid results in addition to publish the sequencing of 8 new bat genomes (an important resource for new studies). So, this paper is definitely of high quality and should be published in a high-ranking journal. However, I still think that the approaches used and results are not novel enough for publishing in a journal such as Nature. I feel that this paper is a broad examination of the very interesting biology and genetic of Myotis, opening a lot of research avenues for future papers and studies, but in-depth examination of the mechanisms at play is essential, in my point of view, to publish in Nature. For example, while the results obtained using the primary cell cultures are interesting, many more experiments are now needed to understand the characteristics of the DNA damage response in these species and how it might confer an increased resistance to cancer or not. This aspect is also not put into perspective in the discussion.

> We appreciate that the reviewer considers this manuscript to be interesting, and that the work herein opens the door to a number of new avenues of research across disciplines. We believe that this work presents a number of important conclusions that are of interest to an interdisciplinary readership. Our study uncovers new ties between aging, cancer resistance, and virus response in bats thus contributing both to our understanding of the etiology of the many extraordinary phenotypes found in Chiroptera and thus provides a new perspective on the study of evolution of aging and longevity across the tree of life. We have made edits to the discussion and conclusion to highlight the exciting future work enabled by our study.

See minor comments below.
Abstract: I still think that the last sentence of the abstract is really weak and this papers should have a much more important conclusion/perspective than what is stated in this last sentence.

> An updated concluding sentence of the abstract reads: “Together, our results suggest that bats’ remarkable longevity and immunity are linked through pleiotropic adaptations against viruses and aging-related disease.”

A third PKR haplotype - H3 - with three tandem duplicates of PKR (PKR1, PKR2, and a third copy) present only in M. californicus have been detected in this study. Then when the functional

impact of the duplicates' co-expression has been investigated, only PKR1 and PKR2 have been studied. Can the authors explain why this third copy has not been further studied? The rationale for studying PKR more than another gene is not super clear to me.

> We focused on PKR1 and PKR2 because they are the result of a duplication event that likely occurred at the base of *Myotis*, thus contributing to phenotypes across this clade. The third copy was unexpectedly found in *M. californicus* and appears to represent a very recent duplication event. Indeed, this H3 haplotype seems to result from a PKR1 duplication that is highly similar to PKR1, thus is likely to have a similar function. We thus focussed on PKR1 and PKR2 and hope future work can clarify the impact of this novel gene duplicate. The rationale for studying PKR was best put by Reviewer 3: *"PKR itself is incredibly important for cell stress, stress granule formation, control of metabolism, scaffolding for NFKB, inflammasome activation and sterile inflammation."* Furthermore, the fact that PKR is a trans-species polymorphism suggests it's evolution is shaped by complex selective forces. Finally, as put by reviewer #1, the observation of the trans-species PKR duplication polymorphism is "an impressive demonstration of the potential of haplotypic resolved genomes."

L260-261 : This is the theory and it has to clearly stated. Maybe, within species evidence should be cited ?

> We have edited this sentence to more explicitly refer to the underlying theory - the multistage theory of carcinogenesis - and added language and citations to show examples of studies within humans and other representative species to complement the analyses shown within larger, pan-mammal and pan-vertebrate studies.

L277-279: "This is consistent with recent pan-mammal and pan-vertebrate studies of cancer risk, which found that bats were present among the 20 species with the lowest rates of neoplasia, and not at all among the 20 species with the highest rates of neoplasia.". I still disagree with this statement. I still think that the data published so far are showing that the prevalence of cancer in bats is extremely variable between species. The fact that none of the bat species is among the 20 species with the highest prevalence of the study does not mean that bats can be considered as cancer-resistant.

> In the interest of clarity and given our results in Figure 6, we have elected to remove our RICR analyses. Instead, we have moved many of our comments and observations about cancer in bats to the discussion. We highlight the need for alternative approaches to longitudinal cancer prevalence studies in bats, as only a few species from Yinpterochiroptera (e.g. Rousettus aegypticus and Pteropus rodrigensis) have such data available. Due to the difficulties in captive rearing of most bat species and their exceptional lifespans in the wild, obtaining accurate cancer prevalence data from *Myotis* and other Yangochiropterans represents a significant challenge with no immediate solutions.

The first paragraph of the discussion is trying to convince the readers that collecting wing punches to establish primary fibroblast cell cultures is a revolutionary approach but I am clearly not convinced. This is a basic methodology in this field!

We do not make the claim that we are the first group to generate primary fibroblasts from wing punches - however, our approach to use primary cells to create near-complete genome assemblies in bats is novel, and has been a long-standing subject of discussion and debate within consortia such as Bat1K. This debate motivated our comparisons of cell-line-based and tissue-based genome assemblies in Supplementary Figure 1. Considering the ethical issues with euthanizing endangered species for genome assembly and annotation, and the logistical difficulties of sampling bats and other species world-wide, our results support that this should be a standard approach for the field.

L364-365: "We find that Myotis demonstrates an increased rate of change in lifespan given body size compared to other mammals." This is actually not true given that "We find that while non-bat mammals experience an average 0.159% increase in lifespan per 1% increase in body size, bats experience a 0.223% increase in lifespan years per 1% increase in body size, though these rates were not significantly different after phylogenetic correction" L254-255.

We note that the first sentence refers to *Myotis* specifically while the second refers to bats as total clade. We have edited the text to clarify: *We find that many extant and ancestral Myotis species have experienced some of the fastest rates of change in lifespan seen among mammals, despite minor changes in body size.*
